# East Asian Herbal Medicine to Reduce Primary Pain and Adverse Events in Cancer Patients : A Systematic Review and Meta-Analysis With Association Rule Mining to Identify Core Herb Combination

**DOI:** 10.3389/fphar.2021.800571

**Published:** 2022-01-17

**Authors:** Hee-Geun Jo, Jihye Seo, Seulki Choi, Donghun Lee

**Affiliations:** ^1^ Department of Bioinformatics and Statistics, Graduate School of Korea National Open University, Seoul, South Korea; ^2^ Department of Obstetrics and Gynecology, Se-Myung University Korean Medicine Hospital, Jecheon-si, South Korea; ^3^ Department of Herbal Pharmacology, College of Korean Medicine, Gachon University, Seongnam, South Korea

**Keywords:** East Asian herbal medicine, cancer pain, complementary and alternative medicine, systematic review, meta-analysis, association rule mining

## Abstract

**Objective:** Cancer pain is an important factor in cancer management that affects a patient’s quality of life and survival-related outcomes. The aim of this review was to systematically evaluate the efficacy and safety of oral administration of East Asian herbal medicine (EAHM) for primary cancer pain and to explore core herb patterns based on the collected data.

**Methods:** A comprehensive literature search was conducted in 11 electronic databases, namely, PubMed, Cochrane Library, Cumulative Index to Nursing & Allied Health Literature, EMBASE, Korean Studies Information Service System, Research Information Service System, Oriental Medicine Advanced Searching Integrated System, Korea Citation Index, Chinese National Knowledge Infrastructure Database (CNKI), Wanfang Data, and CiNii for randomized controlled trials from their inception until August 19, 2021. Statistical analysis was performed in R version 4.1.1 and R studio program using the default settings of the meta-package. When heterogeneity in studies was detected, the cause was identified through meta-regression and subgroup analysis. Methodological quality was independently assessed using the revised tool for risk of bias in randomized trials (Rob 2.0).

**Results:** A total of 38 trials with 3,434 cancer pain patients met the selection criteria. Meta-analysis favored EAHM-combined conventional medicine on response rate (risk ratio: 1.06; 95% CI: 1.04 to 1.09, *p* < 0.0001), continuous pain intensity (standardized mean difference: −1.74; 95% CI: −2.17 to −1.30, *p* < 0.0001), duration of pain relief (standardized mean difference: 0.96, 95% CI: 0.69 to 1.22, *p* < 0.0001), performance status (weighted mean difference: 10.71; 95% CI: 4.89 to 16.53, *p* = 0.0003), and opioid usage (weighted mean difference: −20.66 mg/day; 95% CI: −30.22 to −11.10, *p* < 0.0001). No significant difference was observed between EAHM and conventional medicine on response rate and other outcomes. Patients treated with EAHM had significantly reduced adverse event (AE) incidence rates. In addition, based on the ingredients of herb data in this meta-analysis, four combinations of herb pairs, which were frequently used together for cancer pain, were derived.

**Conclusion:** EAHM monotherapy can decrease adverse events associated with pain management in cancer patients. Additionally, EAHM-combined conventional medicine therapy may be beneficial for patients with cancer pain in increasing the response rate, relieving pain intensity, improving pain-related performance status, and regulating opioid usage. However, the efficacy and safety of EAHM monotherapy are difficult to conclude due to the lack of methodological quality and quantity of studies. More well-designed, multicenter, double-blind, and placebo-controlled randomized clinical trials are needed in the future. In terms of the core herb combination patterns derived from the present review, four combinations of herb pairs might be promising for cancer pain because they have been often distinctly used for cancer patients in East Asia. Thus, they are considered to be worth a follow-up study to elucidate their actions and effects.

**Systematic Review Registration:** https://www.crd.york.ac.uk/prospero/, identifier CRD42021265804

## 1 Introduction

Pain is an important factor influencing clinical outcomes in the medical management of cancer. Recent literature on the prevalence of cancer pain reports that pain is observed in more than one-third of the patients, that is, 60% of patients with cancer complain of pain (van den Beuken-van Everdingen et al., 2007; van den Beuken-van Everdingen et al., 2016). Cancer pain should not be overlooked in that it not only affects a patient’s quality of life but also affects the patient’s survival-related prognosis in the case that severe pain is not well-managed (Quinten et al., 2009; Beck et al., 2010; Ciucă and Băban, 2017). Although clinicians’ awareness of cancer pain is gradually improving, it has been reported that about one-third of cancer survivors do not have access to proper management (Greco et al., 2014). In addition to this, a significant number of patients still suffer from pain after completing curative treatment (van den Beuken-van Everdingen et al., 2007). Therefore, preparing a more effective and safer treatment strategy for cancer pain is an urgent task in clinical research above all else.

Currently, the WHO Analgesic Ladder is widely used as a framework for managing cancer pain. According to this recommendation, drugs ranging from over-the-counter analgesics to strong opioids can be administered sequentially as the severity of pain increases (Vargas-Schaffer, 2010). However, a large number of patients complain of severe pain that does not respond to treatment even after receiving opioids (Anderson et al., 2000). Because the etiology of cancer-causing pain is very diverse, it is difficult to consistently predict the effect of individual interventions on major outcomes of patients, such as the intensity of pain and functional status. Meanwhile, concerns of medical consumers about opioid administration due to the continuous increase in accidental prescription opioid overdose or patients’ financial problems are also pointed out as important barriers (Calcaterra et al., 2013; Kwon, 2014).

In this context, studies on various integrative therapies that can be used as therapeutic alternatives or to increase patient compliance with first-line pharmacologic treatment for cancer pain have been actively conducted recently (Deng, 2019). In particular, herbal medicine has been widely used as an intervention to relieve pain caused by various diseases for a long time in East Asian countries such as Korea, Taiwan, Japan, and China (Chen HY. et al., 2014; Lin PH. et al., 2016; Arai et al., 2020; Wang and Meng, 2021). Recently, a number of clinical and experimental studies on various problems caused by cancer have been reported based on the scientific methodological approach for East Asian herbal medicine (EAHM) (Lin et al., 2019; Tsai et al., 2019; Kim et al., 2020). Several systematic reviews have already been reported to explore the relieving effect of cancer pain (Wang S.-J. et al., 2013; Lee et al., 2015, 2019; Wang et al., 2019, 2020). Nevertheless, evidence related to the efficacy of EAHM for cancer pain, in general, is still insufficient. More RCTs have been additionally conducted thanks to the quantitative expansion of EAHM-related scientific research in recent years. Thus, studies that reflect these results need to be continued. On the other hand, previous reviews for EAHM comprehensively dealt with several types of EAHM formulas, including multiple herbal ingredients, unlike reviews on conventional medicine (CM) with a single dose and composition. For this reason, it is difficult to identify which of the much herb-related information reflected in the review is useful for clinicians or drug discovery. In addition, there are various methodological flaws, such as no limitation on the route of EAHM administration, insufficient analysis of adverse events, and a mixture of target diseases.

Therefore, we set the following research objectives to provide meaningful evidence to clinicians by comprehensively reviewing the efficacy and safety of EAHM for cancer pain and to explore useful hypotheses for drug discovery: 1) systematic literature review on the efficacy and safety of overall oral EAHM is conducted, focusing on the improvement of pain intensity and response rate of cancer pain excluding secondary pain caused by anti-cancer treatment; 2) Apriori algorithm-based association rule mining is performed on the herb data collected in this review to discover the core herb pattern.

## 2 Methods

This study was conducted in accordance with the Preferred Reporting Items for Systematic Reviews and Meta-Analysis 2020 statement (Page et al., 2021). The protocol of this systematic review was registered in PROSPERO (Registration Number: CRD42021265804, available from https://www.crd.york.ac.uk/prospero/display_record.php?ID=CRD42021265804). The procedure for this review has also been published in a scientific journal for public reading (Jo and Lee, 2021).

### 2.1 Search Strategy

Randomized controlled trials (RCTs) that evaluated the efficacy of EAHM for cancer pain were searched in the following 11 electronic databases from their inception until August 19, 2021: four English databases (PubMed, Cochrane Library, Cumulative Index to Nursing & Allied Health Literature (CINAHL), and EMBASE), four Korean databases (Korean Studies Information Service System (KISS), Research Information Service System (RISS), Oriental Medicine Advanced Searching Integrated System (OASIS), and Korea Citation Index (KCI)), two Chinese databases (Chinese National Knowledge Infrastructure Database (CNKI) and Wanfang Data), and one Japanese database (CiNii). At the time of preparing the protocol for this review, the search for the Wanfang Data database was not planned. However, in the process of researching the literature, more comprehensive data collection was required; hence, a search target database was added. The following Boolean format was used for the search: (Pain[MeSH] OR Pain*[TIAB] OR analgesia OR analges* OR nocicept* OR neuroapth*) AND (“Cancer pain”[TIAB] OR “Cancer patient”[TIAB] OR “Cancer patients”[TIAB] OR Neoplasms[MeSH] OR Neoplasms*[TI] OR Cancer*[TI] OR Tumor*[MeSH] OR Tumor*[TI] OR Carcinoma[MeSH] OR Carcinoma*[TI] OR Adenocarcinoma[MeSH] OR Adenocarcinoma*[TI] OR adenomatous[TI] OR Lymphoma[MeSH] OR lymphom*[TI] OR lymphedema*[TI] OR Sarcoma[MeSH] OR Sarcoma*[TI] OR “‘Antineoplastic agents”[MeSH] OR antineoplas*[TI] OR ((adenom*[TI] OR adenopath*[TI]) AND malignant*[TI]))) AND (“Plants, Medicinal”[MeSH] OR “Drugs, Chinese Herbal”[MeSH] OR “Medicine, Chinese Traditional”[MeSH] OR “Medicine, Kampo”[MeSH] OR “Medicine, Korean Traditional”[MeSH] OR “Herbal Medicine”[MeSH] OR “Prescription Drugs”[MeSH] OR “traditional Korean medicine”[TIAB] OR “traditional Chinese medicine”[TIAB] OR “traditional oriental medicine”[TIAB] OR “Kampo medicine”[Title/abstract] OR herb*[TIAB] OR decoction*[TIAB] OR botanic*[TIAB]). In Korean, Chinese, and Japanese databases, these search terms were appropriately modified to perform a search. Detailed search strategies are explicated in Supplementary Figure S1.

### 2.2 Study Selection

#### 2.2.1 Type of Studies

Only RCTs evaluating the efficacy and safety of oral administration of EAHM for cancer pain were included. There were no restrictions on language and publication time. Some studies were excluded if they met the following criteria: 1) not RCT or quasi-RCT; 2) inappropriate or no control group; 3) unrelated to cancer pain; 4) animal experiments; 5) case reports or review; 6) not published in scientific peer-reviewed journals, including postgraduate theses or dissertations.

#### 2.2.2 Type of Participants

Trials were considered eligible for inclusion if they were conducted in patients with cancer pain, with no restriction on age, gender, or race. Studies that recruited patients' secondary cancer-related pain caused by other anticancer therapies like chemotherapy or surgery were excluded since this review focused on primary cancer pain.

#### 2.2.3 Type of Interventions

RCTs that compared EAHM as the active intervention in the treatment group versus placebo or conventional medicine (CM) in the control group were included. RCTs that tested EAHM-combined CM (ECCM) versus CM alone were also considered. All forms of EAHM such as decoction, granule, and capsule for the management of cancer pain were included. There were no restrictions on the dose and duration of treatment for EAHM, but the mode of delivery was limited to oral intake. Studies in which East Asian medical interventions such as acupuncture, massage, or non-drug therapy were only combined in the treatment group were excluded. Studies in which the comparators included other EAHMs were excluded. In addition, studies that could not confirm the composition of individual ingredients and herbs of the utilized EAHM prescription were also excluded.

#### 2.2.4 Type of Outcome Measures

The primary outcome for cancer pain patients was the remission rate for each group measured using the Verbal Rating Scale (VRS), Numerical Rating Scale (NRS), and Visual Analogue Scale (VAS). However, most included studies reported remission rates of complete remission (CR), partial remission (PR), mild remission (MR), and no remission (NR) as CR + PR/all patients. If the remission rate reported by the individual studies is used as is, there is a concern that an outcome lacking consistency may be reported because there is a difference in the categorization criteria for each study. Therefore, the proportion of patients who had remission in each group was used as the response rate by converting the data of the study in which all detailed category information was reported in this review. In addition, individual continuous pain intensity outcomes such as NRS and VAS were also adopted as primary outcomes. Secondary outcomes including duration of pain relief, performance status, and opioid usage were used. In the case of performance status, only outcomes measured by the Karnofsky scale, which is used for cancer patients to access the ability to do ordinary works without impairment, were reflected in the results. Meanwhile, in order to evaluate the safety of the intervention for cancer patients, the incidence of adverse events (AEs) was also included as a secondary endpoint.

#### 2.2.5 Data Extraction

According to the above-mentioned search strategy, the titles and abstracts of potentially eligible studies were independently screened by two investigators (HGJ and JS). Afterward, a full-text review was performed based on the inclusion and exclusion criteria. Subsequently, information on the included studies was extracted independently by two reviewers (HGJ and JS). The following information was collected: title, first author’s name, publication year, sample size, participant age, sex distribution, study design, type of cancer, interventions in the treatment and control groups, treatment duration, outcome measures, reported adverse event, and composition with the dosage of EAHM. Any discrepancy was discussed with the third author (DL).

#### 2.2.6 Methodological Quality Assessment

The methodological quality of each included study was evaluated independently by two investigators (HGJ and JS) according to the revised tool for risk of bias in randomized trials, Rob 2.0 (Sterne et al., 2019). It is comprised of five domains: bias arising from the randomization process, bias due to deviations from intended interventions, bias due to missing outcome data, and bias in the selection of the reported results. Methodological quality was assessed on three levels: “high risk of bias,” “low risk of bias,” and “some concerns.” Disagreements between the two investigators were resolved with the help of the third author (DL).

#### 2.2.7 Statistical Analysis

##### 2.2.7.1 Evidence Synthesis

Evidence synthesis of the included studies with available data was performed by calculating the effect size and 95% CI using only the random-effects model. Heterogeneity was considered statistically significant when the *p*-value based on the χ^2^ test was less than 0.10 or I^2^ was 50% or more. Two-sided *p* < 0.05 was considered statistically significant. Statistical synthesis of individual research results was performed in R version 4.1.1 and R studio program (Version 1.4.1106, Integrated Development for R. RStudio, PBC, Boston, MA) using the default settings of the meta-package (Lortie and Filazzola, 2020). In this review, in order to effectively reveal the exact value of the effect size without relying only on the *p* < 0.05 significance threshold in the interpretation of the primary outcome synthesis result, a drapery plot was additionally illustrated along with the forest plot (Rücker and Schwarzer, 2021). The studies were grouped according to the type of intervention, such as East Asian herbal medicine (EAHM) and East Asian herbal medicine combined conventional medicine (ECCM), and comparator, such as conventional medicine (CM). Summary relative risk (RR) and 95% confidence interval (CI) were calculated for the response rate. Standardized mean difference (SMD) and 95% CIs were calculated for continuous pain intensity and duration of pain relief. Mean difference (MD) and 95% CIs were calculated for opioid usage and performance status. AEs were calculated using the odds ratio because the probability of occurrence of an event is significantly lower than that of other outcomes, and it is necessary to estimate a causal relationship. In order to distinguish publication bias, a contour-enhanced funnel plot was used for the outcome that included the most studies (Peters et al., 2008). For the asymmetry on the visually confirmed funnel plot, Egger’s test (Egger et al., 1997) and Begg’s test (Begg and Mazumdar, 1994) were additionally performed to specifically confirm the existence of publication bias.

##### 2.2.7.2 Association Rule Mining

By analyzing the constituent herb data of EAHM collected from the included study, the potential association rules of core herb combinations were explored. Before proceeding with this analysis, preliminary information for data mining was extracted by first analyzing the frequency of individual herbs. The R studio program (Version 1.4.1106, Integrated Development for R. RStudio, PBC, Boston, MA) was used for the Apriori association rule analysis and plot production. A data fit was done using the “arules” package in R studio (Hahsler et al., 2005). The function of the R package “arulesViz” was applied to generate graphical presentations according to the results (Hahsler, 2017). Mining of frequent hub itemsets and association rules was performed according to the Apriori algorithm method for discovering meaningful relationships between variables in a large database (Agrawal et al., 1993). Through this, it is possible to identify the elements composing the data and the relationship between the elements, and it is being used in various types of medical research aimed at predicting the characteristics of interventions (Leem et al., 2018; Hsieh et al., 2020; Lin et al., 2021).

In the Apriori algorithm, support, confidence, and lift are the main metrics for measuring association. A rule is defined as an expression X⇒Y, where X, Y ⊆ I and X∩Y = ∅. The herb X and herb Y are called antecedent (left-hand side, LHS) and consequent (right-hand side, RHS) of the rules. Association rules are rules that surpass researcher-specified minimum support and minimum confidence thresholds. The support, supp (X), of an itemset X is a measure of importance defined as the proportion of transactions in the dataset which contain the itemset. The confidence of a rule is defined as conf (X⇒Y) = supp (X∪Y)/supp (X), measuring the likelihood of seeing herb Y in a transaction containing herb X. An association rule X⇒Y needs to satisfy supp (X∪Y) ≥ σ and conf (X⇒Y) ≥ δ, where σ and δ are the minimum support and minimum confidence, respectively. Confidence can be interpreted as an estimate of the probability P(Y|X), which is the probability of finding the RHS of the rule in transactions, given that these transactions also contain the LHS. Lift of a rule is defined as lift (X⇒Y) = (supp (X∪Y)/supp (X)). Support is the measure to evaluate the usefulness of the association rule and is the proportion of prescriptions containing a specific herb combination pattern in the total EAHM prescription. When the confidence is close to 1, herb A and herb B are irrelevant because they are close to independence in probability. Meanwhile, if the lift value is large, the relationship between herb A and herb B is interpreted as a strong correlation. In this study, the association rules were identified based on the minimum values for support and confidence being 20 and 80%, respectively. Among them, the core herb combination patterns showing the most distinct association and its constituent herbs were searched.

#### 2.2.8 Quality of Evidence According to Outcome Measurements

The overall quality of evidence for each outcome was evaluated according to the Grading of Recommendations Assessment, Development, and Evaluation (GRADE) pro (Guyatt et al., 2008). The GRADE assessment evaluates the overall quality of evidence in four levels: very low, low, moderate, and high. The level of evidence is lowered according to certain factors, such as the risk of bias, inconsistency, indirectness, imprecision, and publication bias, respectively.

## 3 Results

### 3.1 Study Identification

**FIGURE 1:**
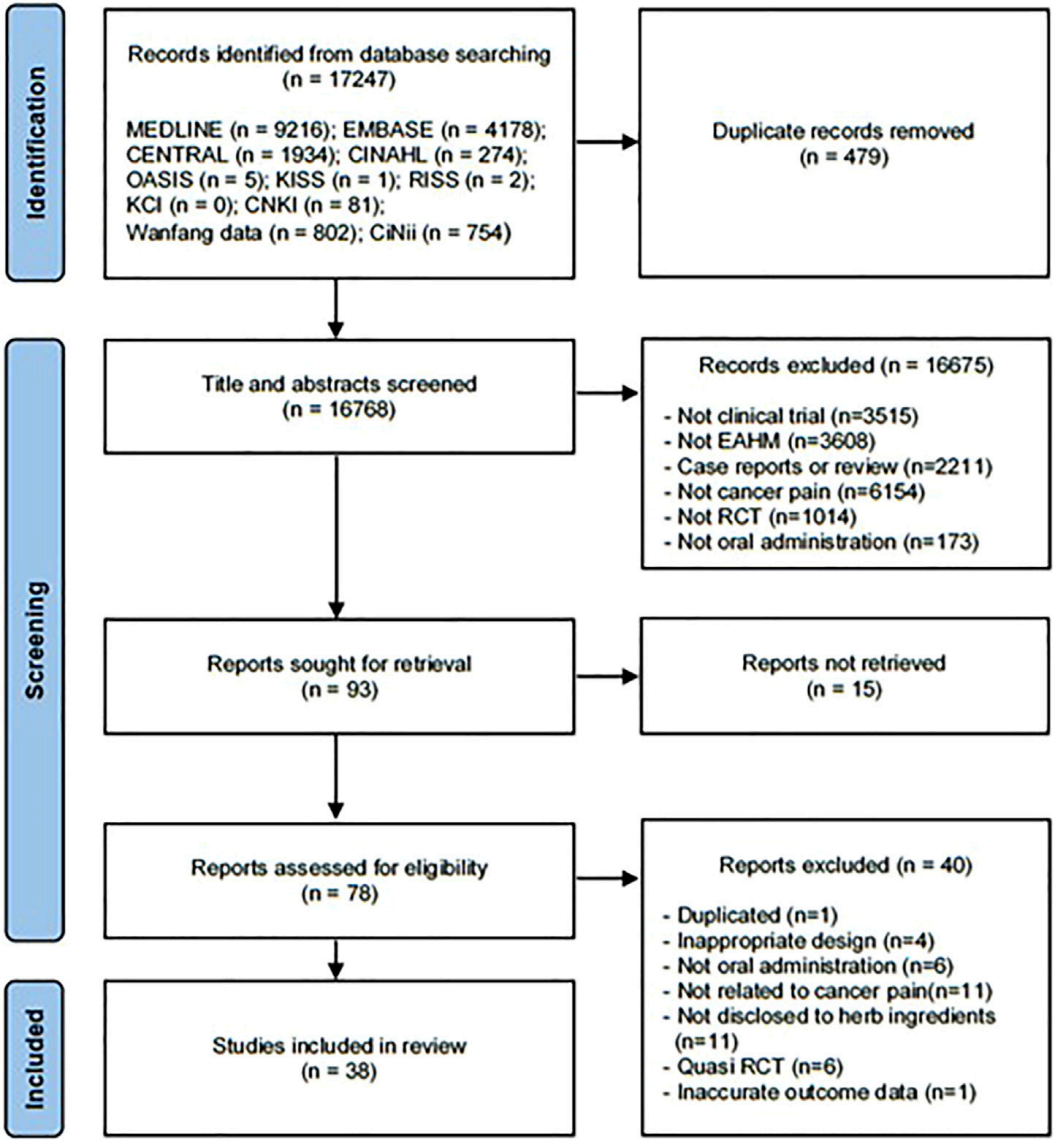
PRISMA 2020 flow diagram.

Based on search strategy, a total of 17,247 potentially relevant articles were identified by electronic search in the 11 databases. After the removal of 479 duplicates, 16,768 reports were retrieved. After screening for titles and abstracts, 16,675 articles that met at least one of the exclusion criteria were removed. A full-text assessment was performed on the remaining 78 studies, and 40 articles were excluded for the reasons listed in Figure 1. The bibliographic information of documents excluded after the full-text review is presented in Supplementary Figure S2. Finally, a total of 38 eligible studies were included in this meta-analysis (Lin et al., 2001; Zhang, 2001; Li et al., 2002; Ma et al., 2003; Chen, 2004; Chen et al., 2005; Wu et al., 2005; Cao and Xu, 2006; Zhang et al., 2006; Chen, 2009; Hao, 2009; Zhai et al., 2009; Zhang, 2009; Zhang et al., 2009; Cai, 2010; Li et al., 2010; Fu, 2011; Wang et al., 2011; Zhou, 2011; Cheng et al., 2012; He, 2012; Meng, 2012; Jiang et al., 2013; Wang and Chen, 2013; Chen H. et al., 2014; Liu and Zhou, 2014; Wan et al., 2014; Song et al., 2015; Li et al., 2017a; Li et al., 2017b; Chen et al., 2017; Bao, 2018; Dong et al., 2018; Miu and Quan, 2018; Ouyang, 2018; Liu, 2020; Yang, 2020; Liang et al., 2021). The screening process is summarized in the PRISMA 2020 flow diagram (Figure 1).

### 3.2 Study Characteristics

The basic characteristics of the 38 included studies are summarized in Table 1. Only one study was published in English and the rest were all published in Chinese. All studies were conducted in China. In general, 3434 patients with cancer pain were included. The sample size ranged from 30 to 320 participants. In the treatment groups, 28 studies used ECCM (Lin et al., 2001; Chen et al., 2005; Cao and Xu, 2006; Zhang et al., 2006; Chen, 2009; Hao, 2009; Zhang et al., 2009; Cai, 2010; Li et al., 2010; Fu, 2011; He, 2012; Jiang et al., 2013; Wang and Chen, 2013; Chen H. et al., 2014; Liu and Zhou, 2014; Wan et al., 2014; Li et al., 2017b; Chen et al., 2017; Bao, 2018; Dong et al., 2018; Miu and Quan, 2018; Ouyang, 2018; Liu, 2020; Yang, 2020; Liang et al., 2021), and 10 studies used EAHM alone (Zhang, 2001; Li et al., 2002; Ma et al., 2003; Wu et al., 2005; Zhai et al., 2009; Zhang, 2009; Wang et al., 2011; Zhou, 2011; Cheng et al., 2012; Meng, 2012). In terms of control conditions, all included studies used CM, such as WHO 3-step ladder, opioids, and other analgesics. Outcomes on the efficacy of EAHM were reported in all 38 included studies. Response rate was reported as the primary outcome measure in 37 studies (Lin et al., 2001; Zhang, 2001, 2009; Li et al., 2002, 2010, 2017a, 2017b; Ma et al., 2003; Chen, 2004, 2009; Chen et al., 2005, Chen H. et al., 2014, 2017; Wu et al., 2005; Cao and Xu, 2006; Zhang et al., 2006, 2009; Hao, 2009; Zhai et al., 2009; Cai, 2010; Fu, 2011; Wang et al., 2011; Zhou, 2011; Cheng et al., 2012; He, 2012; Meng, 2012; Jiang et al., 2013; Wang and Chen, 2013; Wan et al., 2014; Bao, 2018; Dong et al., 2018; Miu and Quan, 2018; Ouyang, 2018; Liu, 2020; Yang, 2020; Liang et al., 2021). Continuous pain intensity, another primary outcome measure, was reported in 12 studies (Ma et al., 2003; Zhai et al., 2009; Meng, 2012; Jiang et al., 2013; Song et al., 2015; Li et al., 2017a, 2017b; Dong et al., 2018; Miu and Quan, 2018; Liu, 2020; Yang, 2020; Liang et al., 2021). In terms of secondary outcome measures, duration of pain relief was observed in 9 studies (Lin et al., 2001; Wu et al., 2005; Cao and Xu, 2006; Chen, 2009; Li et al., 2017a; Li et al., 2017b; Dong et al., 2018; Ouyang, 2018). Performance status was observed in 7 studies (Chen et al., 2005, 2017; Li et al., 2017a, 2017b; Dong et al., 2018; Miu and Quan, 2018; Liang et al., 2021); opioid usage was observed in 3 studies (Li et al., 2017a; Li et al., 2017b; Dong et al., 2018). Adverse events were reported in 30 studies (Lin et al., 2001; Zhang, 2001; Li et al., 2002; Chen et al., 2005; Wu et al., 2005; Cao and Xu, 2006; Zhang et al., 2006; Zhai et al., 2009; Chen, 2009; Zhang, 2009; Zhang et al., 2009; Fu, 2011; Wang et al., 2011; Zhou, 2011; Cheng et al., 2012; He, 2012; Meng, 2012; Wang and Chen, 2013; Chen H. et al., 2014; Wan et al., 2014; Song et al., 2015; Li et al., 2017a; Li et al., 2017b; Bao, 2018; Dong et al., 2018; Miu and Quan, 2018; Ouyang, 2018; Yang, 2020; Liang et al., 2021).

**TABLE 1:** Characteristics of included studies.

First author (Year)	Type of cancer	Trial design	Number of participants (male/female); age (mean ± SD)		Interventions		Outcome index (intergroup differences *p*-value)	Course of treatment	Adverse event (case/symptom)
			Trial	Control	Trial	Control			
Lin et al. (2001)	Mixed (including esophageal cancer, gastric cancer, lung cancer, liver cancer, breast cancer, rectal cancers)	RCT	30 (19/11); 57.23 ± 14.62 years	30 (18/12); 55.81 ± 15.74 years	1) Jianwei niantong capsules (4 c, p.o., q.i.d.) 2) WHO 3-step analgesic ladder treatment: aspirin tablets (0.5 g, p.o., q.i.d.); tramadol capsules (50 mg, p.o., q.i.d.); meperidine tablets (50 mg, p.o., q.i.d.)	1) WHO 3-step analgesic ladder treatment: aspirin tablets (0.5 g, p.o., q.i.d.); tramadol capsules (50 mg, p.o., q.i.d.); meperidine tablets (50 mg, p.o., q.i.d.)	1) Response rate (*p* < 0.05) 2) Duration of pain relief (*p* < 0.01)	10 h	E: 18 cases (7 nausea, 6 dizziness, 3 constipation, 2 mild diarrhea) C: 45 cases (13 nausea, 8 vomiting, 6 dizziness, 18 constipation)
Zhang (2001)	Mixed (including gastric, liver, colon, lung, breast cancers)	RCT	82 (NR gender info); mean 62.4 years (both groups)	28 (NR gender info); mean 62.4 years (both groups)	1) Compound strynchnos capsule (1 c, p.o., t.i.d.)	1) Indomethacin suppositories (50 mg, p.r., b.i.d.)	1) Response rate (*p* > 0.05)	3 weeks	E: 5 cases (due to overdosage, 1 muscle stiffness, 4 dysesthesia of mouth) C: 10 cases (3 hepatic and renal dysfunction, 7 nausea with anorexia)
Li et al. (2002)	Mixed (including lung, gastric, gallbladder, colon, pancreatic, bladder, renal, ovarian, prostate cancers)	RCT	46 (60/24; both groups); range 46–64 years (both groups)	38 (60/24; both groups); range 46–64 years (both groups)	1) Tibetan medicine duyiwei (3 c, 0.3 mg/c, p.o., t.i.d.)	1) Indomethacin (25 mg, p.o., t.i.d.)	1) Response rate (*p* > 0.05)	3 days	E: 2 cases (2 nausea with stomach discomfort) C: 16 cases (14 gastrointestinal reactions, 2 dizziness with headache)
Ma et al. (2003)	Gastric cancer	RCT	31 (25/6); 53.1 years, range 28–79	31 (24/7); 52.8 years, range 25–75	1) Jiaweibaoankeli (9 g, p.o., t.i.d.)	1) Propoxyaminophen compound tablets (1 t, p.o., b.i.d.)	1) Response rate (*p* > 0.05) 2) Duration of pain relief (*p* > 0.05) 3) Pain intensity (*p* > 0.05) 4) Performance status (*p* > 0.05)	15 days	NR
Chen (2004)	Mixed (including liver, gastric, esophageal, pancreatic, colon, metastatic cancers)	RCT	73 in both groups (46/27) 51 years, range 13–82 years	51 in both groups (46/27) Only reports that there is no statistical difference between groups.	1) Shitong decoction 2) WHO 3-step analgesic ladder treatment: morphine sulfate controlled-release tablets (30–60 mg, p.o., b.i.d.); aspirin 0.6 g or indomethacin 25 mg (p.o., t.i.d.)	1) WHO 3-step analgesic ladder treatment: morphine sulfate controlled-release tablets (30–60 mg, p.o., b.i.d.); aspirin 0.6 g or indomethacin 25 mg (p.o., t.i.d.)	1) Response rate (*p* < 0.05)	1 week	NR
Chen et al. (2005)	Mixed (including lung, esophageal, gastric, colon, liver, pancreatic cancers)	RCT	25 (16/9); 54.60 ± 11.35 years	25 (17/8); 53.20 ± 10.28 years	1) Zhitong capsules (4 c, p.o., t.i.d.) 2) tramadol capsules; morphine surfate	1) WHO 3-step analgesic ladder treatment: indomethacin (25 mg, p.o., t.i.d.); tramadol capsules (100 mg, p.o., b.i.d.); morphine surfate (30 mg, p.o., b.i.d.)	1) Response rate (*p* < 0.05) 2) Performance status (*p* > 0.05)	3 days	E: 12 cases (3 constipation, 2 dizziness, 5 nausea, 2 drowsiness) C: 28 cases (3 stomach discomfort, 11 constipation, 2 dizziness, 7 nausea, 5 drowsiness)
Wu et al. (2005)	Mixed (including lung, esophageal, gastric, colon, liver, pancreatic, other type cancers)	RCT	30 (17/13); 58.23 ± 7.32 years	30 (18/12); 58.90 ± 5.18 years	1) Aitongping capsules (0.4 g, 4 c, p.o., t.i.d.)	1) Compound ciclofenac sodium and Codein tablets (40 mg, p.o., t.i.d.)	1) Response rate (*p* > 0.05)	1 week	E: 0 case C: 3 cases (1 nausea, 1 vomiting, 1 constipation)
Cao and Xu (2006)	Bone metastasis (including lung, prostate, breast, esophageal, nasopharyngeal, thyroid primary cancers)	RCT	41 (26/15); 59.82 years	41 (25/16); 57.36 years	1) Zhuanggu zhitong san decoction (200 ml, p.o., b.i.d.) 2) WHO 3-step analgesic ladder treatment: aspirin tablets (0.3 g, p.o., q.i.d.); tramadol capsules (50 mg, p.o., q.i.d.); meperidine tablets (50 mg, p.o., q.i.d.)	1) WHO 3-step analgesic ladder treatment: aspirin tablets (0.3 g, p.o., q.i.d.); tramadol capsules (50 mg, p.o., q.i.d.); meperidine tablets (50 mg, p.o., q.i.d.)	1) Response rate (*p* < 0.05) 2) Duration of pain relief (*p* < 0.01)	10 h	E: 28 cases (8 nausea, 7 vomiting, 6 dizziness, 7 constipation) C: 46 cases (13 nausea, 8 vomiting, 7 dizziness, 18 constipation)
Zhang et al. (2006)	Mixed (including lung, gastric, liver, esophageal, colon cancers)	RCT	41 (28/13); 56.2 ± 8.4 years	43 (25/18); 52.7 ± 9.5 y	1) EAHM formula for individual research (100 ml, p.o., b.i.d.) 2) Morphine hydrochloride sustained-release tablets (30 mg, p.o., t.i.d.)	1) Morphine hydrochloride sustained-release tablets (30 mg, p.o., t.i.d.)	1) Response rate (*p* < 0.05)	2 weeks	E: 2 cases (1 nausea and vomiting, 1 constipation) C: 4 cases (1 burning sensation of dorsal region, 3 nausea and vomiting)
Chen (2009)	Bone metastasis (no specific types of primary cancer reported)	RCT	35 (19/16); median 52 years, range 39–65 years	35 (18/17); median 53 years, range 40–66 years	1) Jiawei Shentong Zhuyu decoction (100 ml, p.o., b.i.d.) 2) Zoledronic acid with normal saline (4 mg, i.v., at least 2 times in 4 weeks)	1) Zoledronic acid with normal saline (4 mg, i.v., at least 2 times in 4 weeks)	1) Response rate (*p* > 0.05) 2) Duration of pain relief (*p* < 0.05)	8 weeks	E: 13 cases (8 fever, 4 nausea and vomiting, 1 myalgia) C: 21 cases (10 fever, 6 nausea and vomiting, 5 myalgia)
Hao (2009)	Bone metastasis (including lung, prostate, breast, esophageal, nasopharyngeal, thyroid primary cancers)	RCT	29 (13/16); NR	29 (13/16); NR	1) EAHM formula for individual research (150 ml, p.o., b.i.d.) 2) Zoledronic acid with 0.9% sodium chloride or 5% glucose 100 ml (4 mg, i.v., once every 3 wees)	1) Zoledronic acid with 0.9% sodium chloride or 5% glucose 100 ml (4 mg, i.v., once every 3 wees)	1) Response rate (*p* < 0.05)	30 days	NR
Zhai et al. (2009)	Mixed (including lung, liver, gastric, pancreatic, cervical, ovarian, rectal, colon, other type cancers)	RCT	89 (51/38); 56.92 years, range 18–86 years	80 (48/32); 56.83 years, range 28–89 years	1) Anti-cancer zhitong decoction (150 ml, p.o., b.i.d.)	1) Bucinnazine tablets (60 mg, p.o., t.i.d.)	1) Response rate (*p* < 0.05) 2) Pain intensity (*p* < 0.01) 3) Performance status (*p* < 0.01)	2 weeks	E: 2 cases (1 nausea, 1 stomach discomfort) C: 12 cases (5 nausea, 2 vomiting, 3 stomach discomfort, 1 excitation, 1 fatigue)
Zhang (2009)	Liver cancer	RCT	80 (42/38); range 31–68 years (both groups)	80 (42/38); range 31–68 years (both groups)	1) Tuqi powder (12 g, p.o., t.i.d.)	1) WHO 3-step analgesic ladder treatment: Tramadol capsules (50 mg, p.o., t.i.d.); Morphine sulfate tablets (10 mg, p.o., b.i.d.)	1) Response rate (*p* < 0.05)	2 weeks	E: 2 cases (No details reported) C: 14 cases (No details reported)
Zhang (2009)	Mixed (specific cancer type NR)	RCT	40 (29/11); 59 years, range 50–79 years)	40 (25/15); 57.6 years, range 49–81 years)	1) Wendan decoction (100 ml, p.o., b.i.d.) 2) Morphine sustained-release tablets (30 mg, p.o., b.i.d.)	1) Morphine sustained-release tablets (30 mg, p.o., b.i.d.)	1) Response rate (*p* > 0.05)	1 week	E: 2 cases (2 nausea and vomiting) C: 8 cases (8 nausea and vomiting)
Cai (2010)	Bone metastasis (including prostate, breast, lung, liver, renal, thyroid, colon, nasopharyngeal primary cancers)	RCT	40 (NR gender info); 52.1 y, range 42–70 years (both groups)	40 (NR gender info); 52.1 y, range 42–70 years (both groups)	1) Yanghe decoction (p.o.) 2) Zoledronic acid with normal saline 50 ml (4 mg, i.v., q.1.m.)	1) Zoledronic acid with normal saline 50 ml (4 mg, i.v., q.1.m.)	1) Response rate (*p* < 0.05)	4 weeks	NR
Li et al. (2010)	Esophageal cancer	RCT	20 (15/5); 58.90 ± 10.17 years	20 (14/6); 57.95 ± 6.75 years	1) Taohongsiwu decoction (p.o.) 2) Ondansetron with normal saline 10 ml (8 mg, i.v., 15 min before each chemotherapy)	1) Ondansetron with normal saline 10 ml (8 mg, i.v., 15 min before each chemotherapy)	1) Response rate (*p* > 0.05)	8 days	NR
Fu (2011)	Mixed (including lung, colon, gastric, liver, breast cancers, cholangioma)	RCT	64 (43/21); median 55 years, range 45–70 years (both groups)	64 (43/21); median 55 years, range 45–70 years (both groups)	1) Qigetongbu decoction (p.o., b.i.d.) 2) Fentanyl transdermal patches (4.2 mg, t.d., q.72.h.)	1) Fentanyl transdermal patches (4.2 mg, t.d., q.72.h.)	1) Response rate (*p* > 0.05)	15 days	E: 7 cases (2 constipation, 2 nausea and vomiting, 1 dizziness, 2 drowsiness) C: 31 cases (9 constipation, 10 nausea and vomiting, 8 dizziness, 4 drowsiness)
Wang et al. (2011)	Bone metastasis (including lung, prostate, breast, esophageal primary cancers)	RCT	35 (19/16); 55.7 years	35 (17/18); 56.2 years	1) EAHM formula for individual research (200 ml, p.o., b.i.d.)	1) Pamidronate disodium with normal saline 500 ml (60 mg, i.v., q.d.)	1) Response rate (*p* < 0.01)	30 days	E: 4 cases (4 diarrhea) C: 4 cases (1 vomiting, 2 fever, 1 hypocalcemia)
Zhou (2011)	Liver cancer	RCT	160 (109/51); range 31–68 years (both groups)	160 (109/51); range 31–68 years (both groups)	1) Modified Tuqi powder (12 g, p.o., t.i.d.)	1) WHO 3-step analgesic ladder treatment: tramadol capsules (50 mg, p.o., t.i.d.); morphine sulfate controlled-release tablets (10 mg, p.o., b.i.d.)	1) Response rate (*p* < 0.05)	1 week	E: incidence of adverse events was 4.8% (nausea, vomiting, dizziness, drowsiness, etc.) C: incidence of adverse events was 36.8% (nausea, vomiting, dizziness, drowsiness etc.)
Cheng et al. (2012)	Bone metastasis (including breast primary cancer)	RCT	15 (NR gender info); 42.00 ± 12.32 years, range 27–58 years (both groups)	15 (NR gender info); 42.00 ± 12.32 years, range 27–58 years (both groups)	1) Baizhu fuzi decoction (200 ml, p.o., b.i.d.)	1) Zoledronic acid with 5% glucose 250 ml (4 mg, i.v., q.1.m.)	1) Response rate (*p* < 0.05)	16 weeks	E: 0 case C: 5 cases (2 fever with chilling sign, 1 headache, 1 muscular pain, 1 anorexia, 1 diarrhea)
He (2012)	Bone metastasis (including lung, breast, gastrointestinal, liver, prostate, cervical primary cancers)	RCT	28 (18/10); 58 years, range 46–76 years	28 (20/8); 56 years, range 47–70 years	1) EAHM formula for individual research (p.o.) 2) Zoledronic acid with normal saline 1000 ml (4 mg, i.v., q.1.m.)	1) Zoledronic acid with normal saline 1000 ml (4 mg, i.v., q.1.m.)	1) Response rate (*p* < 0.05)	8 weeks	E: 5 cases (2 transient exacerbation of bone pain, 3 nausea and vomiting with anorexia) C: 12 cases (4 transient exacerbation of bone pain, 5 nausea and vomiting with anorexia, 2 fever, 1 facial eruption)
Meng (2012)	Bone metastasis (including lung primary cancer)	RCT	21 (10/11); 49 years, range 41–64 years (both groups)	21 (11/10); 49 years, range 41–64 years (both groups)	1) EAHM formula for individual research (150 ml, p.o., b.i.d.)	1) Zoledronic acid with normal saline 100 ml (4 mg, i.v., q.1.w.)	1) Response rate (*p*-value NR) 2) Pain intensity (*p*-value NR )	2 weeks	Incidence of adverse events (both groups): fever 6.5%, bone and joint pain 3.1%, gastrointestinal reaction 7.8%.
Jiang et al. (2013)	Colorectal cancer	RCT	32 (18/14); 53.2 ± 12.4 years	31 (18/13); 53.1 ± 12.8 years	1) EAHM formula for individual research (200 ml, p.o., b.i.d.) 2) Oxaliplatin with 5% glucose (135 mg, i.v., q.2.w.) 3) Folinic acid and calcium salt hydrate (200 mg, i.v., q.2.w.) 4) 5-Fluorouracil (400 mg, i.v., q.2.w.)	1) Oxaliplatin with 5% glucose (135 mg, i.v., q.2.w.) 2) Folinic acid and calcium salt hydrate (200 mg, i.v., q.2.w.) 3) 5-Fluorouracil (400 mg, i.v., q.2.w.)	1) Response rate (*p* < 0.05) 2) Pain intensity (*p* < 0.05)	8 weeks	NR
Wang S. J. et al. (2013)	Mixed (including lung, liver, gastric, pancreatic, esophageal, breast cancers)	RCT	40 (25/14); 41.2 ± 9.7 years	40 (29/11); 41.8 ± 8.6 years	1) Gexia zhuyu decoction combined Shixiao powder 2) WHO 3-step analgesic ladder treatment: non-opioids (aspirin); weak opioids (codeine); strong opioids (morphine)	1) WHO 3-step analgesic ladder treatment: non-opioids (aspirin); weak opioids (codeine); strong opioids (morphine)	1) Response rate (*p* = 0.025)	90 days	E: 0 cases C: 0 cases
Chen H. et al. (2014)	Mixed (including lung, liver, gastric, colon cancers)	RCT	50 (26/24); 62 ± 13 years	50 (28/22); 59 ± 15 years	1) Xuefu Zhuyu decoction (250 ml, p.o., b.i.d.) 2) Morphine sulfate sustained-release tablets (10–30 mg, p.o., b.i.d.)	1) Morphine sulfate sustained-release tablets (10–30 mg, p.o., b.i.d.)	1) Response rate (*p* < 0.05)	24 weeks	E: 43 cases (21 constipation, 11 nausea, 5 vomiting, 3 pruritus, 3 other symptom) C: 74 cases (35 constipation, 18 nausea, 6 vomiting, 7 pruritus, 8 other symptom)
Liu and Zhou (2014)	Gastric cancer	RCT	31 (23/8); 63.45 ± 11.51 years	31 (24/7); 62.85 ± 12.76y	1) Buqi Huoxue decoction (p.o.) 2) Pantoprazole with normal saline 100 ml (40 mg, i.v., q.d.) 3) Granisetron with 5% glucose 50 ml (3 mg, i.v., q.d.) 4) Oxaliplatin with 5% glucose 500 ml (20 mg, i.v., q.d.) 5) Tegafur with 5% glucose 500 ml (0.8 g, i.v., q.d.) 6) Calcium folinate with 5% glucose 250 ml (200 mg, i.v., q.d.)	1) Pantoprazole with normal saline 100 ml (40 mg, i.v., q.d.) 2) Granisetron with 5% glucose 50ml (3 mg, i.v., q.d.) 3) Oxaliplatin with 5% glucose 500 ml (20 mg, i.v., q.d.) 4) Tegafur with 5% glucose 500 ml (0.8 g, i.v., q.d.) 5) Calcium folinate with 5% glucose 250 ml (200 mg, i.v., q.d.)	1) Recurrence rate (*p* < 0.01) 2) Other analgesics usage (*p* < 0.05)	12 weeks	NR
Wan et al. (2014)	Bone metastasis (including lung, breast, prostate, cervical, gastric, other type primary cancers)	RCT	38 (19/19); 53 ± 6.2 years	34 (17/17); 56.3 ± 2.0 years	1) Compound Sangzhi mixture (p.o., b.i.d.) 2) Morphine sulfate sustained-release tablets (30 mg, p.o., b.i.d.)	1) Morphine sulfate sustained-release tablets (30 mg, p.o., b.i.d.)	1) Response rate (*p*-value NR)	30 days	E: 7 cases (3 gastrointestinal reactions including nausea and vomiting, 4 constipation) C: 7 cases (10 gastrointestinal reaction including nausea and vomiting, 7 constipation, 2 urinary retention, 1 central nervous system toxicity)
Song et al. (2015)	Mixed (including liver, abdominal and retroperitoneal lymph node, bone metastasis, lung, pelvis metastatic cancer)	RCT	42 (28/14); range 43–69 years (both groups)	42 (28/14); range 43–69 years (both groups)	1) Total glucoside of paeony capsule (0.6 g, p.o., b.i.d.) 2) Morphine sulfate sustained-release tablets (10 mg, p.o., b.i.d.)	1) Morphine sulfate sustained-release tablets (10 mg, p.o., b.i.d.)	1) Pain intensity (*p* > 0.05)	7 days	E: 4 cases (2 constipation, 1 pruritus, 1 drowsiness) C: 14 cases (4 constipation, 3 pruritus, 1 urinary retention, 5 drowsiness, 1 dyspnea)
Chen et al. (2017)	Bone metastasis (including lung, breast, prostate, ovarian, gastric, renal primary cancers)	RCT	16 (9/7); range 38–77 years	16 (11/5); range 43–78 years	1) Hogu Xioaji prescription (p.o., q.d.) 2) Zoledronic acid with normal saline 250 ml (4 mg, i.v., q.1.w.)	1) Zoledronic acid with normal saline 250 ml (4 mg, i.v., q.1.w.)	1) Response rate (*p* > 0.05) 2) Performance status (*p* > 0.05)	60 days	NR
Li et al. (2017a)	Mixed (including lung, gastric, colon, esophageal, liver, breast cancers)	RCT	90 (50/40); 57.86 ± 16.45 years	90 (48/42); 58.36 ± 15.96 years	1) Xuefu Zhuyu decoction (150 ml, p.o., b.i.d.) 2) Oxycodone hydrochloride sustained-release tablets (10–120 mg, p.o., b.i.d.)	1) Oxycodone hydrochloride sustained-release tablets (10–120 mg, p.o., b.i.d.)	1) Response rate (*p* < 0.05) 2) Pain intensity (*p* < 0.05) 3) Performance status (*p* < 0.05) 4) Opioid usage (*p* < 0.05)	4 week	E: 104 cases (38 constipation, 20 nausea, 18 vomiting, 12 dizziness, 16 anorexia) C: 194 cases (58 constipation, 38 nausea, 30 vomiting, 32 dizziness, 36 anorexia)
Li et al. (2017b)	Mixed (including lung, gastric, colon, esophageal, liver, breast cancers)	RCT	60 (38/22); 51.14 ± 18.42 years	60 (39/21); 50.88 ± 18.42 years	1) Gexia Zhuyu decoction (150 ml, p.o., b.i.d.) 2) Oxycodone hydrochloride sustained-release tablets (10–120 mg, p.o., b.i.d.)	1) Oxycodone hydrochloride sustained-release tablets (10–120 mg, p.o., b.i.d.)	1) Response rate (*p* < 0.05) 2) Pain intensity (*p* < 0.05) 3) Performance status (*p* < 0.05) 4) Duration of pain relief (*p* < 0.05) 5) Opioid usage (*p* < 0.05)	4 weeks	Both groups of patients experienced adverse events such as constipation, nausea, vomiting, dizziness, anorexia, and dysuria. Detailed information NR.
Bao (2018)	Mixed (including lung, gastric, colon, liver, cancers)	RCT	26 (13/13); 57.54 ± 7.11 years	26 (12/14); 56.87 ± 4.54 years	1) Xuefu Zhuyu decoction (200 ml, p.o., b.i.d.) 2) Morphine sulfate controlled-release tablets (10–30 mg, p.o., b.i.d.)	1) Morphine sulfate controlled-release tablets (10–30 mg, p.o., b.i.d.)	1) Response rate (*p* < 0.05)	30 days	E: 6 cases (1 nausea and vomiting, 1 thirst, 1 drowsiness, 3 constipation) C: 13 cases (3 nausea and vomiting, 2 thirst, 1 drowsiness, 7 constipation)
Dong et al. (2018)	Mixed (including lung, gastric, colon, liver, esophageal, breast, prostate cancers)	RCT	120 (65/6); 53.24 ± 16.10 years	120 (67/53); 52.52 ± 16.83 years	1) Cinobufotalin capsules (2 c, p.o., t.i.d.) 2) Morphine sulfate controlled-release tablets (10 mg, p.o., b.i.d.)	1) Morphine sulfate controlled-release tablets (10 mg, p.o., b.i.d.)	1) Response rate (*p* < 0.05) 2) Pain intensity (*p* < 0.01) 3) Performance status (*p* < 0.01) 4) Duration of pain relief (*p* < 0.01) 5) Opioid usage (*p* < 0.01)	30 days	Both groups of patients experienced adverse events such as constipation, nausea, vomiting, dizziness, anorexia, dysuria, etc. Detailed information NR.
Miu and Quan (2018)	Mixed (including lung, gastric, colon, liver, breast cancers)	RCT	23 (13/10); 61.35 ± 9.89 years	23 (14/9); 59.49 ± 10.34 years	1) Cinobufotaling capsules (0.5 g, p.o., t.i.d.) 2) WHO 3-step analgesic ladder treatment: diclofenac sodium sustained-release tablets, profenbeine sustained-release tablets, morphine sulfate sustained-release tablets, etc.	1) WHO 3-step analgesic ladder treatment: diclofenac sodium sustained-release tablets, Profenbeine sustained-release tablets, Morphine sulfate sustained-release tablets, etc.	1) Response rate (*p* < 0.05) 2) Pain intensity (*p* < 0.05) 3) Performance status (*p* < 0.05)	4 weeks	E: 23 cases (9 anorexia, 10 constipation, 4 vomiting) C: 44 cases (16 anorexia, 17 constipation, 11 vomiting)
Ouyang (2018)	Mixed (including gastric, colorectal, liver, breast cancers)	RCT	43 (21/22); 60.04 ± 10.02 years	43 (22/21); 58.76 ± 8.13 years	1) Modified Shaogan fuzi decoction (100 ml, p.o., b.i.d.) 2) Morphine sulfate controlled-release tablets (10–20 mg, p.o., b.i.d.)	1) Morphine sulfate controlled-release tablets (10–20 mg, p.o., b.i.d.)	1) Response rate (*p* < 0.05) 2) Duration of pain relief (*p* < 0.05)	4 weeks	E: 11 cases (9 constipation, 2 nausea and vomiting) C: 32 cases (23 constipation, 9 nausea and vomiting)
Liu (2020)	Rectal cancer	RCT	30 (17/13); 60.6 ± 5.4 years	30 (15/15); 60.5 ± 5.3 years	1) Liuhunzi decoction (150 ml, p.o., b.i.d.) 2) Irinotecan hydrochloride (40 mg, i.v., first treatment) 3) Capecitabine (500 mg, p.o., b.i.d.)	1) Irinotecan hydrochloride (40 mg, i.v., first treatment) 2) Capecitabine (500 mg, p.o., b.i.d.)	1) Response rate (*p* < 0.05) 2) Pain intensity (*p* < 0.05)	6 weeks	NR
Yang (2020)	Mixed (including lung primary cancer)	RCT	35 (17/13); 56.98 ± 3.62 years	35 (25/10) 57.59 ± 3.58 years	1) EAHM formula for individual research (200 ml, p.o., b.i.d.) 2) Zoledronic acid with normal saline 100 ml (40 mg, i.v., first treatment)	1) Zoledronic acid with normal saline 100 ml (40 mg, i.v., first treatment)	1) Response rate (*p* < 0.05) 2) Pain intensity (*p* < 0.05)	2 weeks	E: 4 cases (1 fever, 1 bone joint pain, 2 gastrointestinal reaction) C: 3 cases (1 fever, 1 bone joint pain, 1 gastrointestinal reaction)
Liang et al. (2021)	Mixed (including lung, gastric, liver, colon, breast, cervical cancers)	RCT	39 (23/16); 59.6 ± 7.5 years	39 (22/17); 58.2 ± 7.2 years	1) Cinobufotalin capsules (0.5 g, p.o., t.i.d.) 2) WHO 3-step analgesic ladder treatment: diclofenac sodium sustained-release tablets (1 t, p.o., q.d.); profenbeine sustained-release tablets (2–4 t, p.o., b.i.d.); morphine sulfate sustained-release tablets (1–2 t, p.o., b.i.d.)	1) WHO 3-step analgesic ladder treatment: diclofenac sodium sustained-release tablets (1 t, p.o., q.d.); profenbeine sustained-release tablets (2–4 t, p.o., b.i.d.); morphine sulfate sustained-release tablets (1–2 t, p.o., b.i.d.)	1) Response rate (*p* < 0.01) 2) Pain intensity (*p* < 0.01) 3) Performance status (*p* < 0.01)	4 weeks	E: 34 cases (14 anorexia, 14 constipation, 6 vomiting) C: 67 cases (23 anorexia, 24 constipation, 20 vomiting)

AE, adverse event; b.i.d, bis in die; c: capsule; EAHM, East Asian herbal medicine; d, days; g, gram; i.m., intramuscular; i.v., intravenous; m, months; mg, milligram; mL, milliliter; NR, not reported; p.o, per os; p.r, per rectum; q.d., quaque die; RCT, randomized controlled trial; SD, standard deviation; t, tablet; t.i.d, ter in die; WHO, world health organizations; y, years; ㎍, microgram.

### 3.3 Risk of Bias

The methodological quality of 38 included studies is summarized in Table 2. The risk of bias of studies was assessed using the Rob 2.0 tool (Sterne et al., 2019). In domain 2, bias due to deviations from intended interventions, the risk of bias in all studies was rated high. Although all included studies declare randomization, no study adopted the double-blind method, and this is because the subject and the provider of the intervention can be aware of the assigned intervention. On the other hand, almost all studies did not report on the specific randomization method, and all included studies did not have a registered protocol. Consequently, it was impossible to evaluate compliance with the pre-planned statistical analysis method. Therefore, domain 1 and domain 5 were also evaluated as having some concern of risk of bias in most included studies.

**TABLE 2:** Methodological quality of the included studies according to the risk of bias 2.0.

First author (Year)	D1	D2	D3	D4	D5	Overall
Lin et al. (2001)	Sc	H	L	L	Sc	H
Zhang (2001)	Sc	H	L	L	Sc	H
Li et al. (2002)	Sc	H	L	L	Sc	H
Ma et al. (2003)	Sc	H	L	L	Sc	H
Chen (2004)	Sc	H	L	H	Sc	H
Chen et al. (2005)	Sc	H	L	L	Sc	H
Wu et al. (2005)	Sc	H	H	L	H	H
Cao and Xu (2006)	Sc	H	L	H	Sc	H
Zhang et al. (2006)	Sc	H	L	L	Sc	H
Chen (2009)	Sc	H	L	L	Sc	H
Hao (2009)	Sc	H	L	L	Sc	H
Zhai et al. (2009)	Sc	H	L	L	Sc	H
Zhang (2009)	Sc	H	L	L	Sc	H
Zhang (2009)	Sc	H	L	L	Sc	H
Cai (2010)	Sc	H	L	L	Sc	H
Li et al. (2010)	Sc	H	L	L	Sc	H
Fu (2011)	Sc	H	L	L	Sc	H
Wang et al. (2011)	Sc	H	L	L	Sc	H
Zhou (2011)	Sc	H	L	L	Sc	H
Cheng et al. (2012)	Sc	H	H	L	Sc	H
He (2012)	Sc	H	L	L	Sc	H
Meng (2012)	Sc	H	L	L	Sc	H
Jiang et al. (2013)	Sc	H	L	L	Sc	H
Wang S. J. et al. (2013)	Sc	H	L	H	Sc	H
Chen H. et al. (2014)	Sc	H	L	L	Sc	H
Liu and Zhou (2014)	Sc	H	L	L	H	H
Wan et al. (2014)	Sc	H	L	L	Sc	H
Song et al. (2015)	Sc	H	H	H	Sc	H
Chen et al. (2017)	Sc	H	L	L	Sc	H
Li et al. (2017a)	Sc	H	L	L	Sc	H
Li et al. (2017b)	Sc	H	L	L	Sc	H
Bao (2018)	Sc	H	L	H	Sc	H
Dong et al. (2018)	Sc	H	L	L	Sc	H
Miu and Quan (2018)	Sc	H	L	L	Sc	H
Ouyang (2018)	Sc	H	L	H	Sc	H
Liu (2020)	Sc	H	L	L	Sc	H
Yang (2020)	Sc	H	L	L	Sc	H
Liang et al. (2021)	Sc	H	L	L	Sc	H

D1-D5: 5 domain criteria.

D1: bias arising from the randomization process; D2: bias due to deviations from intended interventions; D3: bias due to missing outcome data; D4: bias in the measurement of the outcome; D5: bias in the selection of the reported results.

H, high risk of bias; L, low risk of bias; Sc, Some concerns.

### 3.4 Primary Outcomes

#### 3.4.1 Response Rate

**FIGURE 2:**
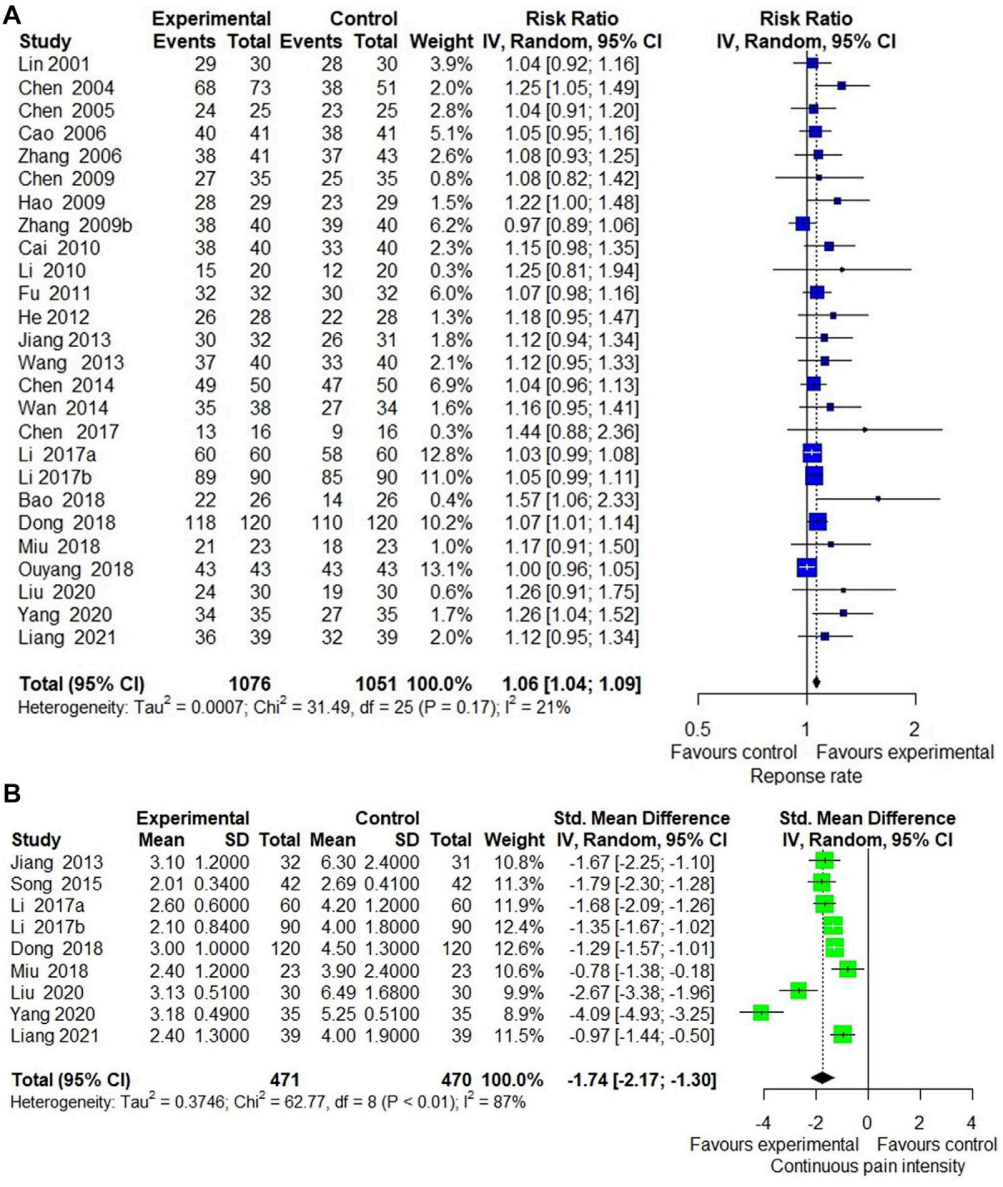
Forest plot of the trials that compared ECCM with CM alone for **(A)** response rate and **(B)** pain intensity.

**FIGURE 3:**
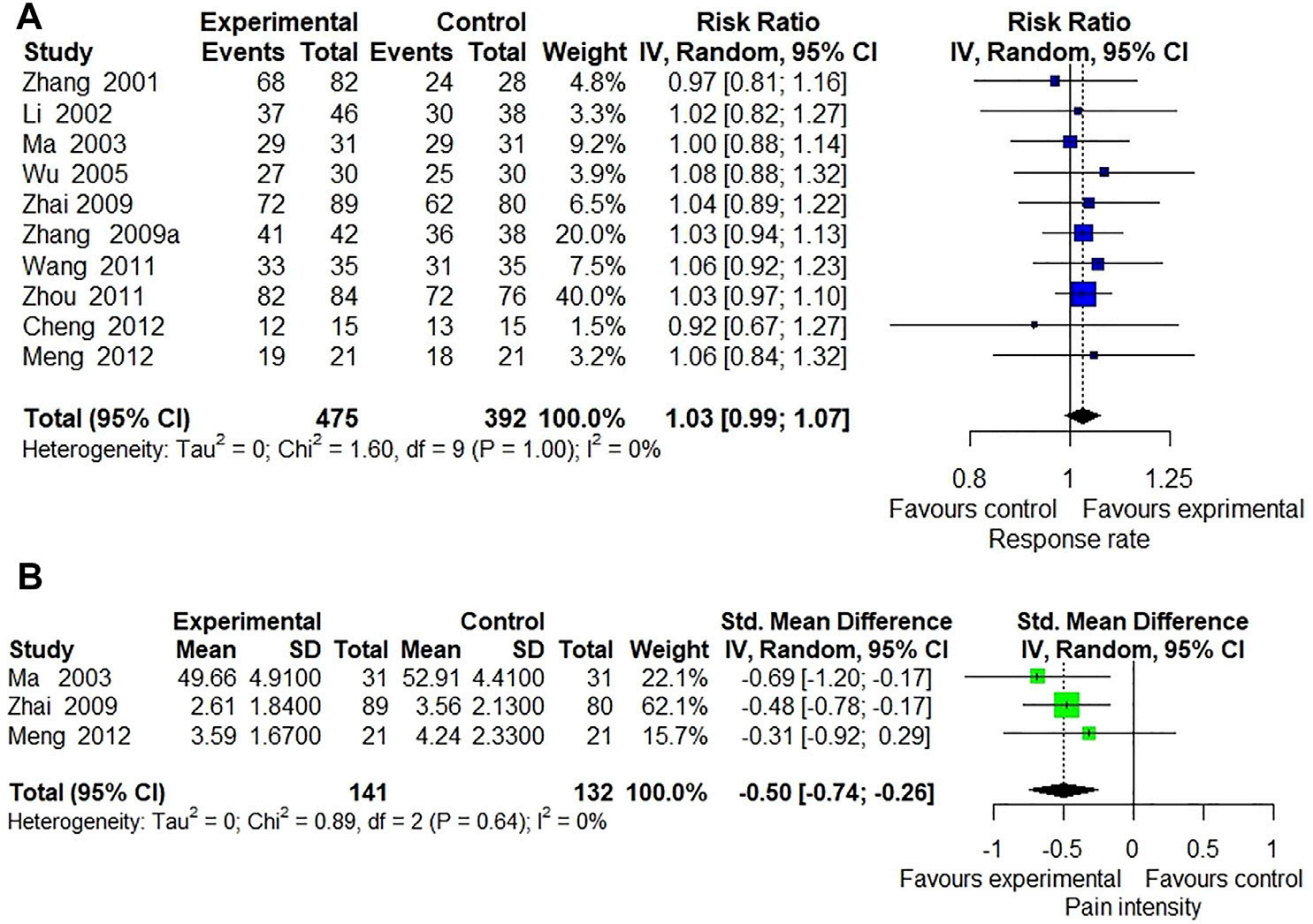
Forest plot of the trials that compared EAHM with CM for **(A)** response rate and **(B)** pain intensity.

**FIGURE 4:**
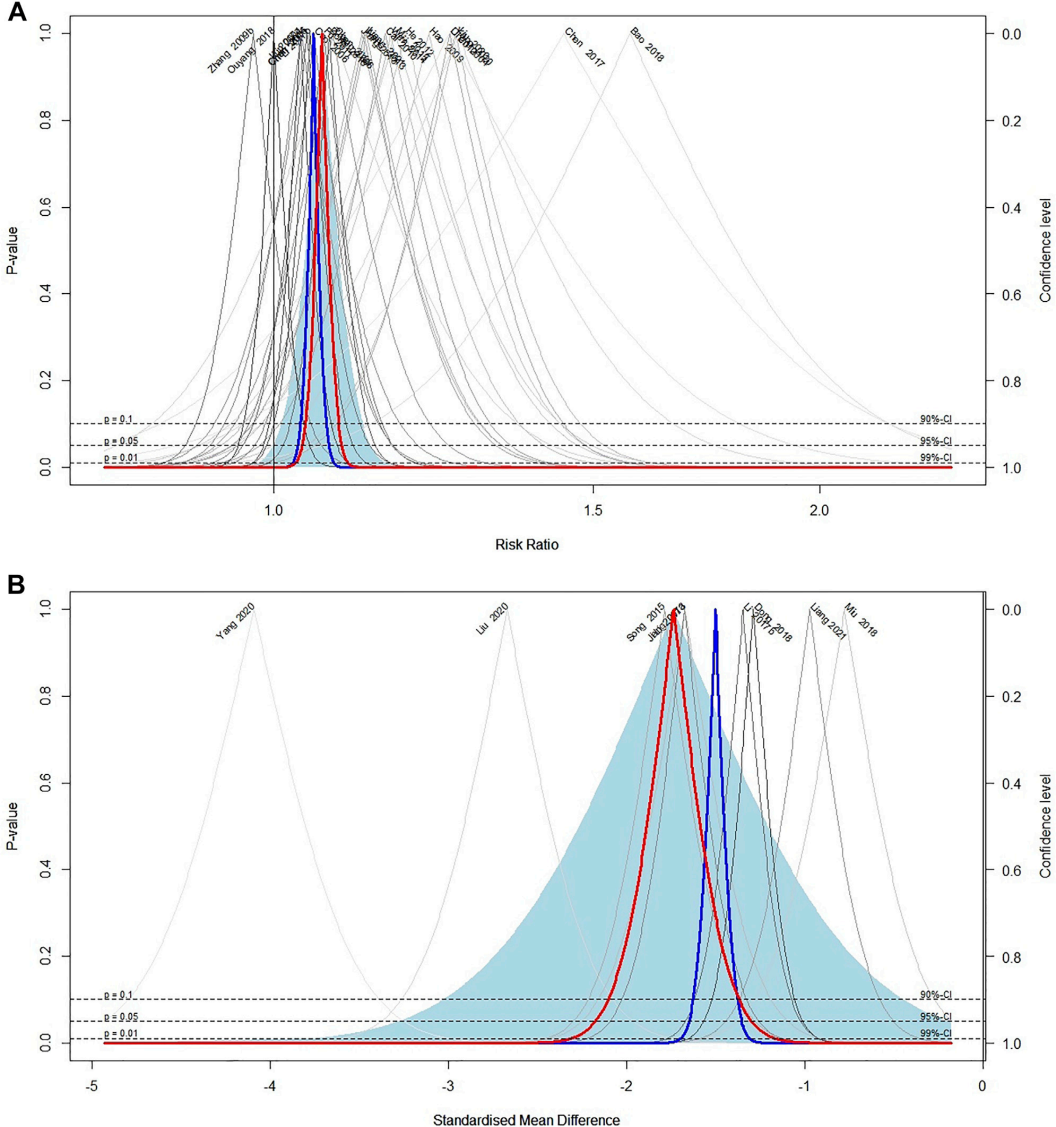
Drapery plot of the trials that compared ECCM with CM alone for **(A)** response rate and **(B)** pain intensity.

**FIGURE 5:**
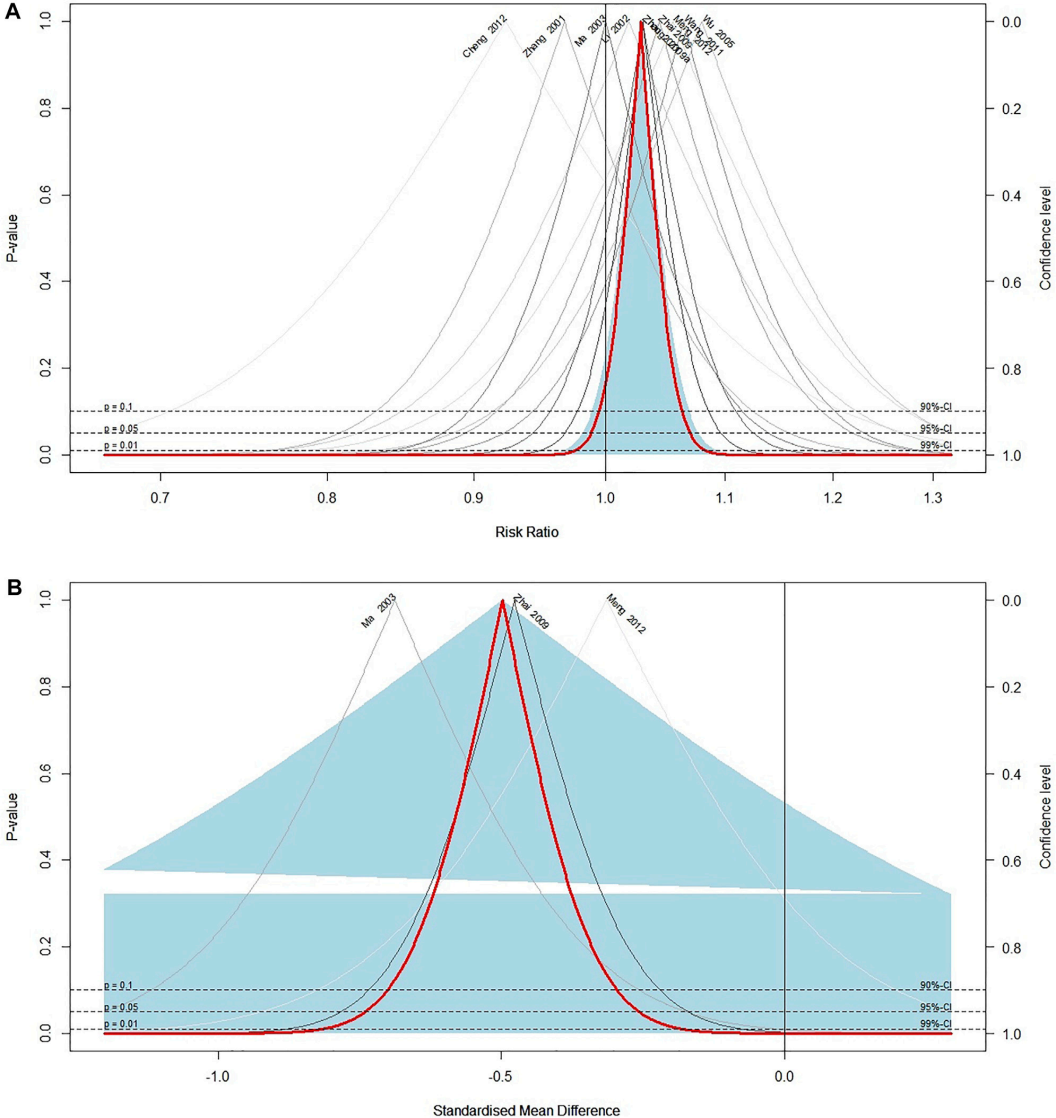
Drapery plot of the trials that compared EAHM with CM for **(A)** response rate and **(B)** pain intensity.

Response rate was reported in 37 included trials. Meta-analysis of 26 trials (Lin et al., 2001; Chen, 2004; Chen et al., 2005; Cao and Xu, 2006; Chen, 2009; Hao, 2009; Zhang et al., 2009; Cai, 2010; Li et al., 2010; Fu, 2011; He, 2012; Jiang et al., 2013; Wang and Chen, 2013; Chen H. et al., 2014; Wan et al., 2014; Chen et al., 2017; Li et al., 2017a; Li et al., 2017b; Bao, 2018; Dong et al., 2018; Miu and Quan, 2018; Ouyang, 2018; Liu, 2020; Yang, 2020; Liang et al., 2021) comparing ECCM with CM revealed a significant effect of ECCM in response rate (26 trials, *n* = 2127; RR: 1.06; 95% CI: 1.04 to 1.09, I^2^ = 21%, *p* < 0.0001; Figure 2A). However, there is no significant difference between EAHM and CM on response rate (10 trials, *n* = 867; RR: 1.03; 95% CI: 0.99 to 1.07, I^2^ = 0%, *p* = 0.1654; Figure 3A). A visual summary of the confidence level for individual studies and pooled estimates using the response rate as the primary outcome was presented through a drapery plot (Figure 4A, Figure 5A).

#### 3.4.2 Continuous Pain Intensity

Continuous pain intensity was measured in 12 included trials. In 9 studies (Jiang et al., 2013; Song et al., 2015; Li et al., 2017a, Li et al., 2017b; Dong et al., 2018; Miu and Quan, 2018; Liu, 2020; Yang, 2020; Liang et al., 2021) comparing ECCM with CM, ECCM was found to be significantly less effective in continuous pain intensity than CM (9 trials, *n* = 941, SMD: −1.74; 95% CI: −2.17 to −1.30; I^2^ = 87%, *p* < 0.0001; Figure 2B). Compared with CM, EAHM exhibited significant improvement on continuous pain intensity (3 trials, *n* = 273, SMD: −0.50; 95% CI: −0.74 to -0.26; I^2^ = 0%, *p* < 0.0001; Figure 5B). A visual summary of the confidence level for individual studies and pooled estimates using the continuous pain intensity as primary outcome was presented through a drapery plot (Figure 4B, Figure 5B).

### 3.5 Secondary Outcomes

#### 3.5.1 Duration of Pain Relief

**FIGURE 6:**
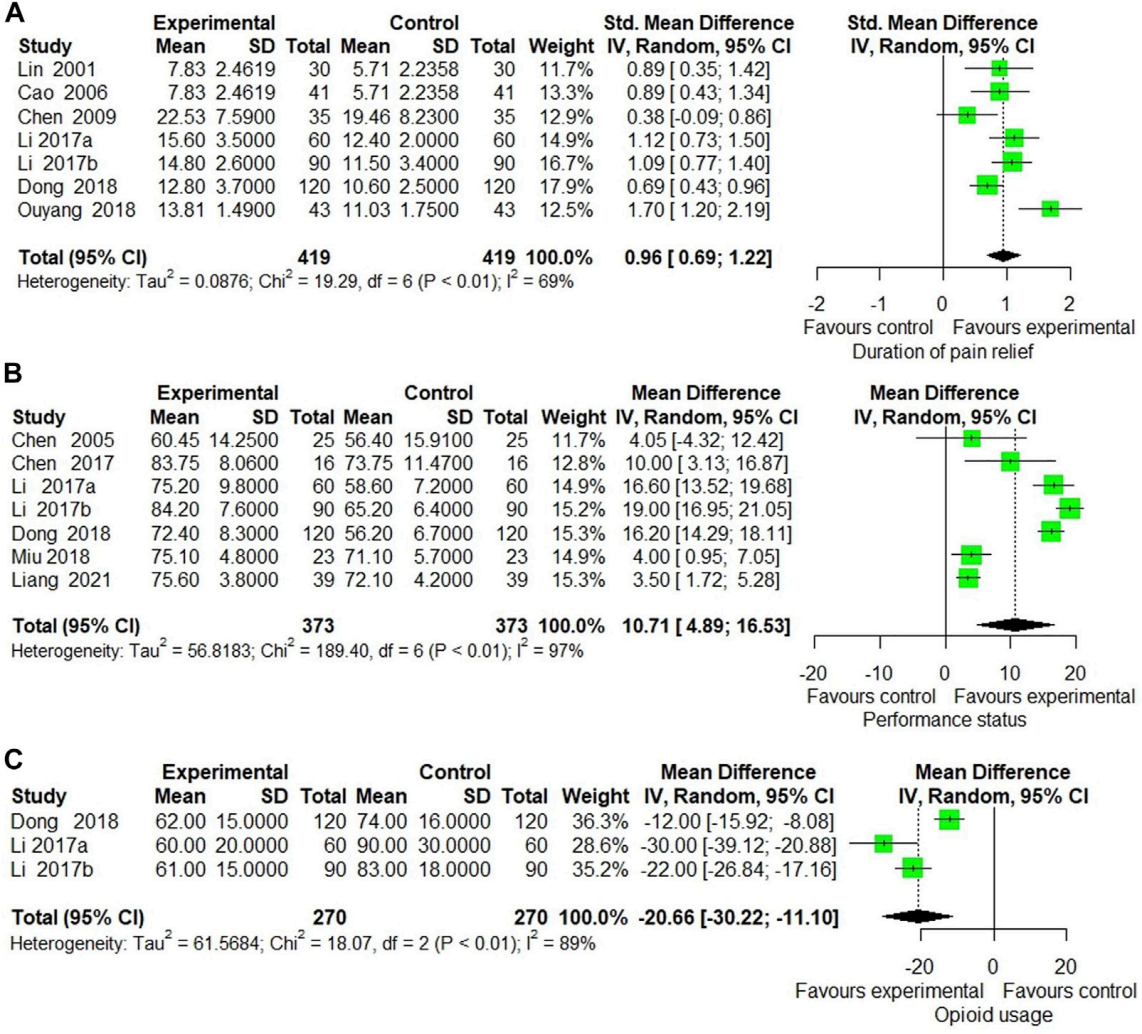
Forest plot of the trials that compared ECCM with CM alone for **(A)** duration of pain relief, **(B)** performance status, and **(C)** opioid usage.

Duration of pain relief was reported in 7 trials (Lin et al., 2001; Cao and Xu, 2006; Chen, 2009; Li et al., 2017a; Li et al., 2017b; Dong et al., 2018; Ouyang, 2018) that compared ECCM with CM. The meta-analysis showed a significant enhancement by ECCM in duration of pain relief (7 trials, *n* = 838, SMD: 0.96, 95% CI: 0.69 to 1.22; I^2^ = 69%, *p* < 0.0001; Figure 6A). However, no significant statistical difference was identified in 1 trial measuring the effect of EAHM on the duration of pain compared to the CM (1 trial, *n* = 55, SMD: −0.09; 95% CI: −0.62 to 0.45; *p* > 0.05) (Wu et al., 2005).

#### 3.5.2 Performance Status

Seven trials measured the effect of ECCM on performance status compared with CM. The meta-analysis revealed a significant improvement in performance status by ECCM (7 trials, *n* = 746, WMD: 10.71; 95% CI: 4.89 to 16.53; I^2^ = 97%, *p* = 0.0003; Figure 6B).

#### 3.5.3 Opioid Usage

Opioid usage was measured in three trials that compared ECCM with CM. The meta-analysis showed a significant reduction by ECCM in opioid usage (3 trials, *n* = 540; WMD: −20.66 mg/day; 95% CI: −30.22 to −11.10; I^2^ = 89%, *p* < 0.0001; Figure 6C).

#### 3.5.4 Adverse Events

In total, 30 trials (30/38, 78.94%) (Lin et al., 2001; Zhang, 2001; Li et al., 2002; Chen et al., 2005; Wu et al., 2005; Cao and Xu, 2006; Zhang et al., 2006; Chen, 2009; Zhai et al., 2009; Zhang, 2009; Zhang et al., 2009; Fu, 2011; Wang et al., 2011; Zhou, 2011; Cheng et al., 2012; He, 2012; Meng, 2012; Wang and Chen, 2013; Chen H. et al., 2014; Wan et al., 2014; Song et al., 2015; Li et al., 2017a; Li et al., 2017b; Bao, 2018; Dong et al., 2018; Miu and Quan, 2018; Ouyang, 2018; Yang, 2020; Liang et al., 2021) reported information on adverse events (AEs). The side effects that occur during the treatment of cancer pain are mainly reported in three areas: upper alimentary tract reactions including nausea and vomiting, lower gastrointestinal tract reactions, such as constipation and diarrhea, and neurologic symptoms such as drowsiness, dizziness, and headache (Scarborough and Smith, 2018). Accordingly, the incidence rates between groups were compared by dividing the findings of AEs reported into the above-mentioned three categories and one category including other symptoms such as burning sensation, fever, fatigue, hypocalcemia, and pruritus in this study. Considering that more than one AE is observed in one patient, if there are several types of AEs observed within an individual symptom category, the type of measurement findings and the number of subjects in each group were multiplied for analysis. Meta-analysis of the upper alimentary tracts’ reaction category showed that the use of EAHM or ECCM significantly reduced the incidences of AEs (20 trials; OR: 0.36; 95% CI: 0.29 to 0.45; *p* < 0.0001; Supplementary Figure S3A). The aggregated results of the lower intestinal tracts reaction category suggested that the incidence of AEs was significantly reduced by ECCM or EAHM (16 trials; OR: 0.32; 95% CI: 0.24 to 0.44; *p* < 0.0001; Supplementary Figure S3B). In addition, the meta-analysis showed that administration of EAHM alone or in combination with CM could reduce the incidence of AEs in the neurologic symptoms category (9 trials; OR: 0.74; 95% CI: 0.28 to 0.74; *p* < 0.0001; Supplementary Figure S3C) and other symptom categories. (12 trials; OR: 0.40; 95% CI: 0.24 to 0.65; *p* < 0.0001; Supplementary Figure S3D). All the reported AEs were not severe and disappeared without long-term treatment. The details of adverse events reported for each study are recorded in Table 1.

### 3.6 Meta-Regression and Subgroup Analysis

As a result of nine trials comparing the effects of continuous pain intensity between ECCM and CM, Higgins I^2^ was 87%, suggesting heterogeneity. Therefore, meta-regression was performed on this result to search for a moderator that induces a potential cause of heterogeneity. As potential moderators, type of cancer, use of opioids in the control group, and duration of treatment were assumed. As a result of meta-regression, there was no statistically significant difference between mixed cancer and single cancer type subgroups (*p* = 0.535), but significant statistical differences were confirmed between subgroups according to whether opioids were adopted in the control group (*p* = 0.003). Moreover, there was a statistically significant difference between the subgroup with a treatment duration of 2 weeks or more and the subgroup with a treatment duration of 2 weeks or less (*p* = 0.034). These results are shown in the bubble plot (Supplementary Figures S4A–C). However, a moderator affecting heterogeneity was not identified in the subgroup analysis, as shown in Table 3. For other outcome measurements, additional subgroup analysis could not be attempted due to the low heterogeneity or the very small number of included studies.

**TABLE 3:** Subgroup analysis of the trials that compared ECCM with CM alone.

	k	Effect size (g)	95% CI	Heterogeneity (I^2^)	P (%)_subgroup_
Type of control group					0.0365
Opioid use	6	−1.3363	−1.4959; −1.1767	90.8	
Other CM	3	−2.5206	−2.9160; −2.1252	55.7	
Duration of treatment					0.2061
>2w	7	−1.3841	−1.5414; −1.2267	73.8	
≤2w	2	−2.4082	−2.8440; −1.9724	95.3	

CM, conventional medicine; ECCM, East Asian herbal medicine combined conventional medicine.

### 3.7 Quality of Evidence According to Outcome Measures

In the comparison between ECCM and CM, the overall quality of evidence according to all outcome measures was low. Meanwhile, in EAHM monotherapy compared with CM, the overall quality of evidence according to all outcome measures was low to moderate. The results of the GRADE assessment are presented in Table 4.

**TABLE 4:** Summary of findings for studies in this meta-analysis.

Intervention and comparator intervention	Outcomes	Number of participants (studies)	Anticipated absolute or relative effects (95%CI)	Quality of the evidence (GRADE)
ECCM compared to CM for cancer pain	Response rate	2127 (26 RCTs)	RR 1.06 more (1.04 more to 1.09 more)	⊕⊕⊕○ MODERATE^a^
	Continuous pain intensity	841 (9 RCTs)	SMD 1.74 SD lower (2.17 lower to 1.3 lower)	⊕⊕○○ LOW^a^ ^,^ ^b^
	Duration of pain relief	838 (7 RCTs)	SMD 0.93 SD higher (0.67 higher to1.2 higher)	⊕⊕○○ LOW^a^ ^,^ ^b^
	Performance status	746 (7 RCTs)	MD 10.71 higher (4.89 higher to 16.53 higher)	⊕⊕○○ LOW^a^ ^,^ ^b^
	Opioid usage	540 (3 RCTs)	MD 20.66 lower (30.22 lower to 11. lower)	⊕⊕○○ LOW^a^ ^,^ ^b^
EAHM monotherapy compared CM for cancer pain	Response rate	867 (10 RCTs)	RR 1.03 (0.99–1.07)	⊕○○○ VERY LOW^a^ ^,^ ^b^ ^,^ ^c^
	Continuous pain intensity	273 (3 RCTs)	SMD 0.5 SD lower (0.74 lower to 0.26 lower)	⊕⊕○○ LOW^a^ ^,^ ^c^
	Duration of pain relief	55 (1 RCT)	SMD 0.18 SD higher (0.18 lower to 0.53 higher)	⊕○○○ VERY LOW^a^ ^,^ ^b^ ^,^ ^c^

EAHM, East Asian herbal medicine; ECCM, East Asian herbal medicine combined conventional medicine; CM, conventional medicine; MD, mean difference; RR, risk ratio; RCT, randomized clinical trial; SD, standardized difference; SMD, standardized mean difference.

GRADE, working group grades of evidence.

High quality: further research is very unlikely to change our confidence in the estimate of effect.

Moderate quality: further research is likely to have an important impact on our confidence in the estimate of effect and may change the estimate.

Low quality: further research is very likely to have an important impact on our confidence in the estimate of effect and is likely to change the estimate.

Very low quality: very uncertain about the estimate.

a Study design with some bias in randomized or distributed blind.

b The confidence intervals are less overlapping, and the heterogeneity is high.

c The 95% confidence interval passes 0 (MD and SMD) or 1 (RR and OR) and the other interventions (OIs) are not satisfied.

### 3.8 Publication Bias

**FIGURE 7:**
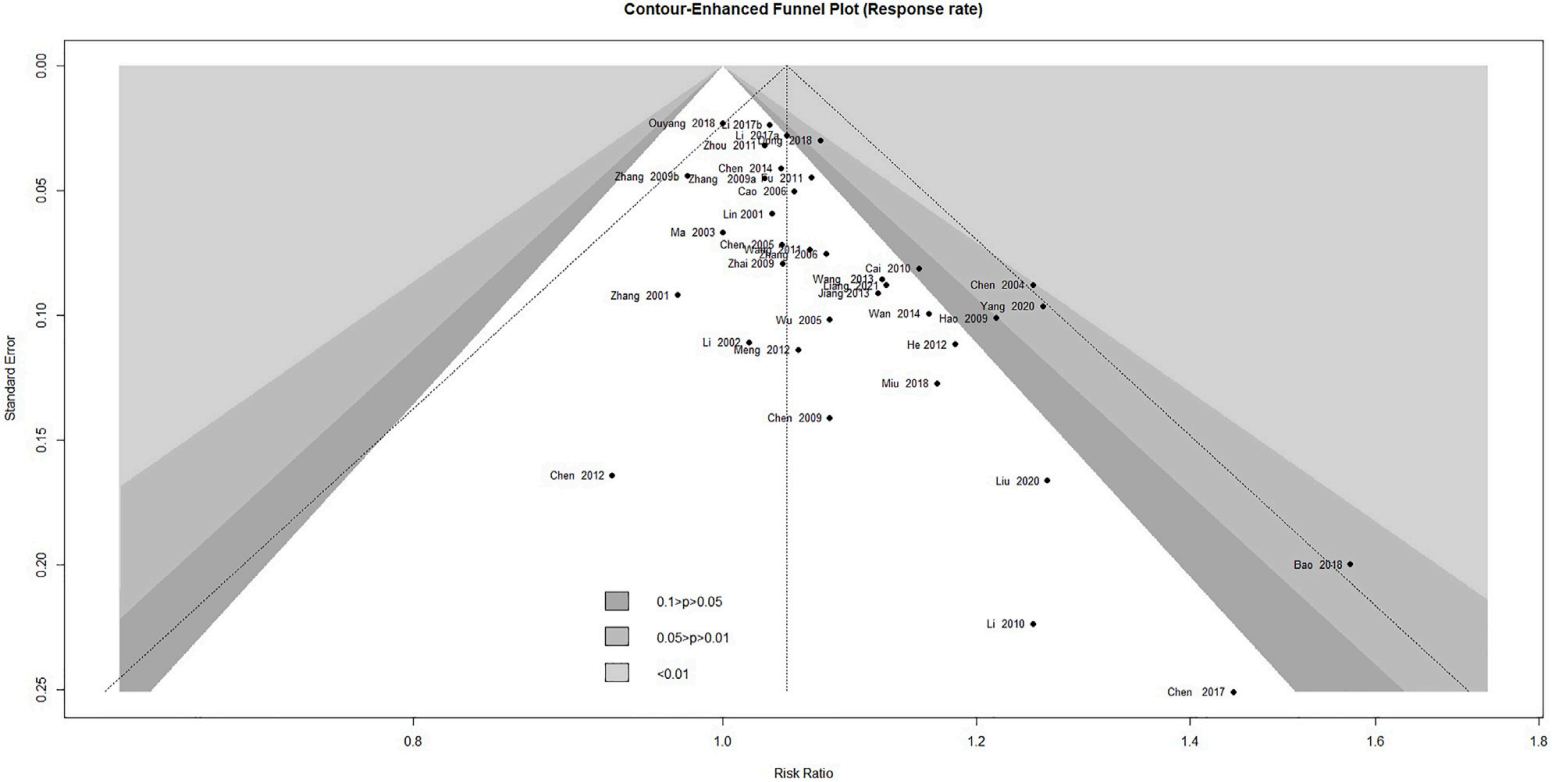
Contour-enhanced funnel plot of the trials for response rate.

Contour-enhanced funnel plot analysis was performed to explore publication bias through the response rate, which is the outcome covering the most included studies. As shown in Figure 7, the pattern in the funnel plot, including 37 studies, showed obvious asymmetry, indicating that there might have been publication bias. This was further confirmed by Egger's test (*p* < 0.0001) and Begg's test (*p* = 0.0013).

### 3.9 Association Rule Mining of EAHM Ingredients

#### 3.9.1 Detailed Information and Distribution of EAHM Ingredients

A total of 125 herbs were used in 38 studies included in this review. Detailed information and types of preparations of herbs constituting EAHM prescriptions are summarized in Table 5. Among them, the top 10 most frequently prescribed herbs for cancer pain were *Glycyrrhiza uralensis* Fisch. ex DC. [Fabaceae], *Paeonia lactiflora* Pall. [Paeoniaceae], *Angelica sinensis* (Oliv.) Diels [Apiaceae], *Prunus persica* (L.) Batsch [Rosaceae], *Corydalis ternata* (Nakai) Nakai [Papaveraceae], *Carthamus tinctorius* L. [Asteraceae], *Pinellia ternata* (Thunb.) Makino [Araceae], *Cullen corylifolium* (L.) Medik. [Fabaceae], *Buthus martensii* Karsch, and *Scrophularia ningpoensis* Hemsl. [Scrophulariaceae]. The relative frequencies of the herb ingredients, which were used in the top 10, ranged from 21.05% to a maximum of 52.63%. The frequency distribution of herbs is shown in Table 6.

**TABLE 5:** The ingredients of EAHM used in the included studies.

Study	EAHM prescription name	Source	Ingredients of EAHM prescription (Latin name)	Ingredients of EAHM prescription (Scientific name)	*Types of preparation*	Quality control reported? (Y/N)	Chemical analysis reported? (Y/N)
Lin et al. (2001)	Jiawei niantong capsule	Prepared by Lin et al. (2001)	Corydalis Tuber, Cyperi Rhizoma, Notoginseng Radix et Rhizoma, Aquilariae Lignum, Curcumae Rhizoma, Citri Unshius Pericarpium, Nardostachyos Radix et Rhizoma, Cinnabaris, Rhei Radix et Rhizoma, Bomeolum	*Corydalis ternata (Nakai) Nakai* [Papaveraceae], *Cyperus rotundus* L. [Cyperaceae], *Panax notoginseng* (Burkill) F.H.Chen [Araliaceae], *Aquilaria malaccensis* Lam. [Thymelaeaceae], *Curcuma phaeocaulis Valeton* [Zingiberaceae], *Citrus deliciosa* Ten. [Rutaceae], *Nardostachys jatamansi* (D.Don) DC. [Caprifoliaceae], *Dracaena cinnabari* Balf.f. [Asparagaceae], *Rheum palmatum* L. [Polygonaceae]*, Dryobalanops aromatica* C.F.Gaertn. [Dipterocarpaceae]	Capsule	N	N
Zhang (2001)	Compound Strynchnos capsule	Prepared by Zhang (2001)	Strychni Semen 0.25 g, Glycyrrhizae Radix et Rhizoma 0.25 g	*Strychnos nux-vomica* L*.* [Loganiaceae] 0.25 g, *Glycyrrhiza inflata* Batalin [Fabaceae] 0.25 g	Capsule	N	N
Li et al. (2002)	Tibetan medicine Duyiwei	Prepared by Li et al. (2002)	Lamiophlomis rotata	*Phlomoides rotata* (Benth. ex Hook.f.) Mathiesen [Lamiaceae]	Capsule	N	N
Ma et al. (2003)	Jiaweibaoankeli	Prepared by Ma et al. (2003)	Rhei Radix et Rhizoma, Aconiti Lateralis Radix Preparata, Glycyrrhizae Radix et Rhizoma, Arisaematis Rhizoma, Paeoniae Radix	*Rheum palmatum* L*.* [Polygonaceae], *Aconitum carmichaelii* Debeaux [Ranunculaceae], *Glycyrrhiza uralensis* Fisch. ex DC. [Fabaceae], *Arisaema erubescens* (Wall.) Schott [Araceae], *Paeonia lactiflora* Pall. [Paeoniaceae],	Granule	N	N
Chen (2004)	Shitong decoction	Prepared by Chen (2004)	Bupleuri Radix 12 g, Ponciri Fructus Immaturus 12 g, Magnoliae Cortex 12 g, Rhei Radix et Rhizoma 12 g, Salviae Miltiorrhizae Radix 30 g, Paeoniae Radix 20 g, Typhae Pollen 12 g, Curcumae Rhizoma 30 g, Notoginseng Radix et Rhizoma 12 g, Corydalis Tuber 12 g, Coptidis Rhizoma 6 g, Hedyotidis Herba 30 g, Scutellariae Barbatae Herba 25 g	*Bupleurum falcatum* L. [Apiaceae] 12 g, *Citrus trifoliata* L. [Rutaceae] 12 g, *Magnolia officinalis* Rehder & E.H.Wilson [Magnoliaceae] 12 g, *Rheum palmatum* L. [Polygonaceae] 12 g, *Salvia miltiorrhiza* Bunge [Lamiaceae] 30 g, *Paeonia lactiflora* Pall. [Paeoniaceae] 20 g, *Typha orientalis* C.Presl [Typhaceae] 12 g, *Curcuma phaeocaulis* Valeton [Zingiberaceae] 30 g, *Panax notoginseng* (Burkill) F.H.Chen [Araliaceae] 12 g, *Corydalis ternata* (Nakai) Nakai [Papaveraceae] 12 g, *Coptis chinensis* Franch. [Ranunculaceae] 6 g, *Scleromitrion diffusum* (Willd.) R.J.Wang [Rubiaceae] 30 g, *Scutellaria barbata* D.Don [Lamiaceae] 25 g	Decoction	N	N
Chen et al. (2005)	Zhitong capsule	Prepared by Chen et al. (2005)	Asiasari Radix et Rhizoma, Paeoniae Radix, Cnidii Rhizoma, Cynanchi Paniculati Radix Et Rhizoma	*Asarum sieboldii* Miq. [Aristolochiaceae], *Paeonia lactiflora* Pall. [Paeoniaceae], *Conioselinum anthriscoides “Chuanxiong”* [Apiaceae], *Vincetoxicum mukdenense* Kitag. [Apocynaceae]	Capsule	N	N
Wu et al. (2005)	Aitongping capsule	Prepared by Wu et al. (2005)	Paridis Rhizoma, Oniscus, Arisaematis Rhizoma, Aconiti Koreani Tuber, Olibanum, Piperis Longi Fructus, Corydalis Tuber	*Paris polyphylla var. chinensis* (Franch.) H.Hara [Melanthiaceae], *Armadillidium vulgare* Latreille, *Arisaema erubescens* (Wall.) Schott [Araceae], *Aconitum coreanum (H.Lév.) Rapaics* [Ranunculaceae], *Boswellia carteri* Birdw*.* [Burseraceae], *Piper longum* L. [Piperaceae], *Corydalis ternata* (Nakai) Nakai [Papaveraceae]	Capsule	N	N
Cao and Xu (2006)	Zhunaggu Zhitoing Powder	Prepared by Cao and Xu (2006)	Angelicae Sinensis Radix 12 g, Rehmanniae Radix Preparata 15 g, Loranthi Ramulus Et Folium 15 g, Manis pentadactyla 15 g, Psoraleae Semen 15 g, Drynariae Rhizoma 15 g, Paeoniae Radix 15 g, Corydalis Tuber 15 g, Notoginseng Radix et Rhizoma 6 g, Curcumae Rhizoma 10 g, Arisaematis Rhizoma 10 g, Scolopendra 2 pieces, Lumbricus 15 g, Scorpio 5 g, Citri Unshius Pericarpium 10 g	*Angelica sinensis* (Oliv.) Diels [Apiaceae] 12 g, *Rehmannia glutinosa (*Gaertn.) DC. [Orobanchaceae] 15 g, *Taxillus chinensis* (DC.) Danser [Loranthaceae] 15 g, *Manis pentadactyla* Linné 15 g, *Cullen* corylifolium (L.) Medik. [Fabaceae] 15 g, *Drynaria roosii* Nakaike [Polypodiaceae] 15 g, *Paeonia lactiflora* Pall. [Paeoniaceae] 15 g, *Corydalis ternata* (Nakai) Nakai [Papaveraceae] 15 g, *Panax notoginseng* (Burkill) F.H.Chen [Araliaceae] 6 g, *Curcuma phaeocaulis* Valeton [Zingiberaceae] 10 g, *Arisaema erubescens (Wall.) Schott* [Araceae] 10 g, *Scolopendra subspinipes mutilans* Linné Koch 2 pieces, *Amynthas pectiniferus* 15 g, *Buthus martensii* Karsch 5 g, *Citrus deliciosa* Ten. [Rutaceae] 10 g	Decoction	N	N
Zhang et al. (2006)	EAHM formula for individual research	Prepared by Zhang et al. (2006)	Asparagi Tuber 9 g, Liriopis seu Ophiopogonis Tuber 9 g, Scrophulariae Radix 9 g, Rehmanniae Radix Recens 9 g, Bupleuri Radix 10 g, Aurantii Fructus Immaturus 10 g, Corydalis Tuber 10 g, Cyperi Rhizoma 10 g, Paeoniae Radix 12 g, Persicae Semen Sinensis Radix 12 g, Notoginseng Radix et Rhizoma 12 g, Citri Unshius Pericarpium Immaturus 6 g, Persicae Semen 6 g, Glycyrrhizae Radix et Rhizoma 3 g	*Asparagus cochinchinensis* (Lour.) Merr. [Asparagaceae] 9 g, *Liriope muscari* (Decne.) L.H.Bailey [Asparagaceae] 9 g, *Scrophularia ningpoensis* Hemsl. [Scrophulariaceae] 9 g, *Rehmannia glutinosa* (Gaertn.) DC. [Orobanchaceae] 9 g, *Bupleurum falcatum* L.[Apiaceae] 10 g, *Citrus × aurantium* L. [Rutaceae] 10 g, *Corydalis ternata* (Nakai) Nakai [Papaveraceae] 10 g, *Cyperus rotundus* L. [Cyperaceae] 10 g, *Paeonia lactiflora* Pall. [Paeoniaceae] 12 g, *Angelica sinensis* (Oliv.) Diels [Apiaceae] 12 g, *Panax notoginseng* (Burkill) F.H.Chen [Araliaceae] 12 g, *Citrus deliciosa* Ten. [Rutaceae] 6 g, *Prunus persica* (L.) Batsch [Rosaceae] 6 g, *Glycyrrhiza uralensis* Fisch. ex DC. [Fabaceae] 3 g	Decoction	N	N
Chen (2009)	Jiawei Shentong Zhuyu decoction	Prepared by Chen (2009)	Gentianae Macrophyllae Radix 12 g, Cnidii Rhizoma 12 g, Persicae Semen 12 g, Carthami Flos 9 g, Glycyrrhizae Radix et Rhizoma 6 g, Osterici seu Notopterygii Radix et Rhizoma 9 g, Myrrha 9 g, Angelicae Sinensis Radix 15 g, Trogopterorum Faeces 9 g, Cyperi Rhizoma 9 g, Achyranthis Radix 15 g, Lumbricus 9 g, Scolopendra 2 pieces, Scorpio 10 g, Psoraleae Semen 15 g, Drynariae Rhizoma 15 g, Herba Speranskiae Tuberculatae 15 g, Loranthi Ramulus Et Folium 15 g	*Gentiana macrophylla* Pall. [Gentianaceae] 12 g, *Conioselinum anthriscoides 'Chuanxiong'* [Apiaceae] 12 g, *Prunus persica* (L.) Batsch [Rosaceae] 12g, *Carthamus tinctorius* L. [Asteraceae] 9g, *Glycyrrhiza uralensis* Fisch. ex DC. [Fabaceae] 6 g, *Ostericum grosseserratum* (Maxim.) Kitag*.* [Apiaceae] 9 g, *Commiphora myrrha* (T.Nees) Engl. [Burseraceae] 9 g, *Angelica sinensis* (Oliv.) Diels 15 g, *Trogopterus xanthipes* 9 g, *Cyperus rotundus* L. [Cyperaceae] 9 g, *Achyranthes bidentata* Blume [Amaranthaceae] 15 g, *Amynthas pectiniferus* 9 g, *Scolopendra subspinipes mutilans *Linné Koch 2 pieces, *Buthus martensii* Karsch 10 g, *Cullen corylifolium* (L.) Medik. [Fabaceae] 15 g, *Drynaria roosii* Nakaike [Polypodiaceae] 15 g, *Speranskia tuberculata* (Bunge) Baill. [Euphorbiaceae] 15 g, *Taxillus chinensis* (DC.) Danser [Loranthaceae] 15 g	Decoction	N	N
Zhai et al. (2009)	Anti-cancer Zhitong decoction	Prepared by Zhai et al. (2009)	Gecko 15 g, Paridis Rhizoma 15 g, Hedyotidis Herba 30 g, Notoginseng Radix et Rhizoma 6 g, Scorpio 3 g, Scolopendra 2 pieces, Corydalis Tuber 15 g, *Manis pentadactyla* 10 g, Citri Sarcodactylis Fructus 15 g, Aspongopus 10 g, Zizyphi Semen 15g, Cynanchi Paniculati Radix Et Rhizoma 15 g, Succinum 3 g, Moschus 0.1 g	*Gekko chinensis* 15 g, *Paris polyphylla var. chinensis* (Franch.) H.Hara [Melanthiaceae] 15 g, *Scleromitrion diffusum* (Willd.) R.J.Wang [Rubiaceae] 30 g, *Panax notoginseng* (Burkill) F.H.Chen [Araliaceae] 6 g, *Buthus martensii *Karsch 3 g, *Scolopendra subspinipes mutilans *Linné Koch 2 pieces, *Corydalis ternata* (Nakai) Nakai [Papaveraceae] 15 g, *Manidae* 10 g, *Citrus medica* L. [Rutaceae]15 g, *Coridius chinensis* 10 g, *Ziziphus jujuba* Mill*.* [Rhamnaceae] 15 g, *Vincetoxicum mukdenense* Kitag. [Apocynaceae] 15 g, *Pinus densiflora* Siebold & Zucc. [Pinaceae] 3 g, *Moschus moschiferus* Linné 0.1 g	Decoction	N	N
Hao (2009)	EAHM formula for individual research	Prepared by Hao (2009)	Astragali Radix 30 g, Cistanchis Herba 20 g, Eucommiae Cortex 15 g, Dipsaci Radix 15 g, Drynariae Rhizoma 20 g, Psoraleae Semen 15 g, Cibotii Rhizoma 10 g, Achyranthis Radix 10 g, Cynanchi Paniculati Radix Et Rhizoma 20 g, Oniscus 20 g, Lumbricus 10 g, Scorpio 6 g, Corydalis Tuber 15 g, Pinelliae Tuber 10 g, Arisaematis Rhizoma 10 g, Hedyotidis Herba 20 g, Linderae Radix 20 g, Glycyrrhizae Radix et Rhizoma 10 g	*Astragalus mongholicus* Bunge [Fabaceae] 30 g, *Cistanche deserticola* Ma [Orobanchaceae] 20 g, *Eucommia ulmoides* Oliv. [Eucommiaceae] 15 g, *Dipsacus asper* Wall. ex DC. [Caprifoliaceae] 15 g, *Drynaria roosii* Nakaike [Polypodiaceae] 20 g, *Cullen corylifolium* (L.) Medik. [Fabaceae] 15 g, *Cibotium barometz* (L.) J.Sm. [Cyatheaceae] 10 g, *Achyranthes bidentata* Blume [Amaranthaceae] 10 g, *Vincetoxicum mukdenense Kitag.* [Apocynaceae] 20 g, *Armadillidium vulgare* Latreille 20 g, *Amynthas pectiniferus* 10 g, *Buthus martensii* Karsch 6 g, *Corydalis ternata* (Nakai) *Nakai* [Papaveraceae] 15 g, *Pinellia ternata* (Thunb.) *Makino* [Araceae] 10 g, *Arisaema erubescens* (Wall.) Schott [Araceae] 10 g, *Scleromitrion diffusum* (Willd.) R.J.Wang [Rubiaceae] 20 g, *Lindera aggregata* (Sims) Kosterm. [Lauraceae] 20 g, *Glycyrrhiza uralensis* Fisch. ex DC. [Fabaceae] 10 g,	Decoction	N	N
Zhang (2009)	Tuqi powder	Prepared by Zhang (2009a)	Curcumae Longae Rhizoma 30 g, Scrophulariae Radix 30 g, Cassiae Cortex Interior 30 g, Angelicae Sinensis Radix 30 g, Caulis Sargentodoxae 30 g, Scolopendra 30g, Curcumae Radi 30 g, Bupleuri Radix 30 g, Salviae Miltiorrhizae Radix 30 g, Pinelliae Tuber 18 g, Arisaematis Rhizoma 18 g, Rhei Radix et Rhizoma 18 g, Paeoniae Radix 18 g, Glycyrrhizae Radix et Rhizoma 18 g, Ginseng Radix 6 g, Zingiberis Rhizoma Recens 6 g, Atractylodis Rhizoma Alba 9 g, Persicae Semen 9 g, Poria Sclerotium 9 g, Zizyphi Fructus 9 pieces	*Curcuma longa* L. [Zingiberaceae] 30 g, *Scrophularia ningpoensis* Hemsl. [Scrophulariaceae] 30 g, *Neolitsea cassia* (L.) Kosterm*.* [Lauraceae] 30 g, *Angelica sinensis* (Oliv.) Diels [Apiaceae] 30 g, *Sargentodoxa cuneata* (Oliv.) Rehder & E.H.Wilson [Lardizabalaceae] 30 g, *Scolopendra subspinipes mutilans* Linné Koch 30 g, *Curcuma aromatica* Salisb. [Zingiberaceae] 30 g, *Bupleurum falcatum* L. [Apiaceae] 30 g, *Salvia miltiorrhiza* Bunge [Lamiaceae] 30 g, *Pinellia ternata* (Thunb.) Makino *[Araceae]* 18 g, *Arisaema erubescens (*Wall.) Schott [Araceae] 18 g, *Rheum palmatum* L. [Polygonaceae] 18 g, *Paeonia lactiflora* Pall. [Paeoniaceae] 18 g, *Glycyrrhiza uralensis* Fisch. ex DC. [Fabaceae] 18 g, *Panax ginseng* C.A.Mey. [Araliaceae] 6 g, *Zingiber officinale* Roscoe [Zingiberaceae] 6 g, *Atractylodes macrocephala* Koidz*.* [Asteraceae] 9 g, *Prunus persica* (L.) Batsch [Rosaceae] 9 g, *Poria cocos* Wolf 9 g, *Ziziphus jujuba* Mill*.* [Rhamnaceae] 9 pieces	Decoction	N	N
Zhang et al. (2009)	Wendan decoction	Prepared by Zhang et al. (2009)	Phyllostachyos Caulis in Taeniam 6 g, Pinelliae Tuber 6 g, Ponciri Fructus Immaturus 6 g, Citri Unshius Pericarpium 9 g, Glycyrrhizae Radix et Rhizoma 3 g, Poria Sclerotium 4.5 g, Zingiberis Rhizoma Recens 5 pieces, Zizyphi Fructus 1 pieces	*Phyllostachys nigra var. henonis* (Mitford) Rendle [Poaceae] 6 g, *Pinellia ternate* (Thunb.) Makino [Araceae] 6 g, *Citrus trifoliata* L. [Rutaceae] 6 g, *Citrus deliciosa* Ten. [Rutaceae] 9 g, *Glycyrrhiza uralensis* Fisch. ex DC. [Fabaceae] 3 g, *Poria cocos* Wolf 4.5 g, *Zingiber officinale* Roscoe [Zingiberaceae] 5 pieces, *Ziziphus jujuba* Mill. [Rhamnaceae] 1 piece	Decoction	N	N
Cai (2010)	Yanghe decoction	Prepared by Cai (2010)	Rehmanniae Radix Preparata 30 g, Cinnamomi Cortex 9 g, Ephedrae Herba 9 g, Cervi Cornus Colla 10 g, Zingiberis Rhizoma 9 g, Sinapis Semen 10 g, Glycyrrhizae Radix et Rhizoma 6 g, Psoraleae Semen 20 g, Drynariae Rhizoma 15 g, Scorpio 9 g, Scolopendra 2 pieces, Asiasari Radix et Rhizoma 6 g	*Rehmannia glutinosa* (Gaertn.) DC. [Orobanchaceae] 30 g, *Neolitsea cassia* (L.) Kosterm. [Lauraceae] 9 g, *Ephedra sinica* Stapf [Ephedraceae] 9 g, *Cervidae* 10 g, *Zingiber officinale* Roscoe [Zingiberaceae] 9 g, *Sinapis alba* L. [Brassicaceae] 10 g, *Glycyrrhiza uralensis* Fisch. ex DC. [Fabaceae] 6 g, *Cullen corylifolium* (L.) Medik. [Fabaceae] 20 g, *Drynaria roosii* Nakaike [Polypodiaceae] 15 g, *Buthus martensii *Karsch 9 g, *Scolopendra subspinipes mutilans* Linné Koch 2 pieces, *Asarum sieboldii* Miq. [Aristolochiaceae] 6 g	Decoction	N	N
Li et al. (2010)	Taohongsiwu decoction	Prepared by Li et al. (2010)	Persicae Semen 20 g, Carthami Flos 10 g, Angelicae Sinensis Radix 20 g, Rehmanniae Radix Recens 20 g, Paeoniae Radix 20 g, Cnidii Rhizoma 10 g	*Prunus persica* (L.) Batsch [Rosaceae] 20 g, *Pinellia ternata* (Thunb.) Makino [Araceae] 10 g, *Angelica sinensis* (Oliv.) Diels [Apiaceae] 20 g, *Rehmannia glutinosa (*Gaertn.) DC*.* [Orobanchaceae] 20 g, *Paeonia lactiflora* Pall. [Paeoniaceae] 20 g, *Conioselinum anthriscoides 'Chuanxiong'* [Apiaceae]. 10 g	Decoction	N	N
Fu (2011)	Qigetongbu decoction	Prepared by Fu (2011)	Astragali Radix 30 g, Poria Sclerotium 30 g, Coicis Semen 30 g, Amomi Fructus 5 g, Amomi Fructus Rotundus 5 g, Pinelliae Tuber 10 g, Aucklandiae Radix 10 g, Phyllostachyos Caulis in Taeniam 10 g, Caulis Perillae 10 g	*Astragalus mongholicus* Bunge [Fabaceae] 30 g, *Poria cocos* Wolf 30 g, *Coix lacryma-jobi var. ma-yuen* (Rom.Caill.) Stapf [Poaceae] 30 g, *Wurfbainia villosa* (Lour.) Skornick. & A.D.Poulsen [Zingiberaceae] 5 g, *Wurfbainia vera* (Blackw.) Skornick. & A.D.Poulsen [Zingiberaceae]5 g, *Pinellia ternata* (Thunb.) Makino [Araceae] 10 g, *Aucklandia costus* Falc. [Asteraceae]10 g, *Phyllostachys nigra var. henonis* (Mitford) Rendle [Poaceae] 10 g, *Perilla frutescens* (L.) Britton [Lamiaceae]10 g	Decoction	N	N
Wang et al. (2011)	EAHM formula for individual research	Prepared by Wang et al. (2011)	Achyranthis Radix 15 g, Rehmanniae Radix Preparata 30 g, Eucommiae Cortex 15 g, Dipsaci Radix 15 g, Loranthi Ramulus Et Folium 15 g, Paeoniae Radix 40 g, Curcumae Longae Rhizoma 15 g, Notoginseng Radix et Rhizoma 6 g, Sinapis Semen 15 g, Ostreae Testa 30 g, Fritillariae Cirrhosae Bulbus 15 g, Gleditsiae Spina 15 g, Lysimachiae Herba 10 g	*Achyranthes bidentata* Blume [Amaranthaceae] 15 g, *Rehmannia glutinosa* (Gaertn.) DC. [Orobanchaceae] 30 g, *Eucommia ulmoides* Oliv. [Eucommiaceae] 15 g, *Dipsacus asper* Wall. ex DC. [Caprifoliaceae] 15 g, *Taxillus chin*ensis (DC.) Danser [Loranthaceae] 15 g, *Paeonia lactiflora* Pall. [Paeoniaceae] 40 g, *Curcuma longa* L. [Zingiberaceae] 15 g, *Panax notoginseng* (Burkill) F.H.Chen [Araliaceae] 6 g, *Sinapis alba* L. [Brassicaceae]15 g, *Ostrea gigas* Thunberg 30 g, *Fritillaria cirrhosa* D.Don [Liliaceae] 15 g, *Gleditsia sinensis* Lam. [Fabaceae] 15 g, *Lysimachia christinae* Hance [Primulaceae] 10 g	Decoction	N	N
Zhou (2011)	Tuqi powder	Prepared by Zhou (2011)	Scolopendra 30 g, Curcumae Radi 30 g, Bupleuri Radix 30 g, Salviae Miltiorrhizae Radix 30 g, Curcumae Longae Rhizoma 30 g, Scrophulariae Radix 30 g, Cassiae Cortex Interior 30 g, Caulis Sargentodoxae 30 g, Magnoliae Cortex 30 g, Rhei Radix et Rhizoma 18 g, Paeoniae Radix 18 g, Pinelliae Tuber 18 g, Arisaematis Rhizoma 18 g, Glycyrrhizae Radix et Rhizoma 18 g	*Scolopendra subspinipes mutilans* Linné Koch 30 g, *Curcuma aromatica Salisb.* [Zingiberaceae] 30 g, *Bupleurum falcatum* L. [Apiaceae] 30 g, *Salvia miltiorrhiza* Bunge [Lamiaceae] 30 g, *Curcuma longa* L. [Zingiberaceae] 30 g, *Scrophularia ningpoensis* Hemsl. [Scrophulariaceae] 30 g, *Neolitsea cassia* (L.) Kosterm. [Lauraceae] 30 g, *Sargentodoxa cuneata* (Oliv.) Rehder & E.H.Wilson [Lardizabalaceae] 30 g, *Magnolia officinalis* Rehder & E.H.Wilson [Magnoliaceae] 30 g, *Rheum palmatum* L. [Polygonaceae] 18 g, *Paeonia lactiflora* Pall. [Paeoniaceae] 18 g, *Pinellia ternata* (Thunb.) Makino [Araceae] 18 g, *Arisaema consanguineum* Schott [Araceae] 18 g, *Glycyrrhiza uralensis Fisch. ex DC.* [Fabaceae] 18 g	Powder	N	N
Cheng et al. (2012)	Baizhu Fuzi decoction	Prepared by Cheng et al. (2012)	Aconiti Lateralis Radix Preparata 10 g, Atractylodis Rhizoma Alba 6 g, Zingiberis Rhizoma Recens 4.5 g, Zizyphi Fructus 6 pieces, Glycyrrhizae Radix et Rhizoma 3 g	*Aconitum carmichaelii* Debeaux [Ranunculaceae] 10 g, *Atractylodes macrocephala* Koidz. [Asteraceae] 6 g, *Zingiber officinale* Roscoe [Zingiberaceae] 4.5 g, *Ziziphus jujuba* Mill. [Rhamnaceae] 6 pieces, *Glycyrrhiza uralensis* Fisch. ex DC. [Fabaceae] 3 g	Decoction	N	N
He (2012)	EAHM formula for individual research	Prepared by He (2012)	Astragali Radix 30 g, Rehmanniae Radix Preparata 20 g, Psoraleae Semen 10 g, Herba Speranskiae Tuberculatae 15 g, Persicae Semen 10 g, Glycyrrhizae Radix et Rhizoma 9 g, Drynariae Rhizoma 15 g, Spatholobi Caulis 15 g, Achyranthis Radix 12 g, Eupolyphaga 10 g, Corydalis Tuber 12 g, Hedyotidis Herba 25 g, Coicis Semen 20 g, Epimedii Herba 10 g, Paeoniae Radix 15 g, Eucommiae Cortex 12 g	*Astragalus mongholicus* Bunge [Fabaceae] 30 g, *Rehmannia glutinosa* (Gaertn.) DC. [Orobanchaceae] 20 g, *Cullen corylifolium* (L.) Medik. [Fabaceae] 10 g, *Speranskia tuberculata* (Bunge) Baill. [Euphorbiaceae] 15 g, *Prunus persica* (L.) Batsch [Rosaceae] 10 g, *Glycyrrhiza uralensis* Fisch. ex DC. [Fabaceae] 9 g, *Drynaria roosii* Nakaike [Polypodiaceae] 15 g, *Spatholobus suberectus* Dunn [Fabaceae] 15 g, *Achyranthes bidentata* Blume [Amaranthaceae] 12 g, *Eupolyphaga sinensis* Walker 10 g, *Corydalis ternata* (Nakai) Nakai [Papaveraceae] 12 g, *Scleromitrion diffusum* (Willd.) R.J.Wang [Rubiaceae] 25 g, *Coix lacryma-jobi var. ma-yuen* (Rom.Caill.) Stapf [Poaceae] 20 g, *Epimedium brevicornu* Maxim*.* [Berberidaceae] 10 g, *Paeonia lactiflora* Pall. [Paeoniaceae] 15 g, *Eucommia ulmoides* Oliv. [Eucommiaceae] 12 g	Decoction	N	N
Meng (2012)	EAHM formula for individual research	Prepared by Meng (2012)	Astragali Radix 30 g, Meliae Fructus 10 g, Pinelliae Tuber 10 g, Platycodonis Radix 30 g, Fritillariae Cirrhosae Bulbus 15 g, Trichosanthis Radix 30 g, Raphani Semen 20 g, Liriopis seu Ophiopogonis Tuber 15 g, Ginseng Radix 10 g, Psoraleae Semen 15 g, Loranthi Ramulus Et Folium 15 g, Glycyrrhizae Radix et Rhizoma 10 g, Scorpio 6 g, Lumbricus 15 g	*Astragalus mongholicus* Bunge [Fabaceae] 30 g, *Melia azedarach* L. [Meliaceae]10 g, *Pinellia ternata* (Thunb.) Makino [Araceae] 10 g, *Platycodon grandiflorus* (Jacq.) A.DC. [Campanulaceae] 30 g, *Fritillaria cirrhosa* D.Don [Liliaceae] 15 g, *Trichosanthes kirilowii* Maxim*.* [Cucurbitaceae] 30 g, *Raphanus raphanistrum* subsp. *sativus* (L.) Domin [Brassicaceae] 20 g, *Liriope muscari* (Decne.) L.H.Bailey [Asparagaceae] 15 g, *Panax ginseng* C.A.Mey. [Araliaceae] 10 g, *Cullen corylifolium* (L.) Medik. [Fabaceae] 15 g, *Taxillus chinensis* (DC.) Danser [Loranthaceae] 15 g, *Glycyrrhiza uralensis Fisch. ex DC.* [Fabaceae] 10 g, *Buthus martensii* Karsch 6 g, *Amynthas pectiniferus* 15 g	Decoction	N	N
Jiang et al. (2013)	EAHM formula for individual research	Prepared by Jiang et al. (2013)	Hedyotidis Herba 15 g, Scutellariae Barbatae Herba 20 g, Phellodendri Cortex 15 g, Rhei Radix et Rhizoma 15 g, Astragali Radix 20 g, Codonopsis Pilosulae Radix 20 g, Paeoniae Radix 20 g, Carthami Flos 15 g, Sophorae Radix 15 g, Sanguisorbae Radix 15 g, Aucklandiae Radix 9 g, Meliae Fructus 9 g, Caulis Sargentodoxae 15 g, Citri Unshius Pericarpium 15 g, Pulsatillae Radix 30 g, Sparganii Rhizoma 12 g, Curcumae Rhizoma 12 g, Ponciri Fructus Immaturus 12 g	*Scleromitrion diffusum (Willd.) R.J.Wang* [Rubiaceae] 15 g, *Scutellaria barbata D.Don* [Lamiaceae] 20 g, *Phellodendron amurense Rupr.* [Rutaceae] 15 g, *Rheum palmatum L.* [Polygonaceae] 15 g, *Astragalus mongholicus Bunge* [Fabaceae] 20 g, *Codonopsis pilosula (Franch.) Nannf.* [Campanulaceae] 20 g, *Paeonia lactiflora Pall.* [Paeoniaceae] 20 g, *Carthamus tinctorius L.* [Asteraceae] 15 g, *Sophora flavescens Aiton* [Fabaceae] 15 g, *Sanguisorba officinalis L.* [Rosaceae] 15 g, *Aucklandia costus* Falc. [Asteraceae]. 9 g, *Melia azedarach* L. [Meliaceae]. 9 g, *Sargentodoxa cuneata* (Oliv.) Rehder & E.H.Wilson [Lardizabalaceae] 15 g, *Citrus deliciosa* Ten. [Rutaceae] 15 g, *Pulsatilla chinensis* (Bunge) Regel [Ranunculaceae] 30 g, *Sparganium stoloniferum* (Buch.-Ham. ex Graebn.) Buch.-Ham. ex Juz. [Typhaceae] 12 g, *Curcuma phaeocaulis* Valeton [Zingiberaceae] 12 g, *Citrus trifoliata* L. [Rutaceae] 12 g	Decoction	N	N
Wang and Chen (2013)	Gexia Zhuyu decoction combined Shixiao powder	Prepared by Wang and Chen (2013)	Typhae Pollen 6 g, Trogopterorum Faeces 10 g, Linderae Radix 12 g, Persicae Semen 12 g, Moutan Radicis Cortex 12 g, Paeoniae Radix 12 g, Corydalis Tuber 12 g, Cyperi Rhizoma 12 g, Carthami Flos 12 g, Notoginseng Radix et Rhizoma 10 g, Scrophulariae Radix 12 g, Cynanchi Paniculati Radix Et Rhizoma 30 g	*Typha orientalis* C.Presl [Typhaceae] 6 g, *Trogopterus xanthipes* 10 g, *Lindera aggregata* (Sims) Kosterm. [Lauraceae] 12 g, *Prunus persica* (L.) Batsch [Rosaceae] 12 g, *Paeonia × suffruticosa* Andrews [Paeoniaceae] 12 g, *Paeonia lactiflora* Pall. [Paeoniaceae] 12 g, *Corydalis* ternata (Nakai) Nakai [Papaveraceae] 12 g, *Cyperus rotundus* L. [Cyperaceae] 12 g, *Carthamus tinctorius L*. [Asteraceae] 12 g, *Panax notoginseng (Burkill) F.H.Chen* [Araliaceae] 10g, *Scrophularia ningpoensis Hemsl.* [Scrophulariaceae] 12 g, *Vincetoxicum mukdenense Kitag.* [Apocynaceae] 30 g	Decoction	N	N
Chen H. et al. (2014)	Xuefu Zhuyu decoction	Prepared by Chen H. et al. (2014)	Persicae Semen 20 g, Carthami Flos 9 g, Angelicae Sinensis Radix 9 g, Rehmanniae Radix Recens 9 g, Achyranthis Radix 15 g, Cnidii Rhizoma 5 g, Paeoniae Radix 6 g, Platycodonis Radix 5 g, Bupleuri Radix 3 g, Scrophulariae Radix 6 g, Glycyrrhizae Radix et Rhizoma 6 g	*Prunus persica* (L.) Batsch [Rosaceae] 20 g, *Carthamus tinctorius* L. [Asteraceae] 9 g, *Angelica sinensis* (Oliv.) Diels [Apiaceae] 9 g, *Rehmannia glutinosa* (Gaertn.) DC. [Orobanchaceae] 9 g, *Achyranthes bidentata* Blume [Amaranthaceae] 15 g, *Conioselinum anthriscoides 'Chuanxiong'* [Apiaceae]. 5 g, *Paeonia lactiflora* Pall. [Paeoniaceae] 6 g, *Platycodon grandiflorus* (Jacq.) A.DC. [Campanulaceae] 5 g, *Bupleurum falcatum* L. [Apiaceae] 3 g, *Scrophularia ningpoensis* Hemsl. [Scrophulariaceae] 6 g, *Glycyrrhiza uralensis* Fisch. ex DC. [Fabaceae] 6 g	Decoction	N	N
Liu and Zhou (2014)	Buqi Huoxue decoction	Prepared by Liu and Zhou (2014)	Rehmanniae Radix Preparata 30 g, Rehmanniae Radix Recens 30 g, Dioscoreae Rhizoma 20 g, Angelicae Sinensis Radix 20 g, Cnidii Rhizoma 10 g, Ligustri Fructus 20 g, Ecliptae Herba 10 g, Thuja Orientalis Folium 10 g, c 3 g, Polygoni Multiflori Radix 30 g, Liquidambaris Fructus 30 g, Astragali Radix 30 g, Glycyrrhizae Radix et Rhizoma 10 g, Persicae Semen 10 g, Carthami Flos 10 g	*Rehmannia glutinosa* (Gaertn.) DC. [Orobanchaceae] 30 g, *Rehmannia glutinosa* (Gaertn.) DC. [Orobanchaceae] 30 g, *Dioscorea polystachya* Turcz. [Dioscoreaceae] 20 g, *Angelica sinensis* (Oliv.) Diels [Apiaceae] 20 g, *Conioselinum anthriscoides 'Chuanxiong'* [Apiaceae] 10 g, *Ligustrum lucidum* W.T.Aiton [Oleaceae] 20 g, *Eclipta prostrata* (L.) L. [Asteraceae] 10 g, *Platycladus orientalis* (L.) Franco [Cupressaceae] 10 g, *Actaea heracleifolia* (Kom.) J.Compton [Ranunculaceae] 3 g, *Reynoutria multiflora (Thunb.)* Moldenke [Polygonaceae] 30 g, *Liquidambar formosana* Hance [Altingiaceae] 30 g, *Astragalus mongholicus Bunge* [Fabaceae] 30 g, *Glycyrrhiza uralensis* Fisch. ex DC. [Fabaceae] 10 g, *Prunus persica* (L.) Batsch [Rosaceae] 10 g, *Carthamus tinctorius* L. [Asteraceae] 10 g	Decoction	N	N
Wan et al. (2014)	Compound Sangzhi mixture	Prepared by Wan et al. (2014)	Mori Ramulus 30 g, Sinomeni Caulis et Rhizoma 30 g, Piperis Kadsurae Caulis 30 g, Angelicae Sinensis Radix 15 g, Osterici seu Notopterygii Radix et Rhizoma 10 g, Araliae Continentalis Radix 10 g, Gentianae Macrophyllae Radix 10 g, Sinomeni Caulis et Rhizoma 10 g, Clematidis Radix 10 g, Cnidii Rhizoma 12 g	*Morus alba L.* [Moraceae] 30 g, *Sinomenium acutum (Thunb.) Rehder & E.H.Wilson* [Menispermaceae] 30 g, *Piper kadsura* (Choisy) Ohwi [Piperaceae] 30 g, *Angelica sinensis* (Oliv.) Diels [Apiaceae] 15 g, *Ostericum grosseserratum* (Maxim.) Kitag. [Apiaceae] 10 g, *Aralia continentalis* Kitag. [Araliaceae] 10 g, *Gentiana macrophylla* Pall. [Gentianaceae] 10 g, *Sinomenium acutum* (Thunb.) Rehder & E.H.Wilson [Menispermaceae] 10 g, *Clematis terniflora* var. *mandshurica* (Rupr.) Ohwi [Ranunculaceae] 10 g, *Conioselinum anthriscoides “Chuanxiong”* [Apiaceae] 12 g	Decoction	N	N
Song et al. (2015)	Bai Shao Zong Gan Jiao Nang	Commercial Supplier Ningbo Lihua Pharmacy Co., Ltd.	Paeoniae Radix	*Paeonia lactiflora* Pall. [Paeoniaceae]	Capsule	N	N
Chen et al. (2017)	Hugu Xiaoji prescription	Prepared by Chen et al. (2017)	Drynariae Rhizoma, Psoraleae Semen, Epimedii Herba, Arisaematis Rhizoma, Lumbricus, Scorpio	*Drynaria roosii* Nakaike [Polypodiaceae], *Cullen corylifolium* (L.) Medik. [Fabaceae], *Epimedium brevicornu* Maxim*.* [Berberidaceae], *Arisaema erubescens* (Wall.) Schott [Araceae], *Amynthas pectiniferus*, *Buthus martensii* Karsch	NR	N	N
Li et al. (2017b)	Xuefu Zhuyu decoction	Prepared by Li et al. (2017a)	Angelicae Sinensis Radix 9 g, Rehmanniae Radix Recens 9 g, Cnidii Rhizoma 9 g, Paeoniae Radix 9 g, Persicae Semen 12 g, Carthami Flos 9 g, Achyranthis Radix 12 g, Bupleuri Radix 6 g, Scrophulariae Radix 6 g, Platycodonis Radix 6 g, Glycyrrhizae Radix et Rhizoma 6 g	*Angelica sinensis* (Oliv.) Diels [Apiaceae] 9 g, *Rehmannia glutinosa* (Gaertn.) DC. [Orobanchaceae] 9 g, *Conioselinum anthriscoides “Chuanxiong”* [Apiaceae] 9 g, *Paeonia lactiflora* Pall. [Paeoniaceae] 9 g, *Prunus persica* (L.) Batsch [Rosaceae] 12 g, *Carthamus tinctorius* L. [Asteraceae] 9 g, *Achyranthes bidentata* Blume [Amaranthaceae] 12 g, *Bupleur* *um falcatum* L. [Apiaceae] 6 g, *Scrophularia ningpoensis* Hemsl. [Scrophulariaceae] 6 g, *Platycodon grandiflorus* (Jacq.) A.DC. [Campanulaceae] 6 g, *Glycyrrhiza uralensis* Fisch. ex DC. [Fabaceae] 6 g	Decoction	N	N
Li et al. (2017a)	Gexia Zhuyu decoction	Prepared by Li et al. (2017b)	Trogopterorum Faeces 6 g, Angelicae Sinensis Radix 9 g, Cnidii Rhizoma 9 g, Paeoniae Radix 9 g, Persicae Semen 9 g, Carthami Flos 6 g, Scrophulariae Radix 6 g, Linderae Radix 6 g, Cyperi Rhizoma 6 g, Moutan Radicis Cortex 9 g, Corydalis Rhizoma 9 g, Meliae Fructus 9 g, Akebiae Fructus 9 g, Salviae Miltiorrhizae Radix 15 g, Curcumae Rhizoma 9 g, Curcumae Longae Rhizoma 9 g, Curcumae Radi 6 g, Glycyrrhizae Radix et Rhizoma 6 g	*Trogopterus xanthipes* 6 g, Angelica sinensis (Oliv.) Diels [Apiaceae] 9 g, *Conioselinum anthriscoides “Chuanxiong”* [Apiaceae] 9 g, *Paeonia lactiflora* Pall. [Paeoniaceae] 9 g, *Prunus persica* (L.) Batsch [Rosaceae] 9 g, *Carthamus tinctorius* L. [Asteraceae] 6 g, *Scrophularia ningpoensis* Hemsl. [Scrophulariaceae] 6 g, *Lindera aggregata* (Sims) Kosterm*.* [Lauraceae] 6 g, *Cyperus rotundus* L. [Cyperaceae] 6 g, *Paeonia × suffruticosa Andrews* [Paeoniaceae] 9 g, *Corydalis yanhusuo* (Y.H.Chou & Chun C.Hsu) W.T.Wang ex Z.Y.Su & C.Y.Wu [Papaveraceae] 9 g, *Melia azedarach* L*.* [Meliaceae] 9 g, *Akebia quinata* (Thunb. ex Houtt.) Decne. [Lardizabalaceae] 9 g, *Salvia miltiorrhiza* Bunge [Lamiaceae] 15 g, *Curcuma phaeocaulis* Valeton [Zingiberaceae] 9 g, *Curcuma longa* L. [Zingiberaceae] 9 g, *Curcuma aromatica Salisb.* [Zingiberaceae] 6 g, *Glycyrrhiza uralensis Fisch. ex DC.* [Fabaceae] 6 g	Decoction	N	N
Bao (2018)	Xuefu Zhuyu decoction	Prepared by Bao (2018)	Persicae Semen 20 g, Codonopsis Pilosulae Radix 15 g, Astragali Radix 15 g, Atractylodis Rhizoma Alba 12 g, Carthami Flos 10 g, Angelicae Sinensis Radix 10 g, Achyranthis Radix 9 g, Rehmanniae Radix Recens 9 g, Paeoniae Radix 6 g, Glycyrrhizae Radix et Rhizoma 6 g, Scrophulariae Radix 6 g	*Prunus persica* (L.) Batsch [Rosaceae] 20 g, *Codonopsis pilosula* (Franch.) Nannf. [Campanulaceae] 15 g, *Astragalus mongholicus* Bunge [Fabaceae] 15 g, *Atractylodes macrocephala* Koidz. [Asteraceae] 12 g, *Carthamus tinctorius* L. [Asteraceae] 10 g, *Angelica sinensis* (Oliv.) Diels [Apiaceae] 10 g, *Achyranthes bidentata* Blume [Amaranthaceae] 9 g, *Rehmannia glutinosa* (Gaertn.) DC. [Orobanchaceae] 9 g, *Paeonia lactiflora* Pall. [Paeoniaceae] 6 g, *Glycyrrhiza uralensis* Fisch. ex DC. [Fabaceae] 6 g, *Scrophularia ningpoensis* Hemsl. [Scrophulariaceae] 6 g	Decoction	N	N
Dong et al. (2018)	Huachansu Jiaonang	NR	Bufonis Venenum	*Bufo bufo gargarizans* Cantor	Capsule	N	N
Miu and Quan (2018)	Huachansu Jiaonang	Commercial supplier Eastantai pharmaceutical co.,Ltd.	Bufonis Venenum	*Bufo bufo gargarizans* Cantor	Capsule	N	N
Ouyang (2018)	Modified Shaogan Fuzi decoction	Prepared by Ouyang (2018)	Glycyrrhizae Radix et Rhizoma 20 g, Paeoniae Radix 60 g, Aconiti Lateralis Radix Preparata 15 g, Cuscutae Semen 20 g, Codonopsis Pilosulae Radix 20 g, Cannabis Semen 15 g, Curcumae Radi 10 g, Agrimoniae Herba 15 g, Angelicae Sinensis Radix 10 g, Corydalis Tuber 15 g	*Glycyrrhiza uralensis* Fisch. ex DC. [Fabaceae] 20 g, *Paeonia lactiflora* Pall. [Paeoniaceae] 60 g, *Aconitum carmichaelii* Debeaux [Ranunculaceae] 15 g, *Cuscuta chinensis* Lam. [Convolvulaceae] 20 g, *Codonopsis pilosula (Franch.)* Nannf*.* [Campanulaceae] 20 g, *Cannabis sativa* L. [Cannabaceae] 15 g, *Curcuma aromatica* Salisb. [Zingiberaceae] 10 g, *Agrimonia pilosa* Ledeb. [Rosaceae] 15 g, *Angelica sinensis* (Oliv.) Diels [Apiaceae] 10 g, *Corydalis ternata* (Nakai) Nakai [Papaveraceae] 15 g	Decoction	N	N
Liu (2020)	Liujunzi decoction	Prepared by Liu (2020)	Pseudostellariae Radix 15 g, Codonopsis Pilosulae Radix 15 g, Astragali Radix 30 g, Poria Sclerotium 15 g, Hedyotidis Herba 15 g, Salviae Miltiorrhizae Radix 20 g, Dioscoreae Rhizoma 25 g, Atractylodis Rhizoma Alba 15 g, Angelicae Sinensis Radix 15 g, Pinelliae Tuber 15 g, Paeoniae Radix 15 g, Sparganii Rhizoma 10 g, Curcumae Rhizoma 10 g, Citri Unshius Pericarpium 15 g, Glycyrrhizae Radix et Rhizoma 25 g	*Pseudostellaria heterophylla* (Miq.) Pax [Caryophyllaceae] 15 g, *Codonopsis pilosula* (Franch.) Nannf. [Campanulaceae] 15 g, *Astragalus mongholicus* Bunge [Fabaceae] 30 g, *Poria cocos* Wolf 15 g, *Scleromitrion diffusum* (Willd.) R.J.Wang [Rubiaceae] 15 g, *Salvia miltiorrhiza* Bunge [Lamiaceae] 20 g, *Dioscorea polystachya* Turcz. [Dioscoreaceae] 25 g, *Atractylodes macrocephala* Koidz. [Asteraceae] 15 g, *Angelica sinensis* (Oliv.) Diels [Apiaceae] 15 g, *Pinellia ternata* (Thunb.) Makino [Araceae] 15 g, *Paeonia lactiflora* Pall. [Paeoniaceae] 15 g, *Sparganium stoloniferum* (Buch.-Ham. ex Graebn.) Buch.-Ham. ex Juz. [Typhaceae] 10 g, *Curcuma phaeocaulis* Valeton [Zingiberaceae] 10 g, *Citrus deliciosa* Ten. [Rutaceae] 15 g, *Glycyrrhiza uralensis* Fisch. ex DC. [Fabaceae] 25 g	Decoction	N	N
Yang (2020)	EAHM formula for individual research	Prepared by Yang (2020)	Astragali Radix 30 g, Platycodonis Radix 30 g, Trichosanthis Radix 30 g, Fritillariae Cirrhosae Bulbus 15 g, Liriopis seu Ophiopogonis Tuber 15 g, Loranthi Ramulus Et Folium 15 g, Psoraleae Semen 15 g, Lumbricus 15 g, Meliae Fructus 10 g, Pinelliae Tuber 10 g, Glycyrrhizae Radix et Rhizoma 10 g, Ginseng Radix 10 g, Raphani Semen 20 g, Scorpio 6 g	*Astragalus mongholicus Bunge* [Fabaceae] 30 g, *Platycodon grandiflorus* (Jacq.) A.DC. [Campanulaceae] 30 g, *Trichosanthes kirilowii* Maxim*.* [Cucurbitaceae] 30 g, *Fritillaria cirrhosa* D.Don [Liliaceae] 15 g, *Liriope muscari* (Decne.) L.H.Bailey [Asparagaceae] 15 g*, Taxillus chinensis* (DC.) Danser [Loranthaceae] 15 g, *Cullen corylifolium* (L.) Medik. [Fabaceae] 15 g, *Amynthas pectiniferus* 15 g, *Melia azedarach* L*.* [Meliaceae] 10 g, *Pinellia ternata* (Thunb.) Makino [Araceae] 10 g, *Glycyrrhiza uralensis* Fisch. ex DC. [Fabaceae] 10 g, *Panax ginseng* C.A.Mey. [Araliaceae] 10 g, *Raphanus raphanistrum* subsp. *sativus* (L.) Domin [Brassicaceae] 20 g, *Buthus martensii* Karsch 6 g	Decoction	N	N
Liang et al. (2021)	Huachansu Jiaonang	Commercial Aupplier Eastantai Pharmaceutical Co., Ltd.	Bufonis Venenum	*Bufo bufo gargarizans* Cantor	Capsule	N	N

EAHM, East Asian herbal medicine; NR, not reported.

**TABLE 6:** The top 10 frequency herbal ingredients prescribed for cancer pain.

EAHM (Scientific name)	Frequency of utilization	Relative frequency (%)
*Glycyrrhiza uralensis* Fisch. ex DC. [Fabaceae]	20	52.63
*Paeonia lactiflora* Pall. [Paeoniaceae]	19	50.00
*Angelica sinensis* (Oliv.) Diels [Apiaceae]	13	34.21
*Prunus persica* (L.) Batsch [Rosaceae]	11	28.94
*Corydalis ternata* (Nakai) Nakai [Papaveraceae]	10	26.31
*Carthamus tinctorius* L. [Asteraceae]	9	23.68
*Pinellia ternata* (Thunb.) Makino [Araceae]	8	21.05
*Cullen corylifolium* (L.) Medik. [Fabaceae]	8	21.05
*Buthus martensii* Karsch	8	21.05
*Scrophularia ningpoensis* Hemsl. [Scrophulariaceae]	8	21.05

EAHM: East Asian herbal medicine.

#### 3.9.2 Apriori Algorithm-Based Association Rule Analysis

**FIGURE 8:**
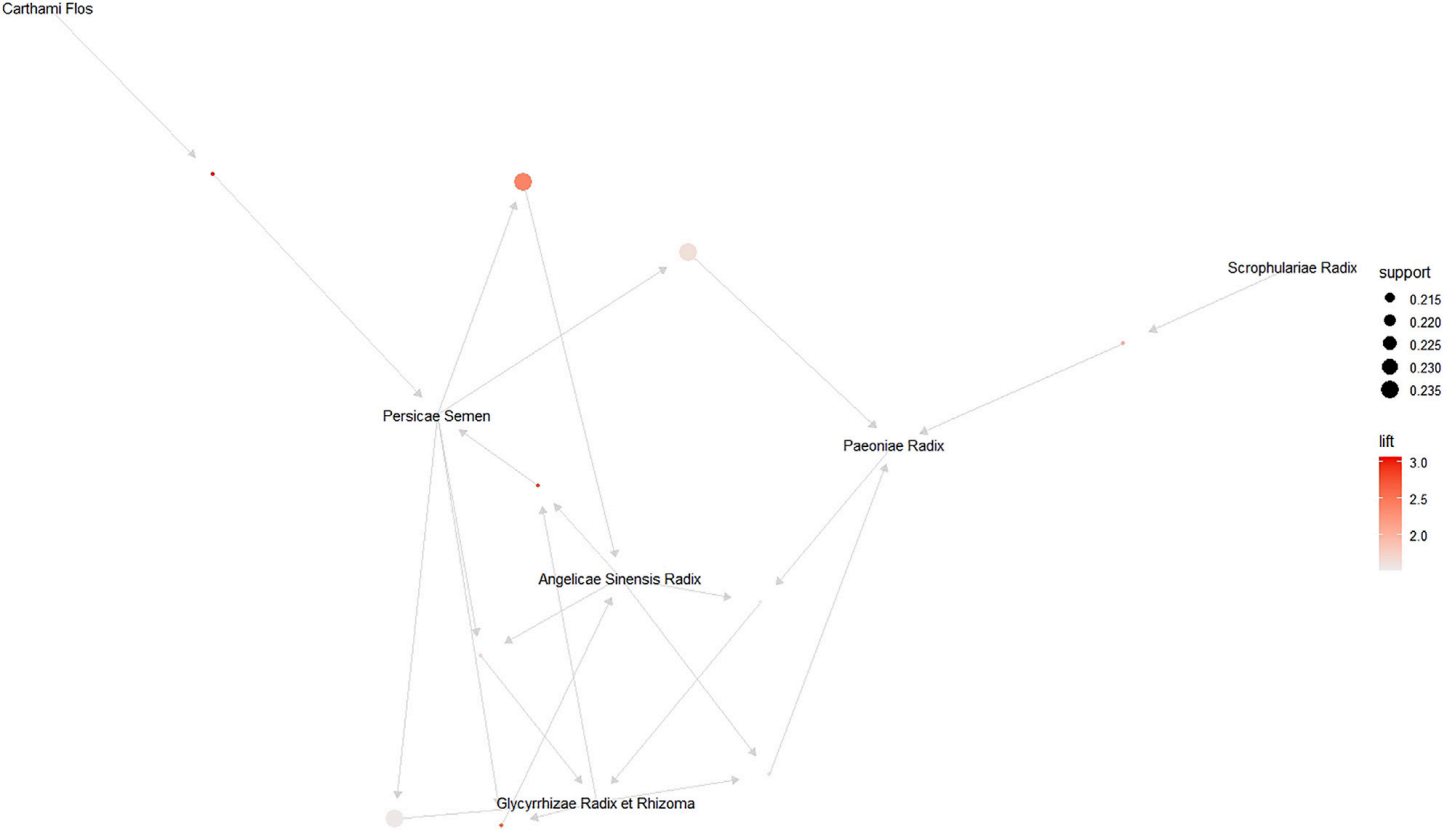
Network graph of the association rules in the meta-analysis of EAHM prescribed for cancer pain.

Based on ingredient data from 38 EAHM formulations and 125 herbs included in this study, 10 association rules were identified in the analysis (Table 7). Based on the identified association rule, a scatter plot with support value on the x-axis and the confidence value on the y-axis was drawn to explore the distribution of lift values (Supplementary Figure S5). In this scatter plot, the depth of the dot color representing each association rule indicates the lift value. Through this, it was possible to observe the fact that the distribution of the overall lift value was distributed between 1.53 and 3.07. Meanwhile, a grouping matrix diagram was presented to examine the general distribution of the identified association rule (Supplementary Figure S6). The abscissas represent 7 clusters, and they represent items generated by 10 association rules. The depth of color inside the circle represents the degree of lift, and the circle size represents the degree of support. As a result of the above analysis, the three rules showing the highest support value of 2.37 were # 3 {*Prunus persica* (L.) Batsch [Rosaceae]} => {*Angelica sinensis* (Oliv.) Diels [Apiaceae]}, # 4{*Prunus persica* (L.) Batsch [Rosaceae]} => {*Paeonia lactiflora* Pall*.* [Paeoniaceae]}, and # 5 {*Prunus persica* (L.) Batsch [Rosaceae]} => {*Glycyrrhiza uralensis* Fisch. ex DC. [Fabaceae]*}*. On the other hand, the rule showing the highest confidence value of 1.00 was #1 {*Scrophularia ningpoensis* Hemsl. [Scrophulariaceae]} => {*Paeonia lactiflora* Pall. [Paeoniaceae]}, and the herb patterns that can be predicted to increase the probability of significant association with lift value exceeding 2.0 are # 2 {*Carthamus tinctorius* L. [Asteraceae]} => {*Prunus persica* (L.) Batsch [Rosaceae]}, # 3 {*Prunus persica* (L.) Batsch [Rosaceae]} => {*Angelica sinensis* (Oliv.) Dlels}, # 7 {*Glycyrrhiza uralensis* Fisch. ex DC. [Fabaceae], *Prunus persica* (L.) Batsch [Rosaceae]} => *Angelica sinensis* (Oliv.) Diels [Apiaceae]}, and # 8 {*Angelica sinensis* (Oliv.) Dlels, *Glycyrrhiza uralensis* Fisch. ex *DC.* [Fabaceae]} => {*Prunus persica* (L.) Batsch [Rosaceae]}. Through the above analysis results, it was revealed that *Glycyrrhiza uralensis* Fisch. ex *DC.* [Fabaceae], *Angelica sinensis* (Oliv.) Diels [Apiaceae], and *Paeonia lactiflora* Pall. [Paeoniaceae] were selected as the central herbs for treating cancer pain with a correlation with *Prunus persica* (L.) Batsch [Rosaceae]. However, since *Glycyrrhiza uralensis* Fisch. ex *DC.* [Fabaceae] is also included in several other association rules, the potential core herb combination formed here could be regarded as *Prunus persica* (L.) Batsch [Rosaceae] - *Angelica sinensis* (Oliv.) Diels [Apiaceae] and *Prunus persica* (L.) Batsch [Rosaceae] - *Paeonia lactiflora* Pall. [Paeoniaceae]. Other influential herb pairs were *Carthamus tinctorius L.* [Asteraceae] - *Prunus persica* (L.) Batsch [Rosaceae] and *Scrophularia ningpoensis* Hemsl. [Scrophulariaceae] - *Paeonia lactiflora* Pall. [Paeoniaceae]. As a result, the herbs constituting the core ingredients of EAHM used in this study for cancer pain in patients were *Prunus persica* (L.) *Batsch* [Rosaceae], *Angelica sinensis* (Oliv.) Diels [Apiaceae], *Carthamus tinctorius* L. [Asteraceae], *Paeonia lactiflora* Pall. [Paeoniaceae], *Scrophularia ningpoensis* Hemsl. [Scrophulariaceae], and *Glycyrrhiza uralensis* Fisch. ex *DC.* [Fabaceae]. The relationship of these association rules is presented through a network graph (Figure 8).

**TABLE 7:** Apriori algorithm-based association rules in the meta-analysis of EAHM prescribed for cancer pain.

No	Associations rules	Support	Confidence	Lift
1	{Scrophularia *ningpoensis* Hemsl. [Scrophulariaceae]*}* => {*Paeonia lactiflora* Pall. [Paeoniaceae]}	0.211	1.000	2.000
2	{Carthamus *tinctorius L.* [Asteraceae]} => {*Prunus persica* (L.) Batsch [Rosaceae]}	0.211	0.889	3.070
3	{*Prunus persica* (L.) Batsch [Rosaceae]} => {*Angelica sinensis* (Oliv.) Diels [Apiaceae]}	0.237	0.818	2.392
4	{*Prunus persica* (L.) Batsch [Rosaceae]} => {*Paeonia lactiflora* Pall. [Paeoniaceae]}	0.237	0.818	1.636
5	{*Prunus persica* (L.) Batsch [Rosaceae]} => {*Glycyrrhiza uralensis* Fisch. ex DC. [Fabaceae]}	0.237	0.818	1.555
6	{*Angelica sinensis *(Oliv.) Diels [Apiaceae], *Prunus persica* (L.) Batsch [Rosaceae]} => {*Glycyrrhiza uralensis* Fisch. ex DC. [Fabaceae]}	0.211	0.889	1.689
7	{*Glycyrrhiza uralensis* Fisch. ex DC. [Fabaceae], *Prunus persica* (L.) Batsch [Rosaceae]} => {*Angelica sinensis* (Oliv.) Diels [Apiaceae]}	0.211	0.889	2.600
8	{*Angelica sinensis* (Oliv.) Diels [Apiaceae], *Glycyrrhiza uralensis* Fisch. ex DC. [Fabaceae]} => {*Prunus persica* (L.) Batsch [Rosaceae]}	0.211	0.800	2.764

## 4 Discussion

### 4.1 Summary of the Main Finding

In this systematic review, the effects and safety of EAHM as combined therapy or monotherapy versus conventional medicine for primary cancer pain were assessed. Overall, EAHM as combined therapy showed superior effects on cancer pain to those of conventional medicine in pain intensity, response rate, duration of pain relief, performance status, and opioid usage. Additionally, EAHM was generally safe and well-tolerable for patients with cancer. Patients treated with EAHM appeared to experience fewer incidence rates of AEs. Therefore, EAHM-combined therapy can be considered a worthy option based on the data of this study in the management of cancer pain. Regarding the various EAHM prescription data included in this study, as a result of the association rule mining, *Prunus persica* (L.) Batsch [Rosaceae], *Angelica sinensis* (Oliv.) Diels [Apiaceae], *Carthamus tinctorius* L. [Asteraceae], *Paeonia lactiflora* Pall. [Paeoniaceae], *Scrophularia ningpoensis* Hemsl. [Scrophulariaceae], and *Glycyrrhiza uralensis* Fisch. ex *DC.* [Fabaceae] were identified as core herb ingredients. At the same time, four combinations of herb pairs considered to have potential significance for cancer pain were *Prunus persica* (L.) Batsch [Rosaceae] - *Angelica sinensis* (Oliv.) Diels [Apiaceae], *Prunus persica* (L.) Batsch [Rosaceae] - *Paeonia lactiflora* Pall. [Paeoniaceae], *Carthamus tinctorius* L. [Asteraceae] - *Prunus persica* (L.) Batsch [Rosaceae], and *Scrophularia ningpoensis* Hemsl. [Scrophulariaceae] - *Paeonia lactiflora* Pall. [Paeoniaceae]. Information on these core herbs is expected to have value as a useful hypothesis for future drug development research using EAHM.

**TABLE 8:** Potential mechanism of core herbs included in this review

First author (Year)	Scientific name of herbal materials	Possible active ingredients	Target cell line or animal model	Possible mechanisms
Kwon et al. (2003)	*Prunus persica* (L.) Batsch [Rosaceae]	Amygdalin (active D-form)	Human promyelocytic leukemia (HL-60) cells	Antiproliferative effect: cytotoxic to HL-60 cells with IC50 of 6.4 mg/ml in the presence of 250 nM of beta-glucosidase as induced nuclear morphology changes and internucleosomal DNA fragmentation
Chiu et al. (2017)	*Angelica sinensis* (Oliv.) Diels [Apiaceae]	N-butylidenephthalide	Human bladder cancer cell lines TCCSUP, 5637, T24, and BFTC (BFTC 905)	Antiproliferative effect: bladder cancer cell death in a time- and dose-dependent manner and induced apoptosis via the activation of caspase-9 and caspase-3, migration of bladder cancer cells suppression, upregulation of E-cadherin and downregulation of N-cadherin, suppressed BFTC xenograft tumor growth
Zhang et al. (2019)	*Carthamus tinctorius* L. [Asteraceae]	Hydroxysafflor yellow A	H22 tumor-bearing mice HepG2 cells	Anti-angiogenic effect: MMP-2 and MMP-9 decrease in H22-transplanted tumor tissue, COX-2 expression was reduced via p38MAPK|ATF-2 signaling pathway, suppression of p38 activation by SB203580 decreased the HepG2 cell viability, proliferation, and migration
Zhang et al. (2016)	*Paeonia lactiflora* Pall. [Paeoniaceae]	Paeoniflorin	Human breast cancer cell lines (MDA-MB-231 and MCF-7)	Antiproliferative effect: inhibits the proliferation and invasion of breast cancer cells through suppressing the Notch-1 signaling pathway
Sheu et al. (2015)	*Scrophularia ningpoensis* Hemsl. [Scrophulariaceae]	Harpagoside	Microglia cells harvested from neonatal ICR mice were activated by exposure to hypoxia	Antiproliferative effect: scavenge hypoxia-enhanced inflammatory genes expression (COX-2, IL-1β and IL-6 genes) and NO synthesis of microglial cells through the NF-κB signaling pathway
Wang S. J. et al. (2013)	*Glycyrrhiza uralensis* Fisch. ex DC. [Fabaceae]	Isoliquiritigenin	Human umbilical vein endothelial cells (HUVECs)	Anti-angiogenic effect: inhibit VEGF expression in breast cancer cells via promoting HIF-1α proteasome degradation, suppressed VEGF/VEGFR-2 signaling pathway

ATF-2, activating transcription factor 2; BFTC, bladder transitional cell carcinoma; COX-2, cyclooxygenase 2; ERK, extracellular-signal-regulated kinase; HIF-1α, hypoxia-inducible factor-1α; HL, human leukemia; IC50, inhibitory concentration 50; IL, interleukin; JNK, c-Jun N-terminal kinase; MAPK, mitogen-activated protein kinase; MMPs, matrix metalloproteinases; NF-κB, nuclear factor kappa-light-chain-enhancer of activated B cells; NO, nitric oxide; VEGF, vascular endothelial growth factor; VEGFR, vascular endothelial growth factor receptor.

### 4.2 Limitations

Clinicians and researchers should note the following limitations before utilizing the results of this systematic review. Firstly, the outcome measures that should be prioritized in pain management of various diseases, including cancer, is Minimum Clinically Important Difference (MCID) in continuous pain intensity. In particular, the significance of cancer pain is greater in that the severity of symptoms itself has a significant impact on the patient’s prognosis. However, in our review, only 12 studies measured continuous pain intensity, and MCID information was not addressed in any of the studies. The response rate adopted by many studies may be a criterion for determining whether an effect occurs, but it cannot replace MCID. For this reason, it is expected that more reliable EAHM efficacy for cancer pain will be possible only when more EAHM clinical trials considering MCID due to continuous pain intensity are conducted. Secondly, the effect of EAHM monotherapy on cancer pain examined in this study not only lacks evidence, despite some positive findings compared to conventional medicine but also lacks consistency in the reported results. Therefore, it was not possible to draw specific conclusions about the effects of EAHM monotherapy on cancer pain only from the studies included in this review. To solve this problem, clinical trials using placebo control and double-blind methodologies need to be additionally performed in the future. Thirdly, the methodological quality of the clinical trials included in this study is generally poor. It is believed that many studies lack explanations for random allocation concealment, cannot confirm pre-registered protocols, and do not employ blinding of participants and outcome assessors. For this reason, it is difficult to reach a rigorous conclusion even if the review includes relatively large sample data and primary tests. Therefore, until a clinical trial with an improved design is added, the conclusions of this review should be taken with caution, considering the information of individual included studies when used in clinical practice. Furthermore, high heterogeneity was observed in the continuous outcomes of this study. This heterogeneity reduces the strength of the synthesized evidence. In this review, meta-regression and subgroup analysis could be performed only on continuous pain intensity because only a few studies adopted a continuous outcome measure, and even through this, detailed causes of heterogeneity could not be identified. Nevertheless, it is not difficult to infer from reviews dealing with EAHM that a major cause of heterogeneity is strongly related to the extreme variability in the composition and dosage of herbal formulations used in individual clinical trials. This leads to serious discrepancies between mediations except for the commonality of “East Asian herbal medicine combination.” In this review, association rule analysis was performed on herb data to overcome this heterogeneity problem partially and to derive more useful information. In the future systematic review of similar topics, it is expected that the data mining method will be actively used to derive additional valuable information consistent with the characteristics of EAHM.

### 4.3 Implications of Clinical Practices

Evidence from the present study supports that concomitant use of EAHM may be considered for the management of cancer pain. The primary finding in this review supporting this is that EAHM as combined therapy provides a significant benefit in improving the response rate and pain intensity of cancer pain. This can be consistent with two previous systematic reviews of similar topics (Wang S.-J. et al., 2013; Lee et al., 2015). However, pain as the secondary symptom caused by anti-tumor treatment (e.g., surgery, chemotherapy, or radiotherapy) was excluded from the scope of the study, and pain caused by cancer itself was set as the target disease in this review. In addition, considering that EAHM is most widely used as a drug commonly taken orally, outcomes by topical applications such as injection, herbal bath, or herbal compression were not included. The characteristic of this review is that it is differentiated from previous studies related to the subject. In addition, considering that safety in cancer treatment is the major issue that patients are concerned about, the incidence rate of AEs was examined by category of symptoms with high frequency in the meta-analysis. Another valuable finding in our review was that the utilization of EAHM could potentially be involved in significantly lowering the odds of adverse events through this analysis. These characteristics of our study suggested that the EAHM as combined therapy with a conventional approach may be a better strategy for cancer patients with pain who are partially insensitive to the conventional medicine alone or are intolerant to opioids and other analgesic drugs. However, a direct comparison between conventional medicine and EAHM showed a potentially better result in response rate but was not statistically significant. Regarding continuous pain intensity, EAHM monotherapy showed a significant lower effect, but the number of trials related to it was minimal. Therefore, it could not be concluded whether EAHM monotherapy can be used as an alternative to conventional treatment for the management of cancer pain.

In the herb data constituting the EAHM prescription of this study, four significant herb pairs and six high-frequency individual herbal medicines were identified. As seen in Table 7, the herbal medicines that form the core herb patterns in this study are expected to contribute to various findings of cancer patients not only in clinical research data but also in terms of mechanisms in modern pharmacological studies. In addition, the two-herb combination pattern identified in this study may be regarded as a frequently used herb pair due to their clinical value. EAHM is generally administered in the form of a multi-herb formula, and herb pairs are used as a basic unit for constructing patient-specific dosages and useful prescriptions (Wang et al., 2012). In addition, the herb pair concept has been widely used recently as a hypothesis to efficiently develop new drugs while reinterpreting existing clinical data from different angles by utilizing advanced research methodologies such as network pharmacology (Li et al., 2011; Mi et al., 2020). From this point of view, clinicians can incorporate the core herb combination information identified in this study into their decision-making.

### 4.4 Implications of Mechanism Research

The mechanism of action of EAHM, which solves various pathological problems in the human body at a systemic level through the action of multi-compounds on multi-targets, is being explored in more detail through recent scientific studies (Zhou et al., 2016). The multi-components of EAHM show a better effect by reducing toxicity and side effects due to the synergistic effect between various compounds in the process of acting on multiple targets. The concepts of “Gun-Shin-Jwa-Sa” (King-Retainer-Officer-Messenger, 君臣佐使 in Chinese characters) and herb pairs are the main prescribing principles of EAHM. To achieve the desired benefits and/or limit side effects of EAHM, use the “Gun-Shin-Jwa-Sa” principle. The key herb in an EAHM formula is “Gun,” which has a greater ratio of directly acting the disease. “Shin” is an adjuvant herb used to enhance the therapeutic impact of the main herb or to target the symptoms that come with it. “Jwa” is commonly used to reduce the EAHM formula’s negative effects. The herb “Sa” directs the active components to their intended organs or harmonizes their effects. Meanwhile, herb pair is a one-of-a-kind combination of two herbs that is the smallest unit of the EAHM formula and plays the most significant role in achieving synergy (Wang et al., 2012; Kim et al., 2015; Zhou et al., 2016). The four core herbal combination patterns explored in the results of this study, *Prunus persica* (L.) Batsch [Rosaceae] - *Angelica sinensis* (Oliv.) Diels [Apiaceae], *Prunus persica* (L.) Batsch [Rosaceae] - *Paeonia lactiflora Pall.* [Paeoniaceae], *Carthamus tinctorius* L. [Asteraceae] - *Prunus persica* (L.) Batsch [Rosaceae], and *Scrophularia ningpoensis* Hemsl. [Scrophulariaceae] - *Paeonia lactiflora* Pall. [Paeoniaceae], could also be predicted to have these benefits. For example, in 2012, it was reported that the major volatile component identified in the *Carthamus tinctorius* L. [Asteraceae] - *Prunus persica* (L.) Batsch [Rosaceae] extract combination in hot water was completely different from that of each single herb (Fu et al., 2012). Based on these results, the authors explained that the pharmacologically active compounds of the two-herb pairs recipe might be different from those of the single herbs which make them up. Data from another study examining the effects of *Carthamus tinctorius* L. [Asteraceae] - *Prunus persica* (L.) Batsch [Rosaceae] combination revealed that the herb pair could control liver inflammation and fibrosis by inhibiting pathological angiogenesis and hepatic fibrosis (Xi et al., 2016). This can be regarded as an example of the individual pharmacological activities of *Carthamus tinctorius* L. [Asteraceae] and *Prunus persica* (L.) Batsch [Rosaceae] that are strengthened through the synergistic effect of the mechanism discussed above. However, association rule mining is literally just a search tool for core patterns and cannot prove a causal relationship (Agrawal et al., 1993). Therefore, the herbal combination patterns identified in this review can be meaningful at the level of a valuable research hypothesis that needs to be verified through follow-up studies on whether they actually have amplified synergistic effects on cancer pain.

It is also a predictable mechanism that the efficacy of individual drugs from different angles acts simultaneously, acting on the complex pathology of cancer pain. In the case of *Paeonia lactiflora* Pallas, it is already known that it has potential effectiveness in various types of cancer, such as bladder tumor and lung cancer, based on several mechanistic studies (Lin MY. et al., 2016; Ma et al., 2021). At the same time, the combination of *Paeonia lactiflora* Pall. [Paeoniaceae] and *Glycyrrhiza uralensis* Fisch. ex DC. [Fabaceae], which is one of the key herbs in this study, is the “Jakyak-Gamcho decoction” (Shaoyao-Gancao-Tang in Chinese and Shakuyaku-Kanzo-To in Japanese), which is supported by reports that the herb combination is involved in various pain, associated with signaling pathways through recent network pharmacology study (Lee et al., 2020). In the above, it has been elucidated that *Scrophularia ningpoensis* Hemsl. [Scrophulariaceae], which forms a core combination with *Paeonia lactiflora* Pall. [Paeoniaceae], induces apoptosis of cancer cells by restoring anoikis sensitivity via disrupting focal adhesion action (Kim et al., 2017). As can be seen here, it is also important to specifically identify the pharmacological properties of individual drugs in order to select a meaningful core herbal combination. Considering *Prunus persica* (L.) Batsch [Rosaceae] contained in several core herb combinations, the active ingredient, which is amygdalin, is thought to contribute to the antiproliferative effects on tumor cells (Kwon et al., 2003). On the other hand, in the case of *Angelica sinensis* (Oliv.) Diels [Apiaceae], *Paeonia lactiflora* Pall. [Paeoniaceae], and *Scrophularia ningpoensis* Hemsl. [Scrophulariaceae], similar effects have been reported based on the action of each active ingredient, such as N-butylidenephthalide, paeoniflorin, and harpagoside (Sheu et al., 2015; Zhang et al., 2016; Chiu et al., 2017). In the case of *Carthamus tinctorius* L. [Asteraceae] - *Prunus persica* (L.) Batsch [Rosaceae] and *Glycyrrhiza uralensis* Fisch. ex DC. [Fabaceae], the anti-angiogenic effects were also related to the respective active ingredients hydroxysafflor yellow A and isoliquiritigenin (Wang Z. et al., 2013; Zhang et al., 2019). As previously discussed, it can be estimated that the action of EAHM at the individual component level and the synergistic effect through the complex action were combined to affect cancer pain caused by various pathologies and causes. It is reasonable to assume that these may be related to the positive clinical outcomes observed in this review. Therefore, it is worth specifically examining which herb combination can be used more effectively and safely for cancer pain compared to other individual herbs and herb pairs in future research and drug development.

## 5 Conclusion

This systematic review supports that EAHM therapy can minimize adverse events for upper and lower gastrointestinal reactions, such as nausea and constipation. Moreover, this meta-analysis demonstrated that EAHM combined with conventional medicine showed significantly better outcomes in response rate, continuous pain intensity, total duration of pain relief, performance status, opioid usage, and incidence of adverse events than prescribing conventional medicine alone. Furthermore, EAHM-combined therapy and monotherapy may result in a decrease in neurological side effects, such as drowsiness and headache, when treating cancer patients.

Considering the association rules on herb pairs, the four combinations of herb pairs, which were *Prunus persica* (L.) Batsch [Rosaceae] - *Angelica sinensis* (Oliv.) Diels [Apiaceae], *Prunus persica* (L.) Batsch [Rosaceae] - *Paeonia lactiflora* Pall. [Paeoniaceae], *Carthamus tinctorius* L. [Asteraceae] - *Prunus persica* (L.) Batsch [Rosaceae], and *Scrophularia ningpoensis* Hemsl. [Scrophulariaceae] - *Paeonia lactiflora* Pall. [Paeoniaceae], have been widely used among cancer treatment-related herbs. Besides, when one particular herb is employed to decrease cancer pain, it is likely that another herb may be used. Hence, they may be salutary for cancer patients to release cancer-related pain.

However, additional RCTs with a more valid outcome measure and an appropriate double-blind method should be additionally performed to draw more firm conclusions. Separately, it is considered worthwhile to conduct a follow-up study to verify the specific target and clinical superiority of the core herb combination pattern derived from this review.

## Data Availability

The original contributions presented in the study are included in the article/Supplementary Material; further inquiries can be directed to the corresponding authors.
