# Supplementary figures and images for: East Asian Herbal Medicine to Reduce Primary Pain and Adverse Events in Cancer Patients : A Systematic Review and Meta-Analysis With Association Rule Mining to Identify Core Herb Combination
Source: Front Pharmacol. 2022 Jan 17;12:800571. doi: 10.3389/fphar.2021.800571 (PMC8802093; doi:10.3389/fphar.2021.800571)

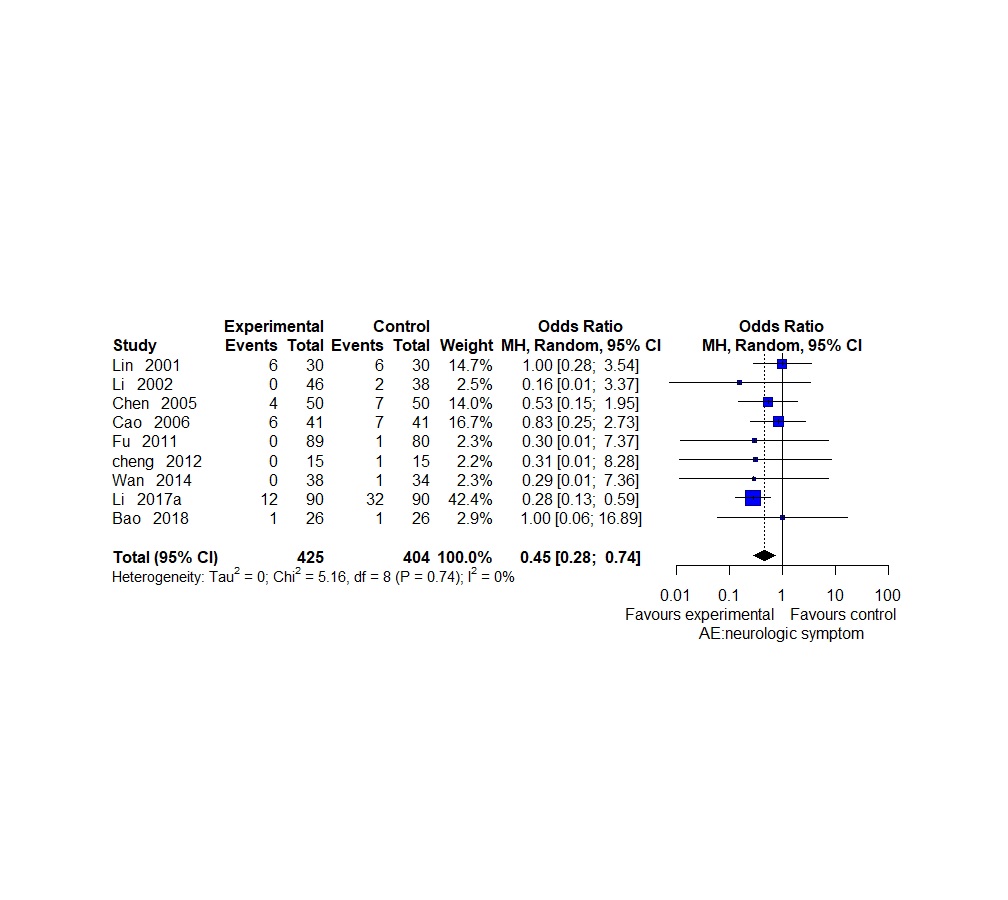

Supplement: Supplementary file 1 [file Image3.jpeg]

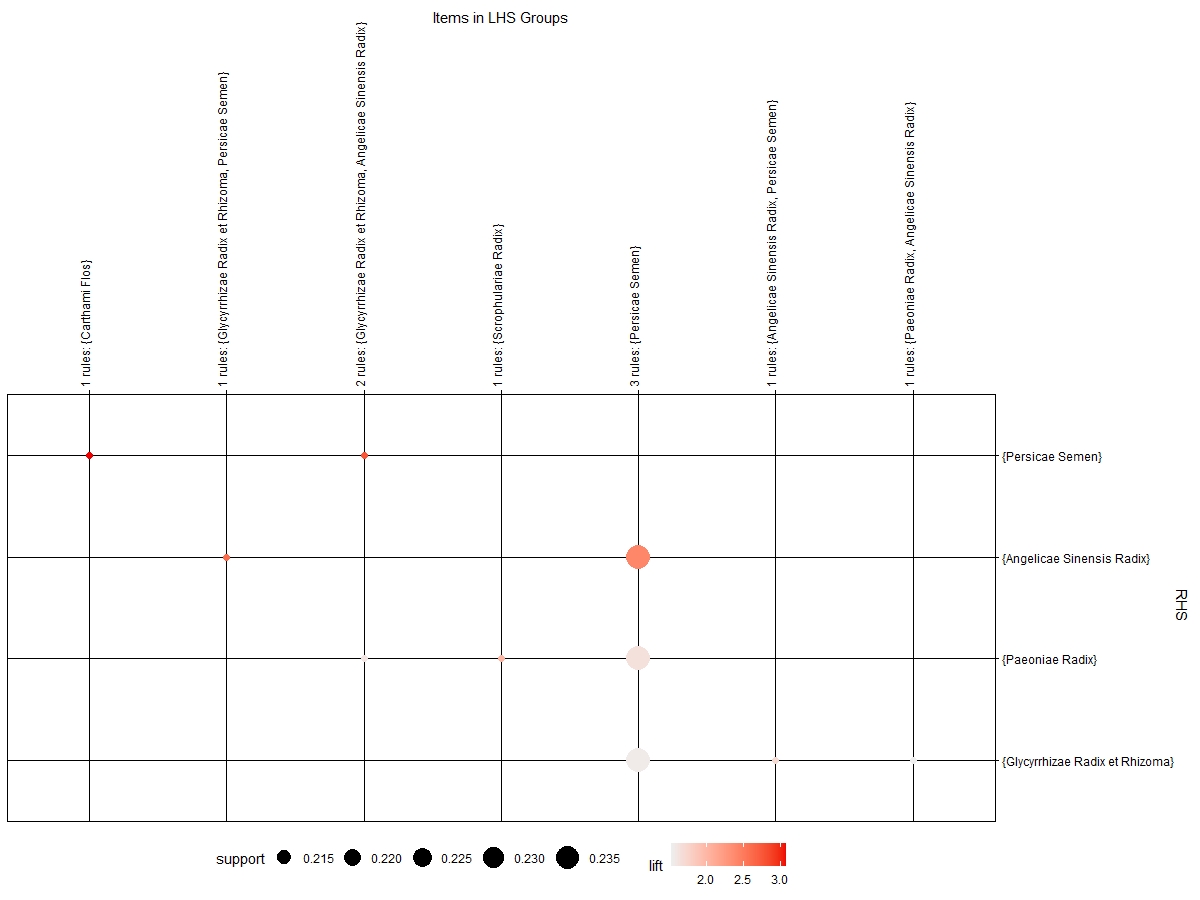

Supplement: Supplementary file 3 [file Image9.jpeg]

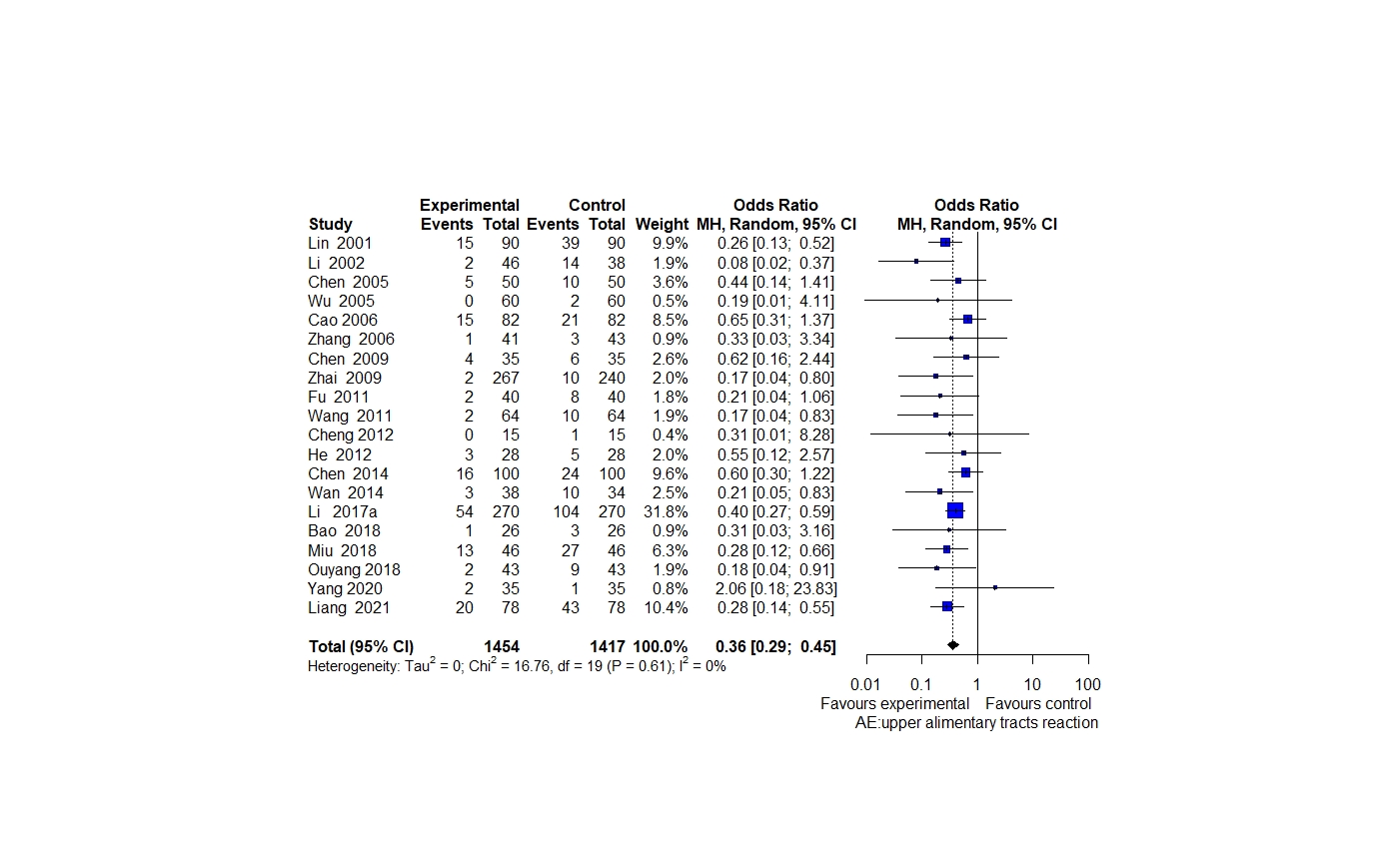

Supplement: Supplementary file 4 [file Image1.jpeg]

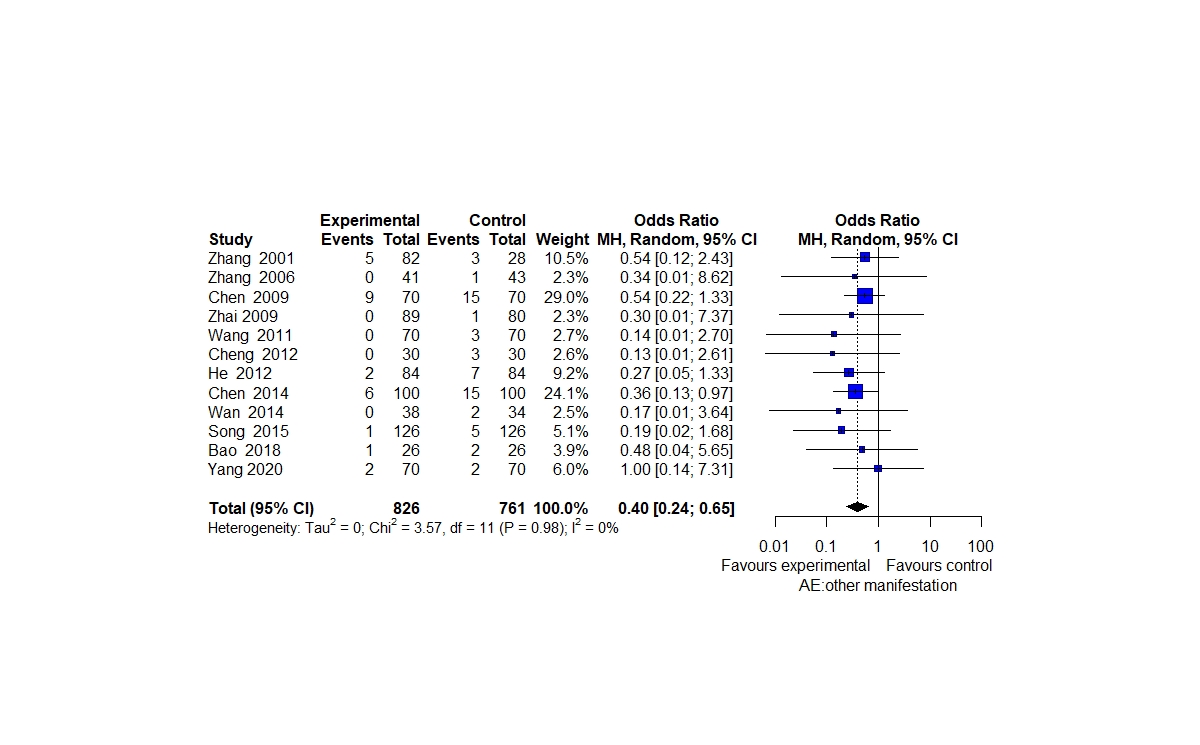

Supplement: Supplementary file 5 [file Image4.jpeg]

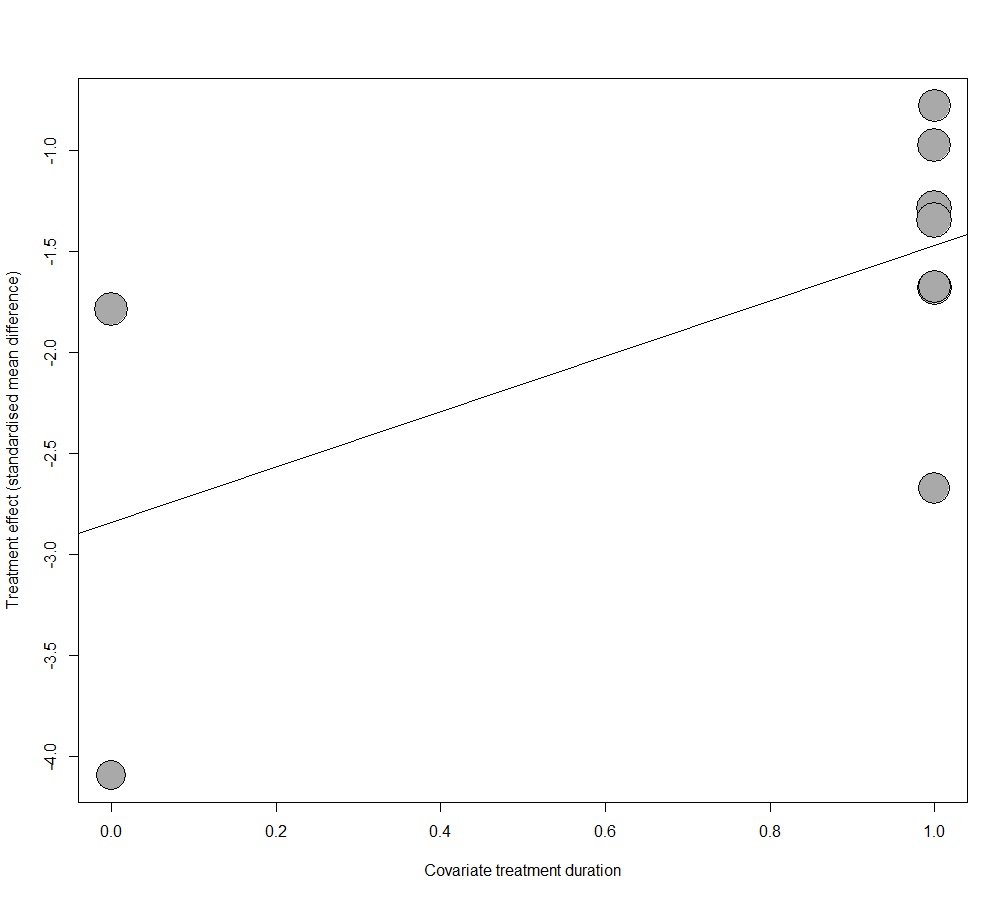

Supplement: Supplementary file 6 [file Image7.jpeg]

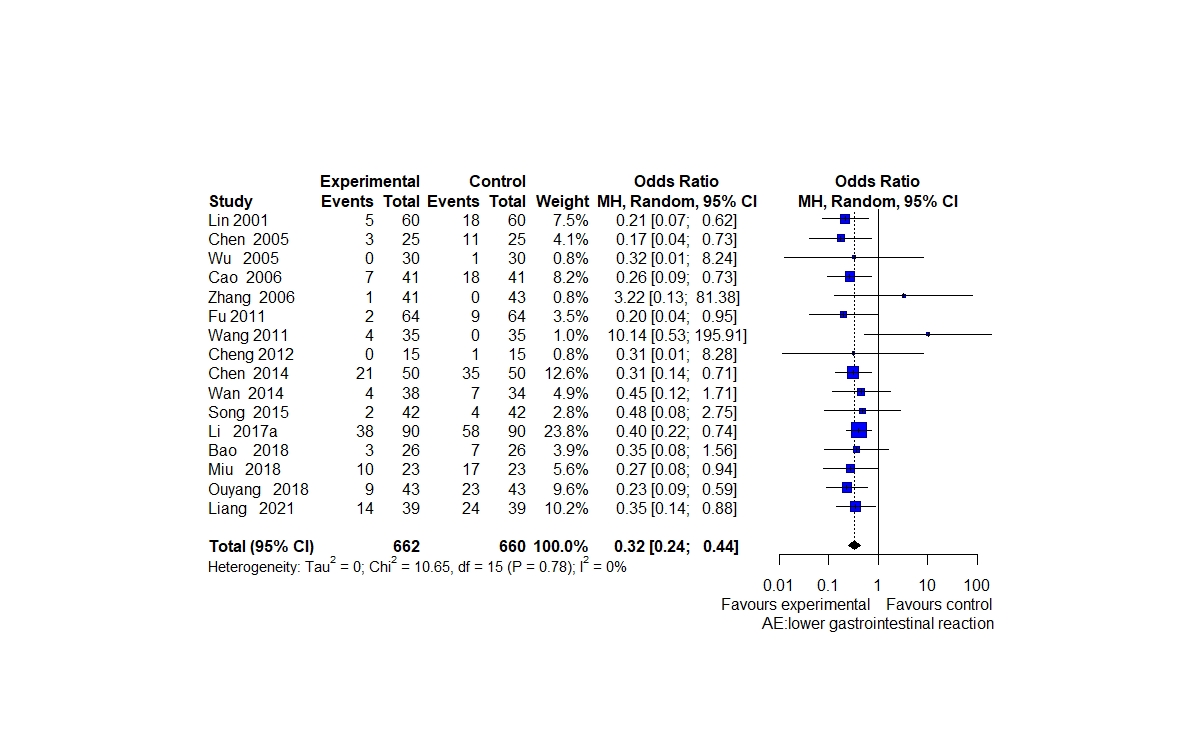

Supplement: Supplementary file 7 [file Image2.jpeg]

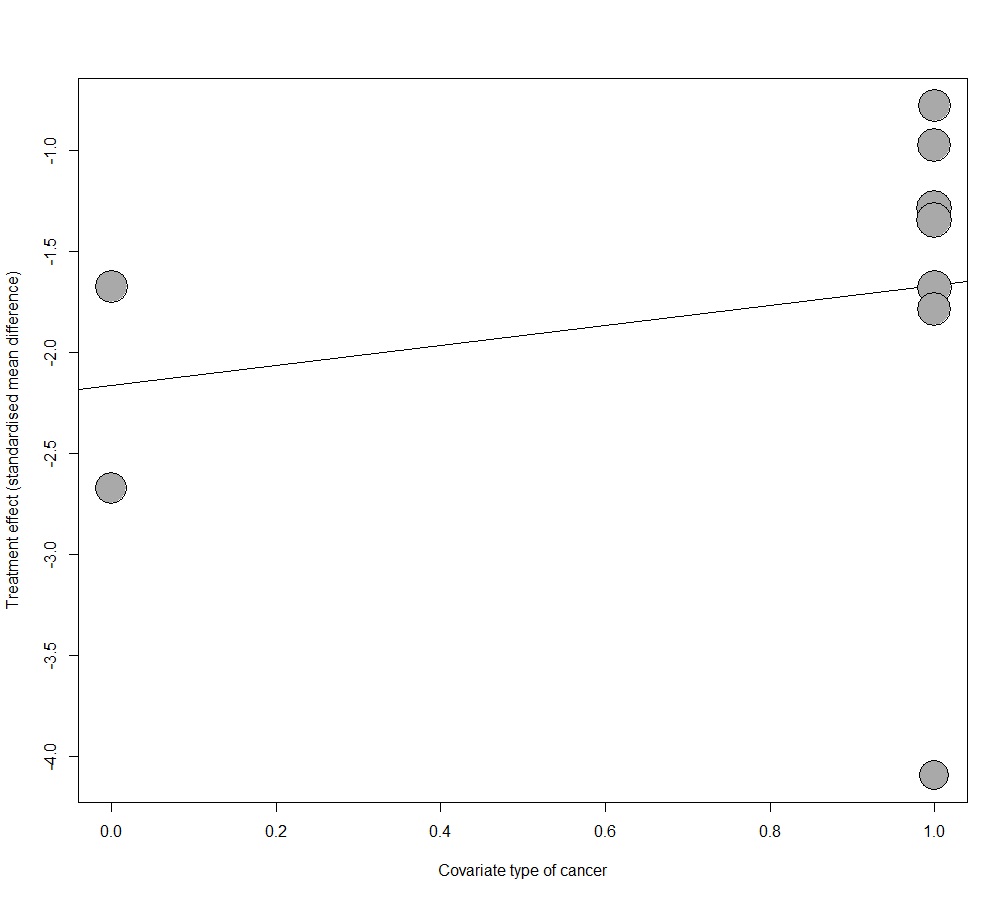

Supplement: Supplementary file 8 [file Image5.jpeg]

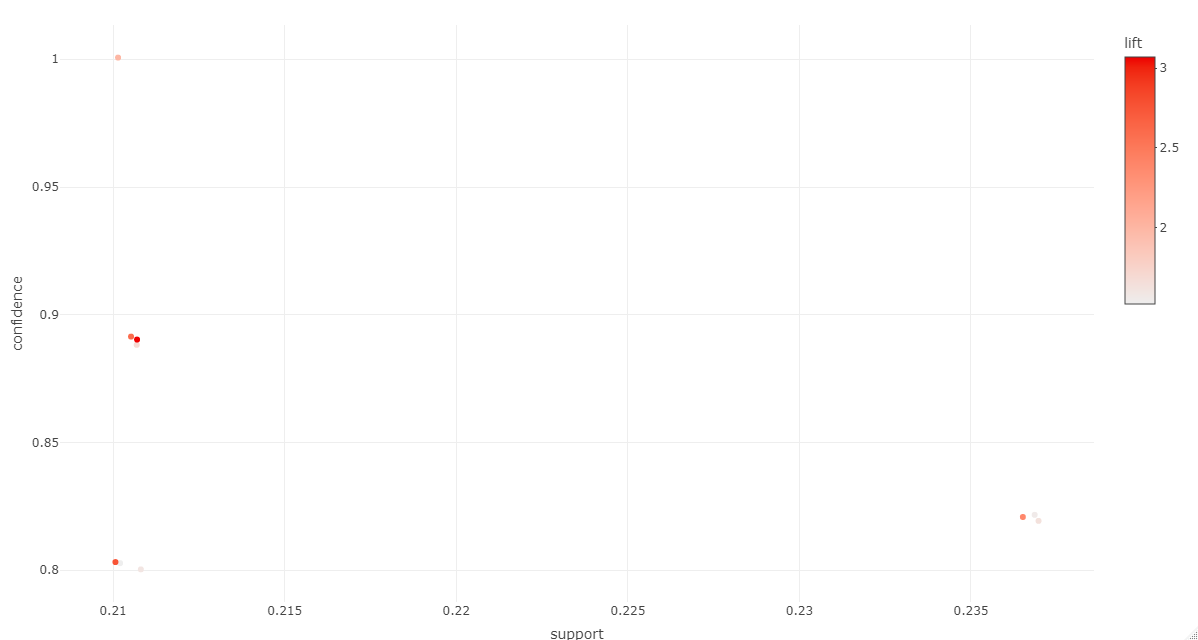

Supplement: Supplementary file 10 [file Image8.png]

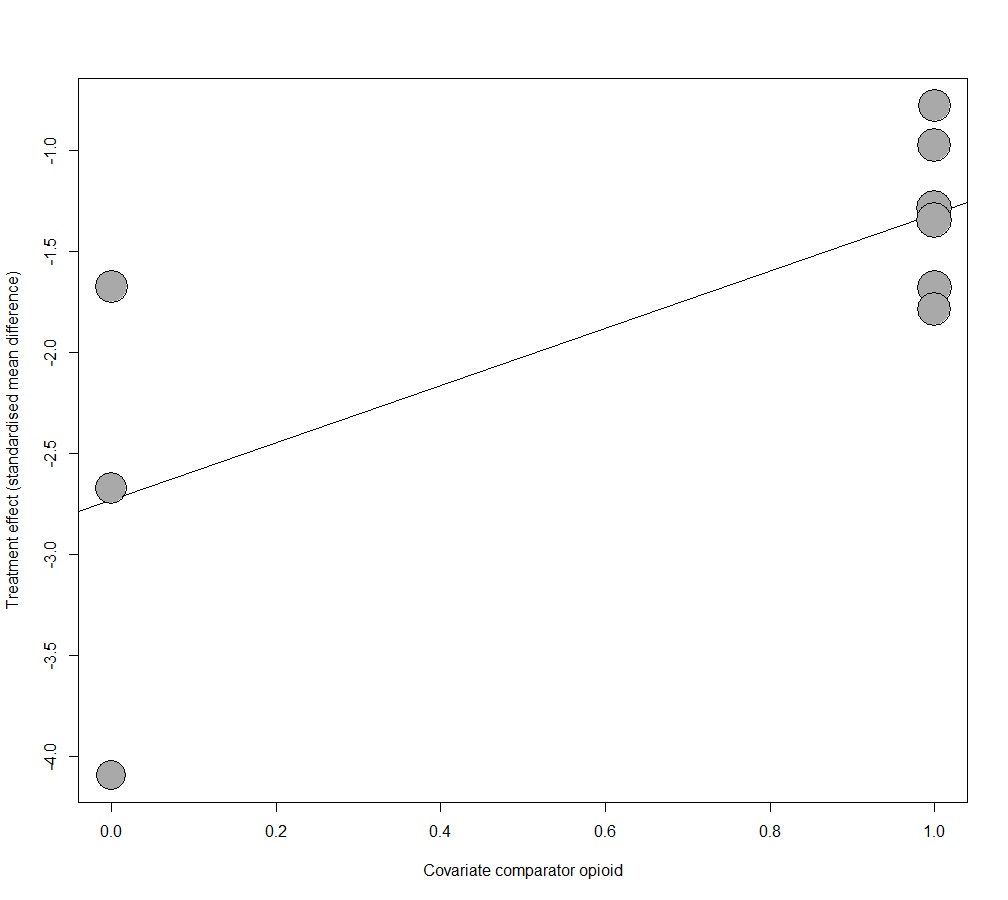

Supplement: Supplementary file 11 [file Image6.jpeg]
