# Supplementary material for: East Asian Herbal Medicine to Reduce Primary Pain and Adverse Events in Cancer Patients : A Systematic Review and Meta-Analysis With Association Rule Mining to Identify Core Herb Combination
Source: Front Pharmacol. 2022 Jan 17;12:800571. doi: 10.3389/fphar.2021.800571 (PMC8802093; doi:10.3389/fphar.2021.800571)
Supplement: Supplementary file 2 [file Table1.docx]

**Supplementary Table S1**. Search terms used in each database and the results of the search

**MEDLINE via PubMed**

|  | Searches | Results |
| --- | --- | --- |
| 1 | Pain[MeSH] OR Pain*[TIAB] OR analgesia OR analges* OR nocicept* OR neuroapth* | 1084669 |
| 2 | “Cancer pain”[TIAB] OR “Cancer patient”[TIAB] OR “Cancer patients”[TIAB] OR Neoplasms[MeSH] OR Neoplasms*[TI] OR Cancer*[TI] OR Tumor*[MeSH] OR Tumor*[TI] OR Carcinoma[MeSH] OR Carcinoma*[TI] OR Adenocarcinoma[MeSH] OR Adenocarcinoma*[TI] OR adenomatous[TI] OR Lymphoma[MeSH] OR lymphom*[TI] OR lymphedema*[TI] OR Sarcoma[MeSH] OR Sarcoma*[TI] OR ‘‘Antineoplastic agents’’[MeSH] OR antineoplas*[TI] OR ((adenom*[TI] OR adenopath*[TI]) AND malignant*[TI])) | 891784 |
| 3 | “Plants, Medicinal”[MeSH] OR “Drugs, Chinese Herbal”[MeSH] OR “Medicine, Chinese Traditional”[MeSH] OR “Medicine, Kampo”[MeSH] OR “Medicine, Korean Traditional”[MeSH] OR “Herbal Medicine”[MeSH] OR “Prescription Drugs”[MeSH] OR “traditional Korean medicine”[TIAB] OR “traditional Chinese medicine”[TIAB] OR “traditional oriental medicine”[TIAB] OR “Kampo medicine”[Title/abstract] OR herb*[TIAB] OR decoction*[TIAB] OR botanic*[TIAB] | 217216 |
| 4 | #1 AND #2 AND #3 | **9216** |

**EMBASE via Elsevier**

|  | Searches | Results |
| --- | --- | --- |
| 1 | 'neoplasms'/exp | 5,306,366 |
| 2 | neoplasm* OR cancer* OR carcino* OR malignan* OR tumor* OR tumour* | 6,603,790 |
| 3 | #1 OR #2 | 7,118,684 |
| 4 | 'pain'/exp | 1,453,298 |
| 5 | pain*:ti,ab | 1,106,196 |
| 6 | analgesia | 235,155 |
| 7 | analges* OR nocicept* OR neuropath*:ti,ab | 612,727 |
| 8 | #4 OR #5 OR #6 OR #7 | 2,127,650 |
| 9 | ‘medicinal plant’/exp OR ‘medicinal plant’ OR ‘herbaceous agent’/exp OR ‘herbaceous agent’ OR ‘chinese medicine’/exp OR ‘chinese medicine’ OR ‘kampo medicine’/exp OR ‘kampo medicine’ OR ‘kampo medicine (drug)’/exp OR ‘kampo medicine (drug)’ OR ‘korean medicine’/exp OR ‘korean medicine’ OR ‘herbal medicine’/exp OR ‘herbal medicine’ OR ‘prescription drug’/exp OR ‘prescription drug’ OR ‘oriental medicine’/exp OR ‘oriental medicine’ OR ‘alternative medicine’/exp OR ‘alternative medicine’ OR ‘complementary medicine’ OR ‘herb’/exp OR ‘herb’ OR ‘decoction’ OR ‘botanic’ | 592,999 |
| 10 | #3 AND #8 AND #9 | 10,583 |
| 11 | #3 AND #8 AND #9 AND [humans]/lim AND [clinical study]/lim | **4178** |

**CENTRAL**

|  | Searches | Results |
| --- | --- | --- |
| 1 | MeSH descriptor: [Neoplasms] explode all trees | 83140 |
| 2 | (neoplasm* or cancer* or carcino* or malignan* or tumor* or tumour*) | 242067 |
| 3 | #1 OR #2 | 249059 |
| 4 | MeSH descriptor: [Pain] explode all trees | 51905 |
| 5 | (Pain*):ti,ab,kw | 196999 |
| 6 | MeSH descriptor: [Analgesia] explode all trees | 8175 |
| 7 | (Analges* or nocicept* or neuropath*):ti,ab,kw | 81161 |
| 8 | #4 OR #5 OR #6 OR #7 | 227722 |
| 9 | MeSH descriptor: [Plants, Medicinal] explode all trees | 946 |
| 10 | MeSH descriptor: [Drugs, Chinese Herbal] explode all trees | 3674 |
| 11 | MeSH descriptor: [Medicine, Chinese Traditional] explode all trees | 1229 |
| 12 | MeSH descriptor: [Medicine, Kampo] explode all trees | 46 |
| 13 | MeSH descriptor: [Medicine, Korean Traditional] explode all trees | 33 |
| 14 | MeSH descriptor: [Herbal Medicine] explode all trees | 63 |
| 15 | MeSH descriptor: [Prescription Drugs] explode all trees | 108 |
| 16 | (“traditional Korean medicine” OR “traditional Chinese medicine” OR “Traditional oriental medicine” OR “Kampo medicine” OR herb* OR decoction* OR botanic*):ti,ab,kw | 19131 |
| 17 | #9 OR #10 OR #11 OR #12 OR #13 OR #14 OR #15 OR #16 | 20394 |
| 18 | # 3 AND #8 AND #17 in Trials | **1934** |

**CINAHL via EBSCO**

|  | Searches | Results |
| --- | --- | --- |
| 1 | TX pain OR MH pain | 521,816 |
| 2 | MH Neoplasms OR TX (neoplasm OR cancer OR tumor OR carcinoma OR lymphoma OR sarcoma) | 978,760 |
| 3 | MH (“Plants, Medicinal” OR “Drugs, Chinese Herbal” OR “Medicine, Chinese Traditional” OR “Medicine, Kampo” OR “Medicine, Korean Traditional” OR “Herbal Medicine” OR “Prescription Drugs”) OR TX (“traditional Korean medicine” OR “traditional Chinese medicine” OR “traditional oriental medicine” OR “Kampo medicine” OR herb* OR decoction* OR botanic*) | 121,789 |
| 4 | #1 AND #2 AND #3 | 8,698 |
| 5 | Limit #4 to ‘Clinical Trial’ and ‘Human’ | **274** |

**OASIS**

|  | Searches | Results |
| --- | --- | --- |
| 1 | 통증 AND 암 AND 한약 | **5** |

**KISS**

|  | Searches | Results |
| --- | --- | --- |
| 1 | 통증 AND 암 AND 한약 | **1** |

**RISS**

|  | Searches | Results |
| --- | --- | --- |
| 1 | 통증 AND 암 AND 한약 | **2** |

**KCI**

|  | Searches | Results |
| --- | --- | --- |
| 1 | 통증 AND 암 AND 한약 | **0** |

**CNKI**

|  | Searches | Results |
| --- | --- | --- |
| 1 | (SU='癌痛'+'癌性疼痛'+'癌因性疼痛'+'疼痛') AND (SU='癌'+'肿瘤'+'腺癌'+'肿瘤') AND (SU='中药'+'中医药'+'本草'+'汤'+'丸'+'散'+'方'+'颗粒'+'胶囊'+'自拟'+'止痛') AND (SU='随机') | **81** |

**Wanfang data**

|  | Searches | Results |
| --- | --- | --- |
| 1 | 题名或关键词:("癌痛" or "癌性疼痛" or "癌因性疼痛" or "疼痛") and 题名或关键词:("癌" or "肿瘤" or "腺癌" or "肿瘤") and 题名或关键词:("中药" or "中医药" or "本草" or "汤" or "丸" or "散" or "方" or "颗粒" or "胶囊" or "自拟" or "止痛") and 题名或关键词:("随机") | **802** |

**CiNii**

|  | Searches | Results |
| --- | --- | --- |
| 1 | (“Pain”) AND (“癌” OR “腺癌” OR “cancer” OR “neoplasm” OR “tumor”) AND (“traditional Korean medicine” OR “traditional Chinese medicine” OR “Traditional oriental medicine” OR “Kampo medicine” OR herb OR decoction OR botanic OR 漢方薬 OR ハーブ OR 散 OR 汤 OR 丸) | **754** |
