# Supplementary material for: East Asian Herbal Medicine to Reduce Primary Pain and Adverse Events in Cancer Patients : A Systematic Review and Meta-Analysis With Association Rule Mining to Identify Core Herb Combination
Source: Front Pharmacol. 2022 Jan 17;12:800571. doi: 10.3389/fphar.2021.800571 (PMC8802093; doi:10.3389/fphar.2021.800571)
Supplement: Supplementary file 9 [file Table2.docx]

**Supplementary Table S2 List of studies excluded after full text review (A total of 39 literatures)**

**Exclusion reason: Duplicated (N=1)**

1. Song H, Yin Y, Zhou L, Feng L. Ｒandomized Controlled Double-blind Clinical Study on Yishen Gukang Ｒecipe (益肾骨康方) Combined with Oxycodone Hydrochloride Sustained-release Tablets in the Treatment of Moderate to Severe Cancerous Pain with Kidney Deficiency and Blood Stasis Syndrome. Journal of Traditional Chinese Medicine. 2018;59(15):1300-1304.

**Exclusion reason: Inappropriate design (N=4)**

1. Song H, Yin Y, Zhou L, Feng L. Ｒandomized Controlled Double-blind Clinical Study on Yishen Gukang Ｒecipe (益肾骨康方) Combined with Oxycodone Hydrochloride Sustained-release Tablets in the Treatment of Moderate to Severe Cancerous Pain with Kidney Deficiency and Blood Stasis Syndrome. Journal of Traditional Chinese Medicine. 2018;59(15):1300-1304.

2. Liu Y, Xu K, Chen D. Clinical Observation of Xianlong Dingtong Yin on Osseous Metastasis Caused Pain. World Chinese Medicine. 2007;2(5):274-275.

3. Yang P, Dong N, Dong E. Xuantong Kangai Recipe (玄通抗癌方药) Combined with Boshengaining (博生癌宁) in Treating 40 Cases of Cancer Pain. Shaanxi Journal of Traditional Chinese Medicine. 2011;32(10):1343-1345.

4. Pan Y, Qian W, Liu L. Clinical Study on the Treatment of 39 Cases of Mild and Moderate Cancer Pain by Kangaizhitong Decoction. 2013;45(3):25-26.

**Exclusion reason: Not oral administration (N=6)**

1. Cai P, Li L, Hong H, et al. A Chinese medicine warm compress (Wen Jing Zhi Tong Fang), combined with WHO 3-step analgesic ladder treatment for cancer pain relief: A comparative randomized trial. Medicine (Baltimore). 2018;97(11):e9965. doi:10.1097/MD.0000000000009965

2. Tan H, Zhu S, Li T, et al. Safety and efficacy of Tongkuaixiao ointment in reliving cancer-induced pain: a multicenter double-blind placebo-controlled randomized trial. J Tradit Chin Med. 2016;36(6):695-700. doi:10.1016/s0254-6272(17)30001-8

3. Fu Q, Wang X. Clinical Observation on Cancer Pain Treated by Aitong Tincture (癌痛酊). Journal of Emergency in Traditional Chinese Medicin. 2012;21(3):488-489.

4. Zhou Y, Wang X, Zheng H, et al. Clinical Observation on Cancer Pain Treated by Huayu Sanhan Buxuetongluozhitong Ointment (化瘀散寒补血通络止痛膏). Acta Chinese Medicine and Pharmacology. 2015;43(4):118-120.

5. Mei S. Differentiation and Nursing of Cancer Pain of Deficiency and Blood Stasis Type. Liaoning Journal of Traditional Chinese Medicine. 2010;37(10):2043-2045.

6. Ye X, Lu D, Chen X, Li S, Chen Y, Deng L. A Multicenter, Randomized Double-Blind, Placebo-Controlled Trial o Shuangbai San for Treating Primary Liver Cancer Patients With Cancer Pain. J Pain Symptom Manage. 2016:51(6):979-986. doi:[10.1016/j.jpainsymman.2015.12.330](https://doi.org/10.1016/j.jpainsymman.2015.12.330)

**Exclusion reason: Not related to cancer pain (N＝11)**

1. Kaku H, Kumagai S, Onoue H, et al. Objective evaluation of the alleviating effects of Goshajinkigan on peripheral neuropathy induced by paclitaxel/carboplatin therapy: A multicenter collaborative study. Exp Ther Med. 2012;3(1):60-65. doi:10.3892/etm.2011.375

2. Abe H, Kawai Y, Mori T, et al. The Kampo medicine Goshajinkigan prevents neuropathy in breast cancer patients treated with docetaxel. Asian Pac J Cancer Prev. 2013;14(11):6351-6356. doi:10.7314/apjcp.2013.14.11.6351

3. Kono T, Hata T, Morita S, et al. Goshajinkigan oxaliplatin neurotoxicity evaluation (GONE): a phase 2, multicenter, randomized, double‑blind, placebo‑controlled trial of goshajinkigan to prevent oxaliplatin‑induced neuropathy. Cancer chemotherapy and pharmacology. 2013;72(6). doi:10.1007/s00280-013-2306-7

4. Chen C, Lin L, Zhang E. Standardized treatment of Chinese medicine decoction for cancer pain patients with opioid-induced constipation: a multi-center prospective randomized controlled study. Chin J Integr Med. 2014;20(7):496-502. doi:10.1007/s11655-014-1864-9

5. Zhu G, Liu F, Zhu Z, et al. Clinical Observation of Buzhong Yiqi Decoction in Treating Cancer-induced Fatigue Between Chemotherapy of Gastric Cancer. Modern Hospital. 2016;16(8):1169-1171.

6. Zhu L, Mao Z, Yao Q, Guo L, Shen K. Clinical effect of Wenjing-Quyu-Juanbi Prescription combined with methylcobalamin in treatment of chemotherapy-induced peripheral neuropathy. Journal of Shanghai Jiao Tong University. 2019;39(7):778-782.

7. Li Y, Cui H, Huang J, et al. Clinical Study of Jiawei Huangqi Guizhi Wuwu Decoction (加味黄芪桂枝五物汤) in Preventing and Treating Peripheral Neuro-sensory Toxicity Caused by Oxaliplatin. Chinese Journal of Integrative Medicine, 2006;12(1):19-23.

8. Yang L, Zhang S. Observation on the short-term curative effect of Qiankun Capsule (乾坤胶囊) combined with chemotherapy in the treatment of advanced malignant tumors. Journal of Hebei Traditional Chinese Medicine and Pharmacology. 2001;16(3):21-22.

9. Zhang K. Clinical Observation on Treatment of 80 Cases of Liver Cancer with Gan Fu recipe. China Foreign Medical Treatment. 2011(14):58-59.

10. Liu Y, Zhu G, Han L, et al. Clinical Study on the Prevention of Oxaliplatin-Induced Neurotoxicity with Guilongtongluofang: Results of a Randomized, Double-Blind, Placebo-Controlled Trial. Evid Based Complement Alternat Med. 2013;2013:541217. doi:10.1155/2013/541217

11. Mok TS, Yeo W, Johnson PJ, et al. A double-blind placebo-controlled randomized study of Chinese herbal medicine as complementary therapy for reduction of chemotherapy-induced toxicity. Ann Oncol. 2007;18(4):768-774. doi:10.1093/annonc/mdl465

**Exclusion reason: Not disclosed to herb ingredients (N=11)**

1. Chen C, Sun G, Tang W, et al. Clinical obsevration of the efficacy and safety of “Guishen analgesic mixture” in treatment of cancer pain. Chinese New Drugs Journal. 2000;9(3):196-200.

2. Lu R, Zhang C. Clinical Double Blind and Simulated Tests on Guiseng Zhitong Mixture. Annals of Yunnan Traditional Chinese Medicine and Chinese Medicine. 2001;22(1):5.

3. Zhong J, Que T, Zhang H, Wei M, Lin Z. Clinical Evaluation of the analgesic effect of comdrugcide on cancer pain. Chinese Journal of Pain Medicine. 88-91;2004(10):2.

4. Shi H, Liu X, Shi K, Huang D. Clinical observation on the treatment of 90 cases of pain caused by malignant tumor with Aitongling. Guiding journal of TCM. 2005;11(2):21-22.

5. Luo X. Analysis of Curative Effect of Jintong Oral Liquid in Treating Tumor Pain. CHinese Medicine Guide. 2008;6(16):474-475.

6. Li Q, Liu S, Li S. Clinical Observation on Tonglingzhi Capsule in Treating Cancer Pain. Practical Integrated Traditional Chinese and Western Medicine Clinic. 2010;10(4):11-12.

7. Ma C. Twelve Wei Yiliu Capsules Combined with Indomethacin Suppository Treating Mild to Moderate Pain in Malignant Tumor of Qi Deficiency and Blood Stasis Type. Chinese Journal of Experimental Traditional Medical Formulae. 2010;16(12):189-190.

8. Wu S, Wu B. Clinical observation of Xihuang capsules and morphine hydrochloride sustained-release tablets treating the medium-term and advanced lung cancerous pain. Chinese Medicine and Pharmacy. 2012;2(3):21-22.

9. Zou S, Gao F. Dahuang Lidan Capsule Combined Morphine Controlled- release Tablets Treatment of Moderate to Severe Cancer Pain Randomized Controlled Study. Journal of Practical Traditional Chinese Internal Medicine. 27(5):104-105.

10. Chi Y. Clinical effect observation of Xihuang capsule combined with morphine hydrochloride sustained-release tablets in the treatment of middle and late stage lung cancer pain. Xiandai Yangsheng. 2017;16:141.

11. Yang H, Han Z, He M, Wu J, Zou X, Zhang C. Effect Analysis of Chinese Herb on the Remission of Pain in Colorectal Cancer Patients. Anti-tumor Pharmacy. 2018;8(1):57-59.

**Exclusion reason: Quasi RCT (N=6)**

1. Gao T, Shi H, Liu Z, Xu L, Zhang X, Tang J. Clinical Observation on 26 Cases of Cancer Pain Treated by Kangsaide Zhitong Decoction. Journal of Traditional Chinese Medidicine. 1998;39(7):416-417.

2. Bai G, Lu Y, Huang D. Clinical Observation on Treatment of Moderate and Severe Cancer Pain with Fuzheng Yiai Decoction and Western Medicine. Journal of New Chinese Medicine. 2010;42(8):78-79.

3. Cong Y, Sun K, He X, et al. A Traditional Chinese Medicine Xiao-Ai-Tong Suppresses Pain through Modulation of Cytokines and Prevents Adverse Reactions of Morphine Treatment in Bone Cancer Pain Patients. Mediators Inflamm. 2015;2015:961635. doi:10.1155/2015/961635

4. Yang L. Clinical Observation of Fuzi Lizhong Decoction on 45 Cases of Cancer Pain Caused by Digestive Tract Tumor. Journal of Clinical Medicine. 2017;4(84):16614-16616.

5. Xu Z, Ma W. Study on the curative effect of Xuefu Zhuyu Decoction with routine western medicine on cancer pain with Qi deficiency and blood stasis. J Community Med. 2019;17(16):1005-1007.

6. Wang X, Li J. Clinical Observation on the Treatment of Moderate and Severe Cancer Pain by Self-made Jijizhitong Decoction Combined with Oxycodone Hydrochloride. JETCM. 2019;28(2):331-334.
